# Coordination Between Partial Robotic Exoskeletons and Human Gait: A Comprehensive Review on Control Strategies

**DOI:** 10.3389/fbioe.2022.842294

**Published:** 2022-05-25

**Authors:** Julio S. Lora-Millan, Juan C. Moreno, E. Rocon

**Affiliations:** ^1^ Centre for Automation and Robotics, Consejo Superior de Investigaciones Científicas-Universidad Politécnica de Madrid, CSIC-UPM, Madrid, Spain; ^2^ Electronic Technology Department, Universidad Rey Juan Carlos, Madrid, Spain; ^3^ Neural Rehabilitation Group, Cajal Institute, Spanish National Research Council (CSIC), Madrid, Spain

**Keywords:** unilateral robotic exoskeleton, single-joint powered orthosis, gait assistance, coordination strategies, state-of-the-art

## Abstract

Lower-limb robotic exoskeletons have become powerful tools to assist or rehabilitate the gait of subjects with impaired walking, even when they are designed to act only partially over the locomotor system, as in the case of unilateral or single-joint exoskeletons. These partial exoskeletons require a proper method to synchronize their assistive actions and ensure correct inter-joint coordination with the user’s gait. This review analyzes the state of the art of control strategies to coordinate the assistance provided by these partial devices with the actual gait of the wearers. We have analyzed and classified the different approaches independently of the hardware implementation, describing their basis and principles. We have also reviewed the experimental validations of these devices for impaired and unimpaired walking subjects to provide the reader with a clear view of their technology readiness level. Eventually, the current state of the art and necessary future steps in the field are summarized and discussed.

## 1 Introduction

Robotic exoskeletons are wearable mechanisms capable of augmenting, restoring, or assisting the function of human limbs by acting in parallel (Pons, 2008). This technology can be applied in many fields, ranging from industrial (Fox et al., 2019) or military (Chu et al., 2006) domains, where the user is empowered to perform a heavy task, to space teleoperation (Lovasz et al., 2017) or health care (Cardona et al., 2020). More specifically, two of the fields in which lower-limb robotic exoskeletons have shown promising results are rehabilitation (Louie and Eng, 2016; Holanda et al., 2017) or assistance (Yan et al., 2015a) of human gait.

In this context, significant research efforts have been made to develop and improve wearable robotic devices that provide proper assistance during gait. A considerable amount of works have addressed different aspects of the design, control strategies, and experimental validation of these devices. To understand and organize the large body of information on this topic, several authors have reviewed the development of lower-limb robotic exoskeletons from multiple perspectives [see (Sanchez-Villamañan et al., 2019) for a complete review of compliant actuators currently used in robotic exoskeletons or (Pinto-Fernandez et al., 2020) for a detailed analysis of performance metrics, for instance].

In terms of control strategies, Tucker et al. provided an overview of the different control strategies for lower-limb robotic prostheses and orthoses and introduced a three-level paradigm controller development (Tucker et al., 2015). This same paradigm was recently updated and completed by Baud et al., who systematically analyzed the control strategies of lower-limb exoskeletons by dividing them into functional units (Baud et al., 2021). Considering these three-level paradigms, Ma et al. performed a deeper review of the high-level controllers responsible for the voluntary control of robotic devices (Ma et al., 2019), while Miscon et al. focused on middle-level controllers, in particular, joint trajectory generation for robotic exoskeletons (Miskon and Yusof, 2014). Low-level controllers responsible for direct actuator control were reviewed by Meng et al. (2015). The review proposed by Yan et al. also focused on control strategies for lower-limb exoskeletons, more specifically, on strategies that assisted user’s gait (Yan et al., 2015a). Similarly, Li et al. also reviewed control strategies for lower-limb exoskeletons but centered on rehabilitation purposes (Li et al., 2021).

Considering the number of actuated joints, robotic exoskeletons can be classified as single-joint or multi-joint (former, if they actuate over one unique joint; latter if more than one joint is assisted) (Yan et al., 2015a). Some authors have focused their reviews on single-joint exoskeleton robots. For instance, ankle robots have been reviewed by Mills et al. (2010) and also by Shi et al. (2019), while Chen et al. analyzed both knee (Chen et al., 2019) and hip (Chen et al., 2020) devices.

In contrast with complete lower-limb exoskeletons, which act over the hips and knees (even ankles) of both limbs, partial exoskeletons act partially over the locomotor system of the wearer by exclusively assisting one joint (single-joint exoskeletons) or one leg of the user (unilateral exoskeletons). Although they present several advantages [they are simpler and lighter than bilateral devices (Baud et al., 2021) and can target the specific function of the assisted joint during gait (Yan et al., 2015a)], they need to ensure proper interjoint coordination, especially with non-actuated joints. Unlike complete exoskeletons, which can impose the appropriate coordination between joints and legs, partial exoskeletons cannot actuate globally in the entire locomotor system; therefore, this interjoint coordination needs to be resolved by the controller of the device. Such coordination requires a dual interaction with humans: cognitive and physical. Proper delivery of the assistance is required to ensure that the user can benefit from the exoskeleton’s assistance, but also, a predictable timing that matches the user’s pre-estimation is required to achieve the exoskeleton’s embodiment. This would lead wearers to assimilate the robot’s action; so the exoskeleton would not be used as a tool but as a part of the user’s body (Li et al., 2021).

Understanding strategies that achieve proper coordination of human and robotic systems has become crucial, especially for those devices which aim to rehabilitate or assist the gait of impaired walking users. Although coordinated operation is crucial for the proper operation of a partial robotic exoskeleton, it has not yet been systematically analyzed in previous works. The main objective of this paper is to perform a comprehensive analysis of the different control strategies that are used to coordinate the action of partial exoskeletons with the user’s gait to ensure proper interjoint coordination. We have also included those strategies initially developed for bilateral or complete exoskeletons, that could be used to synchronize the action of partial exoskeletons. We have paid particular attention to the assessments and validations that the authors have carried out with their devices and strategies. The reported effects of each work on impaired or unimpaired walking subjects have also been summarized in this review. The content of this paper is organized as follows. Section 2 reports the literature search methodology that we followed. Section 3 describes the state of the art, organized into the five main coordination strategies that we identified. Finally, section 4 and section 5 discuss and conclude the main findings of this review.

## 2 Literature Search Methodology

We conducted a literature search using two different databases: Scopus and Web of Science from January 2004 until December 2021. To obtain results that cover the coordination issue between humans and robots, especially in unilateral or single-joint devices, we used the following search query in the title, abstract, and keywords:

Topic = {leg OR hip OR knee OR ankle OR foot OR [lower AND (limb* OR extremity OR body)]} AND Topic= (power* OR robot*) AND Topic = [ortho* OR exoskeleton* OR (wearable robot*) OR (portable robot*) OR (robot* suit) OR (robot exosuit)] AND Topic= (control* OR validation* AND Topic= (coord* OR unilateral OR (mono joint) OR (single joint) OR ((sound OR impaired OR paretic) AND (leg OR limb)) OR (hemip*))

Inclusion criteria for this review were as follows:

1) English full-text journal articles or conference proceedings.
2) Studies related to the design and control of a unilateral or single-joint robotic exoskeleton to assist gait.
3) Studies that involved a bilateral exoskeleton whose control strategy could be directly applied to the unilateral or single-joint paradigms.
4) Studies with a description of the experimental validation and assistance results of the devices mentioned above.

Exclusion Criteria Included

1) Documents that only described the mechanical structure of the device or the design of actuators or new materials intended for gait assistance.
2) Documents that described prosthesis or passive and uncontrolled orthosis.
3) Documents that lacked complete methods, results, or discussion sections.

**FIGURE 1:**
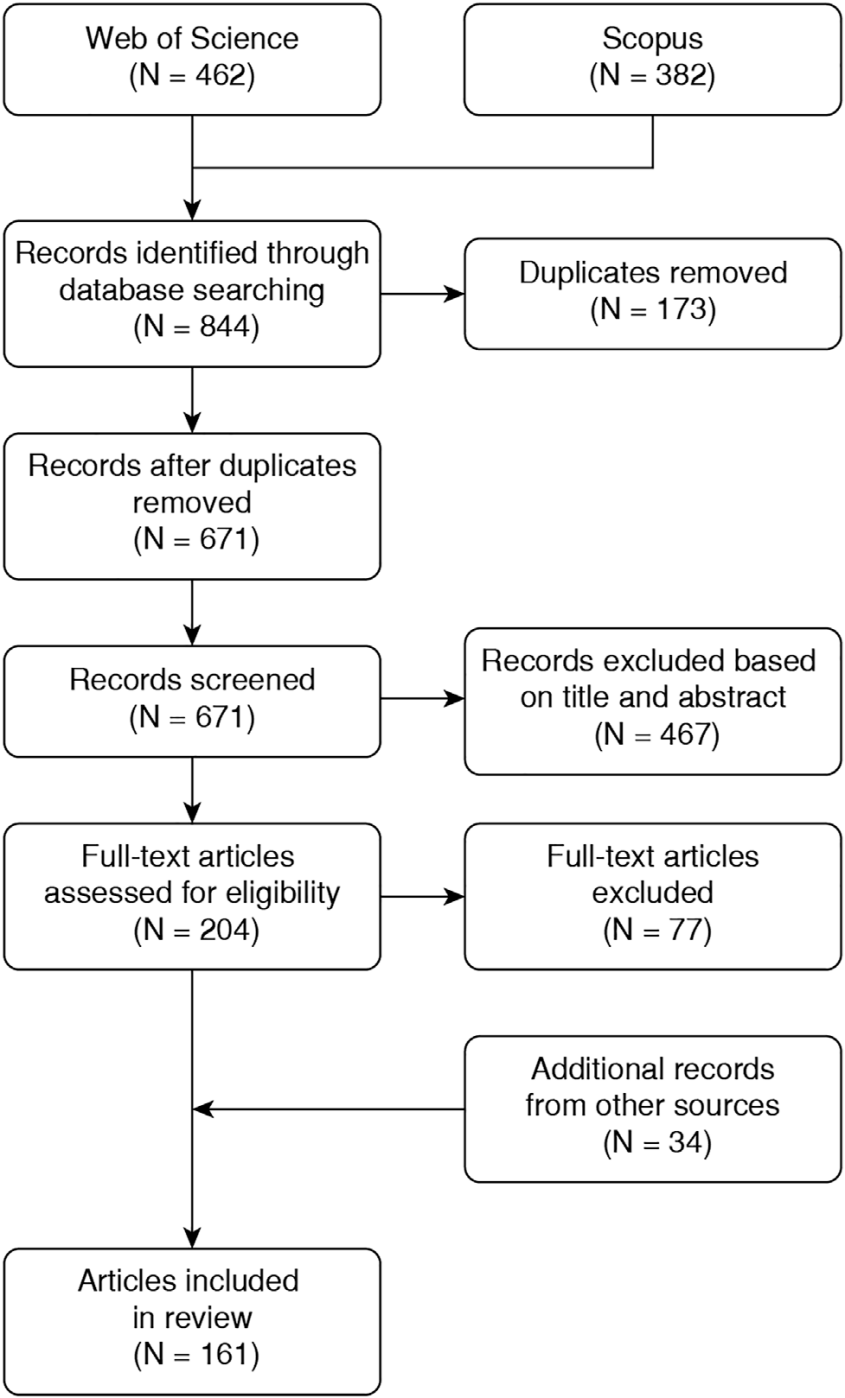
Flow diagram of the literature search methodology for document selection.

The initial number of articles (844) was reduced to 671 after looking for duplicated documents. After checking the title and abstract, we discarded 467 papers and 204 were selected for full-text reading. Based on the authors’ experience and the bibliography of the reviewed articles, 34 documents that were not included in the initial search were also considered for full-text reading. Then, we selected the 161 documents that fulfilled the inclusion criteria to be reported in this review. Figure 1 shows the flow diagram of the literature search and the document selection procedure.

## 3 State of the Art

Our analysis of the selected documents was focused on two main aspects: 1) coordination strategies with the actual user’s gait and 2) experimental validation of the devices regarding the subjects (number and pathology) and obtained results. The control strategies were analyzed regardless of the device’s morphology or actuation principle, based on the working principles that ensure proper interjoint coordination between the robotic exoskeleton and the user’s gait. The individual details of each reviewed paper are shown in Supplementary Table S1.

**FIGURE 2:**
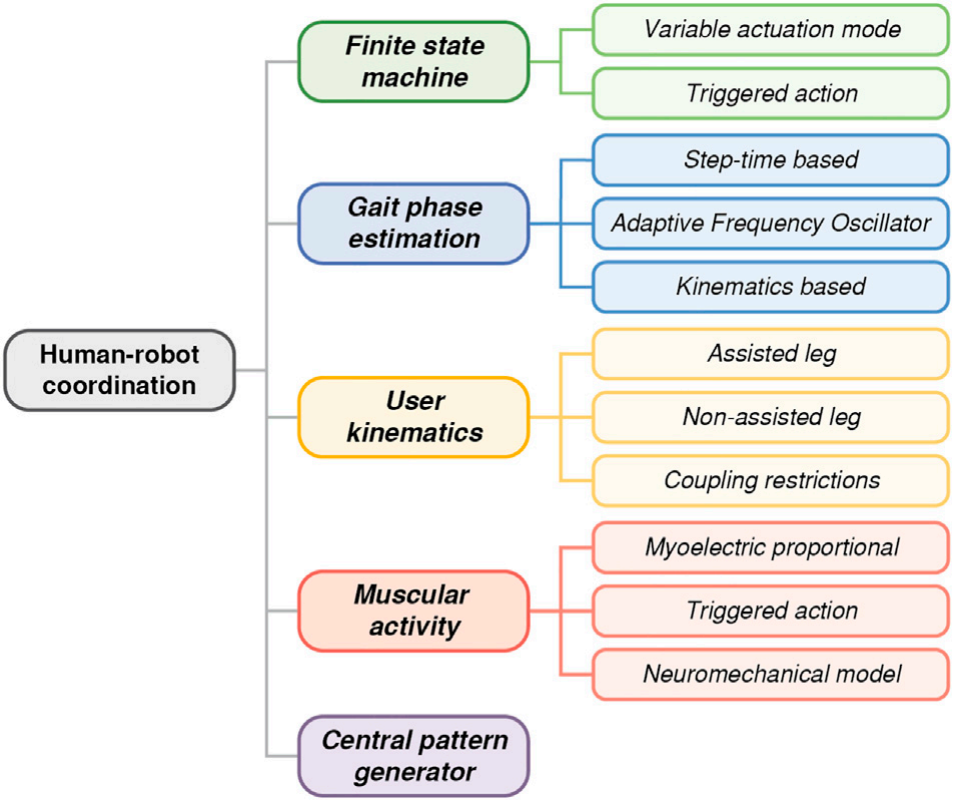
Classification of the different kinds of human-robot coordination strategies identified throughout the systematic review.

Across the literature, we have identified five strategies to synchronize the action of wearable robotic devices with human gait. The most extended methodology exploits the cyclic nature of human gait by using a finite state machine. Some authors leverage this cyclic property to estimate the continuous gait phase, which is a function that increases monotonically from 0 to 100% between consecutive heel strikes, and use this variable for the timing of the robot assistance. Other methodologies are based on the activity generated by the user, relying on either the movement itself or the muscle activity. Finally, mathematical tools have been developed, as in the case of central pattern generators that simulate the full gait dynamics of a subject. Figure 2 shows a diagram of the proposed classification.

### 3.1 Coordination Based on Finite State Machine

**FIGURE 3:**
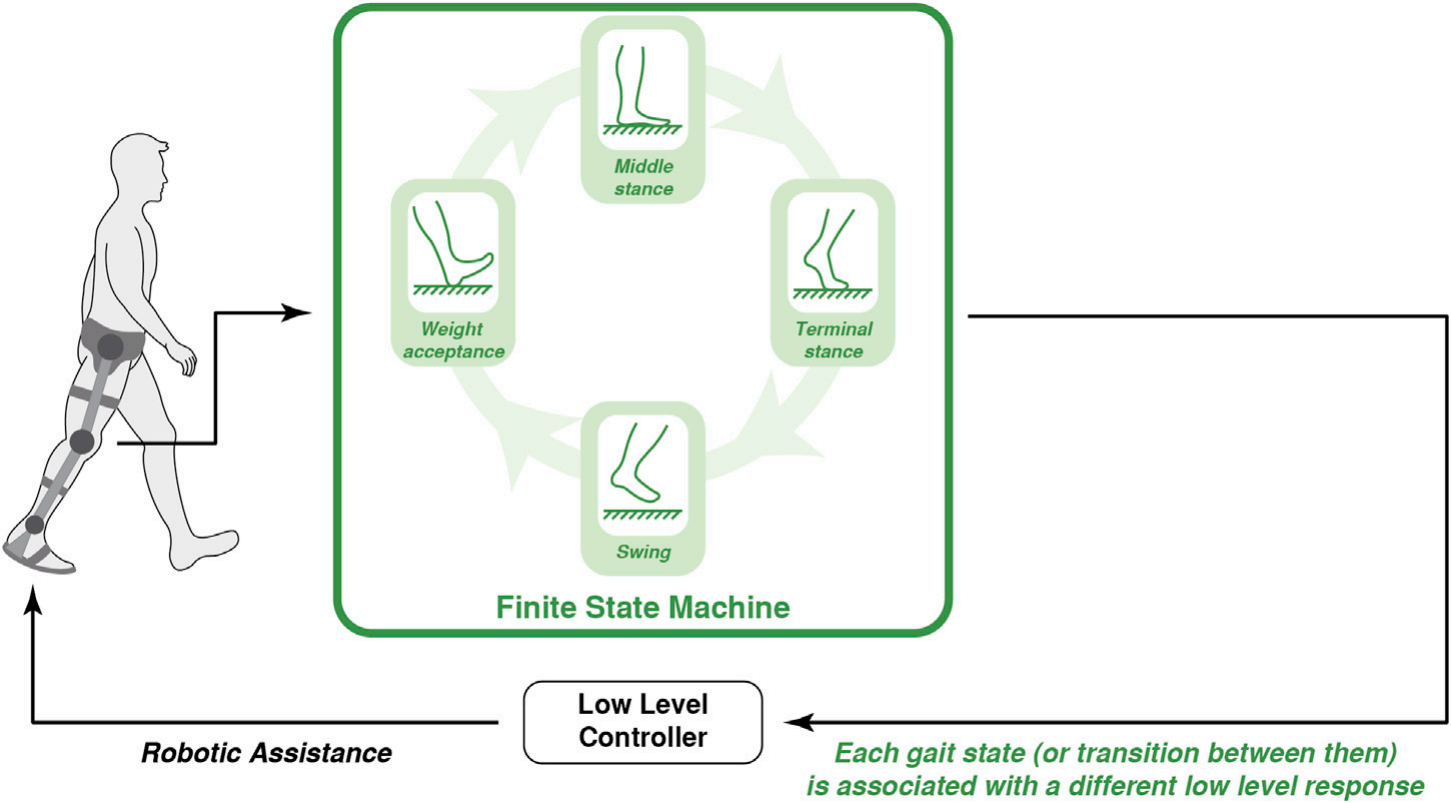
Conceptual representation of the robot-gait coordination based on a finite state machine: each gait state or the transitions between them are associated to a different action in the exoskeleton.

Since human gait is a continuous repetition of the same states and events, some authors have taken advantage of this cyclic nature to coordinate the action of robotic exoskeletons with human locomotion (Blaya and Herr, 2004; Kawamoto and Sankai, 2005; Zhang et al., 2017). Due to its simplicity, authors have used this strategy since the 1960s (Popovic et al., 1991, 1995; Tomovic and McGhee, 1966). Figure 3 represents the conceptual description of this coordination strategy between robot and human gait. This coordination relies on detecting key events and consequent gait division into states. These key-events are related to the joint kinematics (maximum and minimum of the joint angle or the angular velocity) or events about the foot’s contact with the floor (heel-strike, flat-foot, toe-off, etc.,). A common method to follow the sequence of gait events is the use of a finite state machine (FSM) based on angular sensors (such as gyroscopes or potentiometers) (Di Natali et al., 2019; Xia et al., 2020) or pressure sensors (such as insole force resisting sensors—FSR or foot switches) (Blaya and Herr, 2004; Kawamoto and Sankai, 2005; Shorter et al., 2011).

Within this strategy, robot coordination can be achieved by different approaches. Some authors triggered a specific action when a key-event was detected (Kawamoto and Sankai, 2005; Zhang et al., 2017), while others changed the actuation mode of the robot depending on the current gait states determined by the FSM (Blaya and Herr, 2004).

#### 3.1.1 Actuation Mode Depending on the Gait Subphase

The contact of each foot with the floor can be used to divide the gait cycle of each leg into states, defining the stance and swing states for the contact and noncontact stages, respectively. Several authors tailored the actuation mode of their system according to the current gait state in different ways, modifying the impedance of the actuators (Blaya and Herr, 2004; dos Santos et al., 2017), their stiffness (Shamaei et al., 2013; 2014a; 2014b, 2015) or the torque levels (Horst, 2009; Forrester et al., 2016).

The most extended method was to modulate the impedance level of the robotic joint according to the biomechanical requirements of the human joint during each gait state. Typically, a high-impedance model was applied during stance to assist weight acceptance, while a low-impedance model allowed free movement of the leg during the swing state (Blaya and Herr, 2004; Chinimilli et al., 2020; dos Santos et al., 2017; Kim et al., 2015; Shamaei et al., 2013; Villa-Parra et al., 2017; El Zahraa Wehbi et al., 2017; Xu et al., 2019; Zhou et al., 2016).

A different approach consisted of adjusting the torque assistant profile depending on the gait state derived from biomechanical models. Thus, ankle exoskeletons applied plantarflexion torque during weight loading to prevent foot drop, and applied plantarflexion torque during preswing and dorsiflexion torque during swing phase to prevent toe drag (Kim et al., 2011, 2020; Roy et al., 2013; Forrester et al., 2016; Choi et al., 2018). Meanwhile, knee exoskeletons reinforced the extension of the joint during stance and guide the movement during the swing phase of the gait (Horst, 2009; Wong et al., 2012; Stein et al., 2014; Arazpour et al., 2016; Lee et al., 2020; Liu and Wang, 2020).

In other cases, this state-dependent actuation mode was exploited by elastic actuators to store energy during the loading phase and release it afterwards to assist the movement of the joint. Thus, body inertia during the stance phase collaborated to compress an elastic actuator that was decompressed during the swing, releasing the elastic energy and assisting the movement of the leg (Ward et al., 2011; Di Natali et al., 2019).

#### 3.1.2 Action Triggered by the Detection of Key-Events

Some exoskeletons based the timing of their assistance on detecting certain key-events, typically the heel strike. When this event was detected, the device triggered the application of a predefined position and velocity (Kawamoto et al., 2009), torque (Shorter et al., 2011; Sridar et al., 2018, 2020), or work profiles (Mooney et al., 2014a, 2014b, 2014c; Mooney and Herr, 2016). In addition to the heel strike, other foot events could also be used to trigger a different assistive profile, such as push-off (Acosta-Sojo and Stirling, 2022) or flat-foot events (Lerner et al., 2018). A set of different joint events can also be used to trigger different assistive profiles in different gait states (Gomez-Vargas et al., 2021; Lerner et al., 2017b, 2017a; Xia et al., 2020; Yeung et al., 2017, 2021).

To improve the efficacy of the assistance, some authors scaled the duration of the reference profile to the duration of previous steps (Beyl et al., 2011; Knaepen et al., 2014; Jackson and Collins, 2015, 2019; Lim et al., 2015; Witte et al., 2015; Galle et al., 2017; Steele et al., 2017; Zhang et al., 2017; Ma et al., 2018; Malcolm et al., 2018; Wei et al., 2019; Bacek et al., 2021). By doing so, the assistive profile was adapted to different gait velocities and could be used independently of the individual gait features of the user. However, the trigger of these profiles was still limited to the event detection moment.

### 3.2 Coordination Based on the Real-Time Estimation of the Continuous Gait Phase

For the coordination between wearable robots and human gait, several authors have opted to use the concept of continuous gait phase in their control paradigms (Ronsse et al., 2011; Giovacchini et al., 2015; Awad et al., 2017b; Jin et al., 2017). The gait phase is a continuous function that increases monotonically from 0 to 100% between consecutive heel strikes, so it provides continuous information of the timing inside the current step and can be used to synchronize the robot’s action with the current step timing.

**FIGURE 4:**
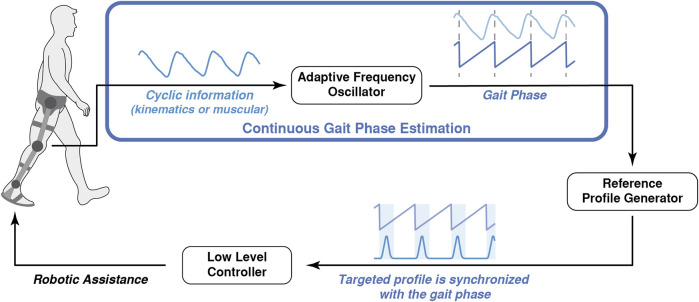
Conceptual representation of the robot-gait coordination based on the gait phase estimation performed by an Adaptive Frequency Oscillator: the AFO estimates the continuous gait phase and uses it to synchronize the application of an assistive profile.

We have identified two main methods to estimate the gait phase in real-time: the first method is based on the duration of previous steps (Awad et al., 2017b; Jin et al., 2017; Ding et al., 2018), while the second method uses adaptive frequency oscillators (also called adaptive oscillators, AOs) to learn features of the gait as a cyclic process, including the gait phase (Ronsse et al., 2011; Giovacchini et al., 2015; Seo et al., 2016). Additionally, a third residual subgroup includes those methods that use kinematic information and machine learning or optimization techniques. In Figure 4, we depict a representation of a coordination strategy based on the gait phase estimated by an AO.

#### 3.2.1 Step Time Based Gait Phase Estimation

The core of this strategy consists of estimating the phase of the gait cycle considering the duration of the last step and the time lapse from the last heel strike. Therefore, it is a quite simple strategy to estimate the continuous gait phase while the step duration remained constant. Once the gait phase is known, it is used to determine the timing of the assistive actions of the robotic exoskeleton (Oymagil et al., 2007; Bae et al., 2015, 2018a, 2018b; Ding et al., 2016a, 2016b, 2017, 2018; Lee et al., 2016, Lee et al., 2017 G.; Awad et al., 2017a, 2017b; Jin et al., 2017; Young et al., 2017; Bougrinat et al., 2019; Huo et al., 2019; Kang et al., 2019; Kim et al., 2019; Siviy et al., 2020; Haufe et al., 2021).

#### 3.2.2 Adaptive Frequency Oscillators

To synchronize the action of a robotic exoskeleton with the human gait, several authors considered the periodic nature of gait-related signals (such as kinematics or muscular activity) and used its features (e.g., amplitude, frequency, or phase) for the control of the robot. AOs are mathematical tools that can be synchronized with a quasi-periodic signal by learning its features as variable states (Ronsse et al., 2013). Although this approach initially worked for quasi-sinusoidal signals, it was extended to non-sinusoidal periodic signals by coupling a pool of AOs to a kernel-based non-linear filter (Ronsse et al., 2011). Once converged, the amplitude, frequency, and phase of the AO corresponded to the amplitude, frequency, and phase of the input signal. If this input was a characteristic gait signal, its features could be used inside the control strategy of a robotic wearable device.

The information provided by the AO could be used only for assistance timing, considering the phase and frequency of the gait (Lenzi et al., 2013; Aguirre-Ollinger, 2015; Yan et al., 2015b; Cempini et al., 2015; Giovacchini et al., 2015; Ruiz Garate et al., 2016; Seo et al., 2016; Parri et al., 2017; Ruiz Garate et al., 2017; Sanz-Morere et al., 2018; Aguirre-Ollinger et al., 2019; Ishmael et al., 2019; Aguirre-Ollinger and Yu, 2021; Talatian et al., 2021; Tricomi et al., 2021), or it could also be used to reconstruct the source signal to use it directly as a reference for the low-level controller (Zanotto et al., 2014) or as filtered information to generate an assistive reference (Ronsse et al., 2011).

Although the input of an AO was usually a kinematic signal, as reported in the mentioned articles, other signals could also be used. Measures from insole pressure sensors (Grazi et al., 2015; van Dijk et al., 2017), the linear envelope of muscular activity (Aguirre-Ollinger, 2013), or joint torques (Han et al., 2019) were also demonstrated to be reliable sources of information to estimate the gait phase with AOs.

An extended approach, called Particularly Shaped Adaptive Oscillator (PSAO), was used to determine the gait phase and frequency from the hip angle (Seo et al., 2016; Lee H.-J. et al., 2017, Lee et al., 2017 S.-H., 2019). In addition, other information such as the user’s speed and the ground inclination was also estimated from the PSAO states and one inertial measurement unit (IMU) located at the user’s back.

#### 3.2.3 Kinematics-Based Gait Phase Estimation

These methods rely on the kinematic information of the joints to estimate the continuous real-time gait phase of the user. For example, the ANdROS prototype compared the movement of both knees and hips with reference trajectories and minimized the difference between them to estimate the current gait phase (Aoyagi et al., 2007; Unluhisarcikli et al., 2011).

Other methods used machine learning techniques to learn the gait features from experimental data. In (Kang et al., 2020), the authors used a neural network model based on hip and thigh angles to estimate the gait phase in real-time. In contrast, in (Li et al., 2011; Zhang et al., 2021), the authors opted for learning a model to characterize the kinematics of the gait and then used a cross-correlation method (Li et al., 2011) or a particle swarm optimization (Zhang et al., 2021) to estimate the continuous gait phase.

### 3.3 Coordination Based on User Kinetics and Kinematics

**FIGURE 5:**
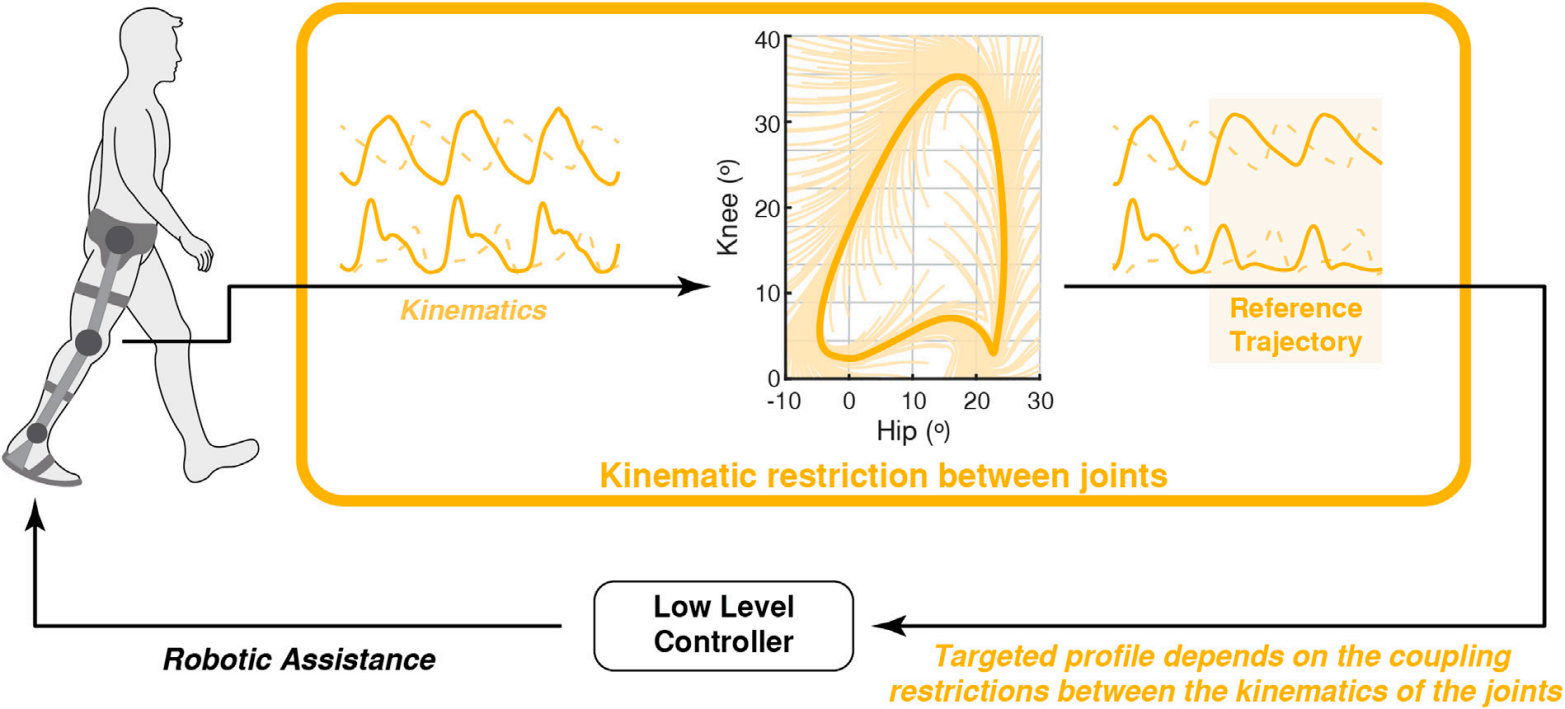
Conceptual representation of the robot-gait coordination based on the kinematics of the gait: a flow control imposed restrictions between the movement of hip and knee to generate the assistive profile that is applied by the robot.

This section presents the coordination methodologies that relied on the users’ movements. These techniques calculate the assistance according to the current movement of the limbs and can be divided according to the movements or restrictions that are considered: 1) residual movements of the assisted limb, 2) movements of the unassisted limb, or 3) coupling restriction between joint movements. Figure 5 represents an example of the working principle of these strategies.

#### 3.3.1 Coordination Based on the Movement of the Assisted Limb

Some authors calculated the assistive action of their robotic devices based on the residual movements of the leg that was aimed to be assisted. For example, in (Lai et al., 2013), the authors exploited the strong correlation between the knee angle and hip angular velocity during the swing phase of gait. Other sources of information were the pressure measurements between the thigh muscles and the exoskeleton straps (Wu et al., 2015) for generating a velocity reference profile, or the biological moment of the ankle for generating assistive (Fang and Lerner, 2021; Gasparri et al., 2019; Orekhov et al., 2020, 2021) or resistive (Conner et al., 2020) torque profiles.

#### 3.3.2 Coordination Based on the Movement of the Non-Assisted Limb

The assistance provided by a unilateral exoskeleton can be based on the assumption that the movement of both legs is delayed 180°. This paradigm is called echo-control (Wang et al., 2013) and implies that the movement of one leg can be used to estimate the movement of the contralateral leg to apply an assistive strategy or directly replicate the gait pattern of the non-assisted leg (Nguyen et al., 2013; Kawamoto et al., 2014, 2015; Zhang et al., 2016; Xie and Huang, 2019; Baser et al., 2020; Lora-Millan et al., 2020). The prototype developed by Peng et al. also based its movement on sound limb kinematics, but according to a leader-follower multi-agent system framework (Peng et al., 2020).

Instead of directly using the trajectory depicted by the unimpaired leg, some authors used regression models to estimate the desired position of the impaired limb according to the position of the unimpaired limb. The complementary limb motion estimation (CLME) strategy computed the paretic limb’s targeted joint positions based on the healthy one’s current position by using synergetic information by referencing unimpaired walking subjects (Vallery and Buss, 2006; Vallery et al., 2007, 2009).

This method was extended by Hassan et al., who included the movement of a walking aid (a cane) as an input of the PCA algorithm (Hassan et al., 2012, 2018) to estimate a target trajectory for the hip and knee joints. Nunes et al. also extended the PCA approach, but to obtain the torque primitives that originated the movement and used them as references for their knee exoskeleton (Nunes et al., 2018).

#### 3.3.3 Coordination Based on the Restriction Between the Movements of the Joints

Some authors exploited the inter-joint restrictions to coordinate the assistance provided by a robotic exoskeleton. This is the case for the force-field control that imposed a force field around a healthy foot trajectory in the joint space, so that users could be forced to move the foot inside this trajectory (Banala et al., 2007, 2009). The second version of this controller included a tangential force to the prescribed trajectory to assist the user in the movement (Winfree et al., 2011; Jin et al., 2015, 2018; Srivastava et al., 2015; Hidayah et al., 2020).

Although the path control reinforced the constraints between hips and knees in the joint space during the swing (Martinez et al., 2018), it also allowed the user to modify the step length, because the users were able to control the exoskeleton freely during the stance phase. This controller was expanded by adding a tangential force to the prescribed path to assist the movement across it. This new version was called flow control (Martinez et al., 2019). Although this controller was initially developed for bilateral exoskeletons, it was adapted to be used in a knee exoskeleton in a single-joint flow control paradigm (Martinez et al., 2020).

Considering a complete biomechanical model also leads to restrictions on the joint configuration. This is the case for the controller that considered the virtual pivot point (VPP) as the point above the center of mass through which the ground reaction forces should pass to obtain a stable gait. This controller aimed to apply such joint torques to modify the directions of these forces and ensure a stable gait by guiding them through the VPP (Zhao et al., 2017; Sharbafi et al., 2018).

### 3.4 Coordination Based on Muscular Activity

**FIGURE 6:**
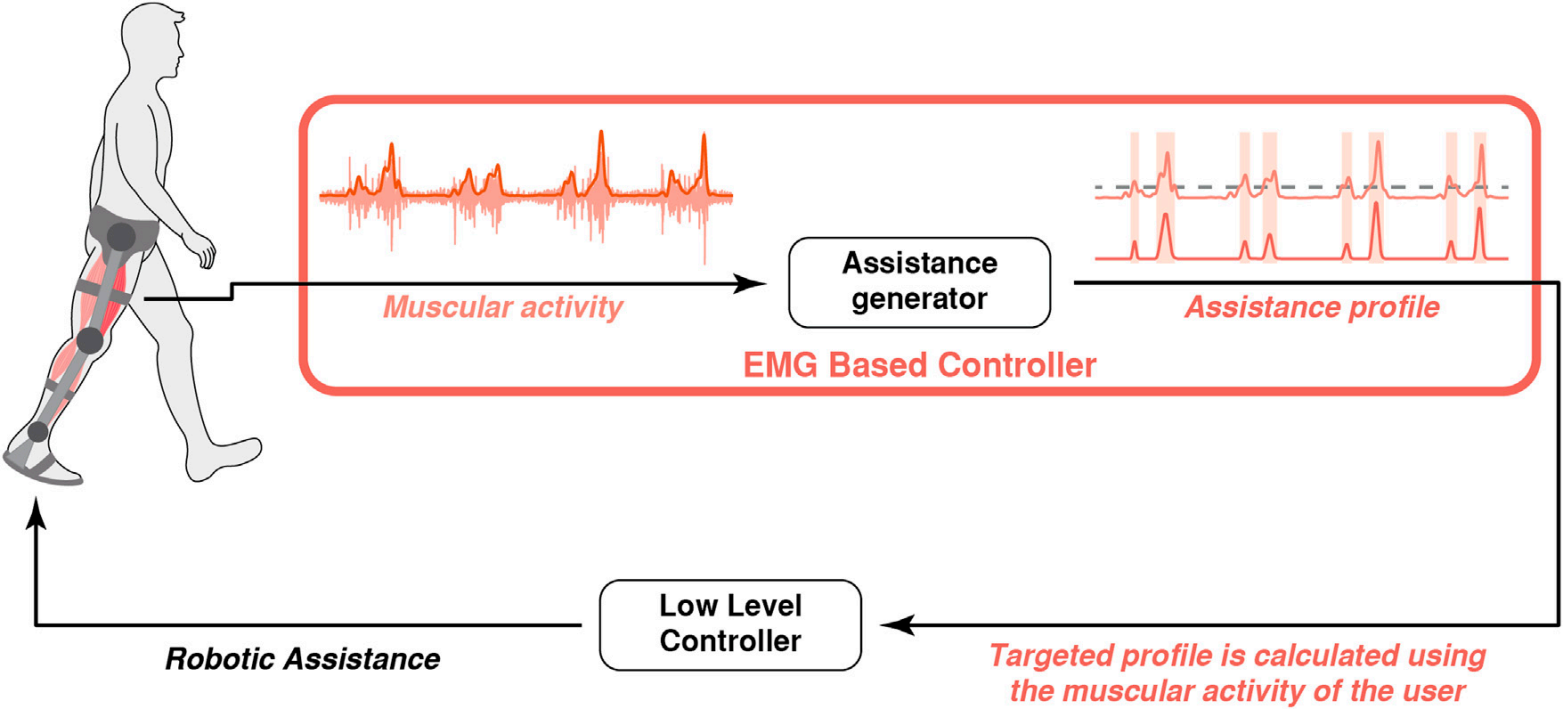
Conceptual representation of the robot-gait coordination based on the EMG signals from the user: the controller generates an assistance whose timing and amplitude depend on the muscular activity.

Limb movements are inevitably coordinated with the biosignals that recruit the muscles involved in movements. Because of this, some authors used these signals to control their robotic exoskeletons, as represented in Figure 6. Three different approaches have been found in the literature: 1) using muscular activity as a trigger to execute predefined trajectories in the exoskeleton (Kawamoto et al., 2010), 2) calculating the assistive torque proportionally to the muscular activity (the approach called myoelectric proportional control) (Gordon and Ferris, 2007; Nilsson et al., 2014) or 3) employing neuromechanical models to calculate the joint torque from the activity of the muscles involved in the gait (Fleischer and Hommel, 2008).

#### 3.4.1 Exoskeleton Actions Triggered by Muscular Activity

Similar to detecting certain gait key-events, muscular activity can also be detected and used to trigger a variety of actions in a device. Typically, this controller detects when muscular activity is above a certain threshold to perform an assistive action, such as triggering a torque profile (Kawamoto et al., 2010) or some predefined movements (Nilsson et al., 2014; Watanabe et al., 2014, 2020).

#### 3.4.2 Proportional Myoelectric Control

In contrast to the previous strategy, in which muscular activity is discretely evaluated through a comparison with a threshold, proportional myoelectric control uses information from voluntarily activated gait muscles to continuously assist limb movement. This controller generates an assistance profile that is proportional to the recruitment of these muscles (Nilsson et al., 2014; Sczesny-Kaiser et al., 2019; Tan et al., 2018; Watanabe et al., 2014, 2020). The muscles involved in this controller depends on the morphology of the robotic exoskeleton, while the above mentioned works use flexor/extensor muscles of the hip and knee to assist these joints, soleus and gastrocnemius activity can be used to command an ankle exoskeleton (Cain et al., 2007; Gordon and Ferris, 2007; Sawicki and Ferris, 2008; Kinnaird and Ferris, 2009; Kao et al., 2010; Koller et al., 2017).

Although the myoelectric proportional control usually utilize a fixed gain to calculate the assistance profile, Koller et al. introduced an adaptive gain that is a function of muscular activity (Koller et al., 2015). This adaptive paradigm leads to a more efficient assistive strategy by allowing users to find the optimal gait. An extension of the myoelectric proportional control avoids the co-contraction of pneumatic artificial muscle actuators by inhibiting flexor actuators when the extensor muscle is recruited (Sawicki and Ferris, 2009).

As an improvement of the proportional myoelectric control, the proportional myoelectric propulsion (PMP) controller adjusts not only the amplitude of the assistance but also its timing (Takahashi et al., 2015). The PMP controller generates a torque profile proportional to the muscular activation when the ground reaction force is directed anteriorly to assist the plantar flexion ankle in hemiparetic subjects. In the second version of this controller, exoskeleton assistance is also modulated by gait speed, as a higher gait speed needed higher assistance (McCain et al., 2019).

#### 3.4.3 Neuromechanical Models for Joint Torque Estimation

The muscular activation signal can also be used to estimate joint torque using neuromechanical models. For example, Fleischer et al. developed a biomechanical model to estimate knee torque based on EMG recordings from the knee flexor/extensor muscles and provided it through an exoskeleton to assist the gait (Fleischer and Hommel, 2008). In contrast, Durandau et al. exploited the synergies between muculo-tendom units, so they developed a user-specific neuromechanical model based on 12 units, although measurements of only 8 of them were used (Durandau et al., 2019). This model estimated the joint torque, and a fraction of it was provided to the users to assist the movement of their lower limbs.

### 3.5 Central Pattern Generators

**FIGURE 7:**
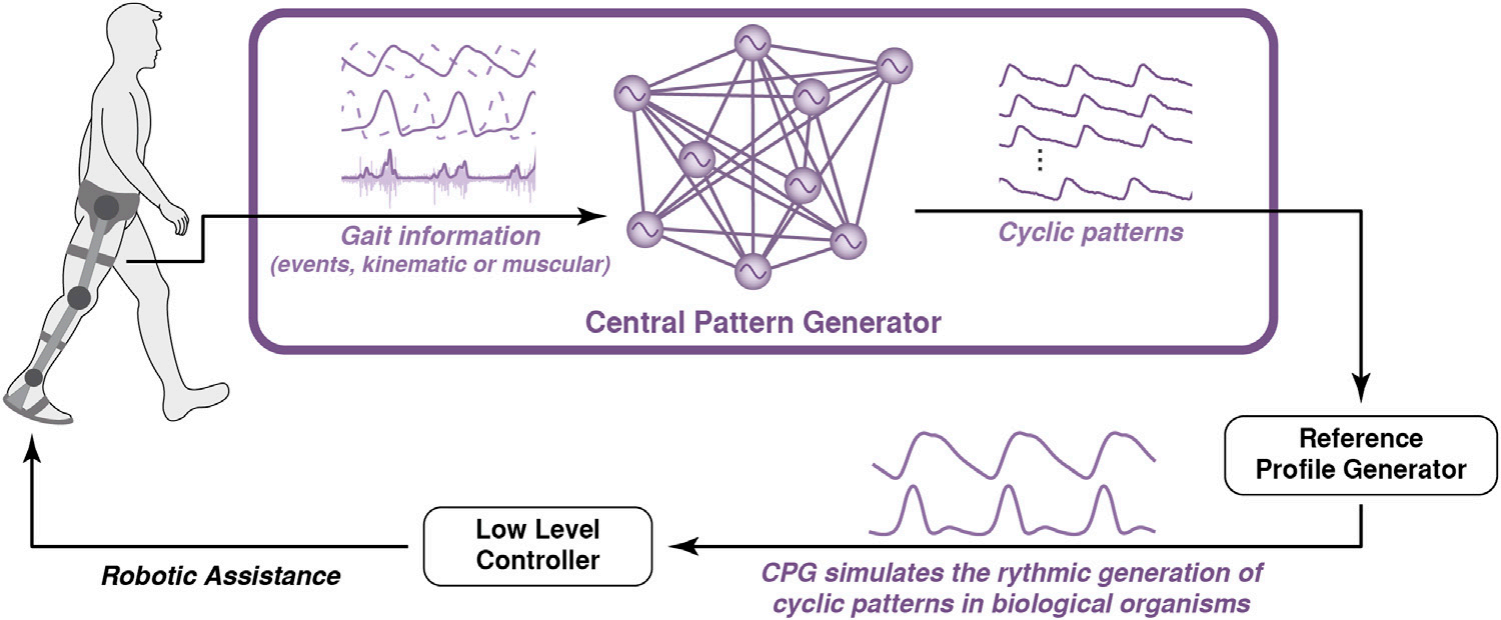
Conceptual representation of the robot-gait coordination based on the use of a CPG: it estimates cyclic signals coordinated with the user’s gait that is used to generate the assistive profile for the robot.

Following a biomimetic approach, some authors have opted to emulate the central pattern generators (CPGs) responsible for generating coordinated patterns of cyclic activity that play a crucial role in the locomotion of vertebrate and invertebrate animals (Ijspeert, 2008). By mathematically modeling these CPG neural networks, it is possible to obtain robust and rhythmic movement characteristics of bipedal locomotion (Mishra et al., 2013). If CPGs are synchronized with an external signal, typically a kinematic signal, they can also be used to coordinate the assistance provided by a robotic exoskeleton (Figure 7).

For example, Mishra et al. described an algorithm that used a CPG with the movement of the sound leg of a stroke survivor to determine the trajectory of a rehabilitation robot that would assist the movement of their paretic leg (Mishra et al., 2014). In contrast, the CPG proposed for the Curara prototype simulated the excitation and inhibition of neurons of the central nervous system. It used the interaction torque between the robot and the impaired limb to estimate the gait phase and generate an assistive reference trajectory from a predefined pattern (Tsukahara and Hashimoto, 2016; Mizukami et al., 2018). Other authors opted for synchronizing the CPG output with the detection of floor contact events (Dzeladini et al., 2016; Tamburella et al., 2020) or with the estimated knee torque from the knee flexor/extensor EMG (Gui et al., 2017).

### 3.6 Experimental Validation

Across the reviewed literature, the inconsistency found in the experimental validation of the reviewed control strategies is remarkable. As reported in other reviews (Pinto-Fernandez et al., 2020; Baud et al., 2021), the lack of common validation protocols and metrics makes it extremely difficult to compare results from different devices and reliably assess the benefits of each control strategy. Regarding the reviewed works, we found two main issues that were divergent: 1) the subject population that tested the devices and 2) the evaluated outcomes. Both items are summarized in Supplementary Table S1 for each reviewed paper.

#### 3.6.1 Subjects Involved in the Validation

The validation tests reported in the reviewed literature can be divided into three types. The first concerns the technical feasibility of the proposed device and/or the control method. In this case, some papers presented results in which no subjects were involved in the evaluation. In other cases, the authors reported simulation results (Mishra et al., 2014; Sharbafi et al., 2018) or experimental results on the robot’s ability to follow a prescribed trajectory, reject disturbances, or generate assistive profiles in time (Aoyagi et al., 2007; Unluhisarcikli et al., 2011; Zhang et al., 2016). Similarly, people with unimpaired or impaired walking were involved in other works just because their data were needed for the control strategies to use as input. In these cases, the authors only evaluated the assistance generation and how it was provided, instead of the effects on the unimpaired walking participants (Fleischer and Hommel, 2008; Hassan et al., 2012; Shamaei et al., 2013; Giovacchini et al., 2015; Zhou et al., 2016; Gui et al., 2017) or impaired walking participants involved (Sanz-Morere et al., 2018; Huo et al., 2019; Baser et al., 2020).

In contrast, other studies involved human subjects because they were focused on evaluating the real effects of the exoskeleton’s assistance. Some authors involved unimpaired walking subjects because they were the target users (Mooney et al., 2014b; Galle et al., 2017; Malcolm et al., 2018; Acosta-Sojo and Stirling, 2022), although sometimes they participated as an earlier validation before involving impaired walking subjects (Vallery et al., 2007; Winfree et al., 2011; Hassan et al., 2012; Dzeladini et al., 2016; Seo et al., 2016). Typically, after such an early validation stage, the effects of the devices were evaluated in actually impaired walking subjects (Srivastava et al., 2015; Lee H.-J. et al., 2017; Hassan et al., 2018; Tamburella et al., 2020). On other occasions, these subjects were directly involved in experimental validations to assess the effect of a robotic exoskeleton in this population without the need for previous validations (Bae et al., 2015; Lerner et al., 2017a; Tan et al., 2018).

#### 3.6.2 Assessed Outcomes

As mentioned above, some authors reported technical validation of their devices, in which they evaluated the robot’s ability to follow a prescribed trajectory, reject disturbances, or generate assistive profiles at the correct time (Fleischer and Hommel, 2008; Beyl et al., 2011; Hassan et al., 2012; Shamaei et al., 2013; Zhou et al., 2016). Most of the reviewed articles reported information about how assistance affected those subjects who wore the exoskeleton.

These effects were measured in impaired and unimpaired walking subjects while the robotic devices were assisting their gait. Throughout the reviewed literature, the authors focused on three different aspects during these validations. Most of them assessed how assistance modified the kinematics of the gait to produce a more symmetric gait (Bae et al., 2015; Arazpour et al., 2016; Liu and Wang, 2020) or a pattern closer to natural gait (Kawamoto et al., 2009; Winfree et al., 2011; Srivastava et al., 2015; Martinez et al., 2020; Gomez-Vargas et al., 2021) reduce drop-foot (Blaya and Herr, 2004; Yeung et al., 2017) or crouch gait (Lerner et al., 2017a), improve step length and height (Hidayah et al., 2020), or increase gait speed (Ruiz Garate et al., 2017; Mizukami et al., 2018).

Other authors went beyond and evaluated the physiological effects of the provided gait assistance. Several authors detected changes in the lower limbs’ muscular activity due to the robot’s action, pointing out that the subjects adapted their natural patterns according to the provided assistance (Gordon and Ferris, 2007; Steele et al., 2017; Tan et al., 2018). Similarly, some authors found reductions in the metabolic cost of transport, indicating that robotic assistance led to more efficient gait patterns with lower energetic cost (Koller et al., 2015; Lee H.-J. et al., 2017; Ding et al., 2018).

## 4 Discussion

**FIGURE 8:**
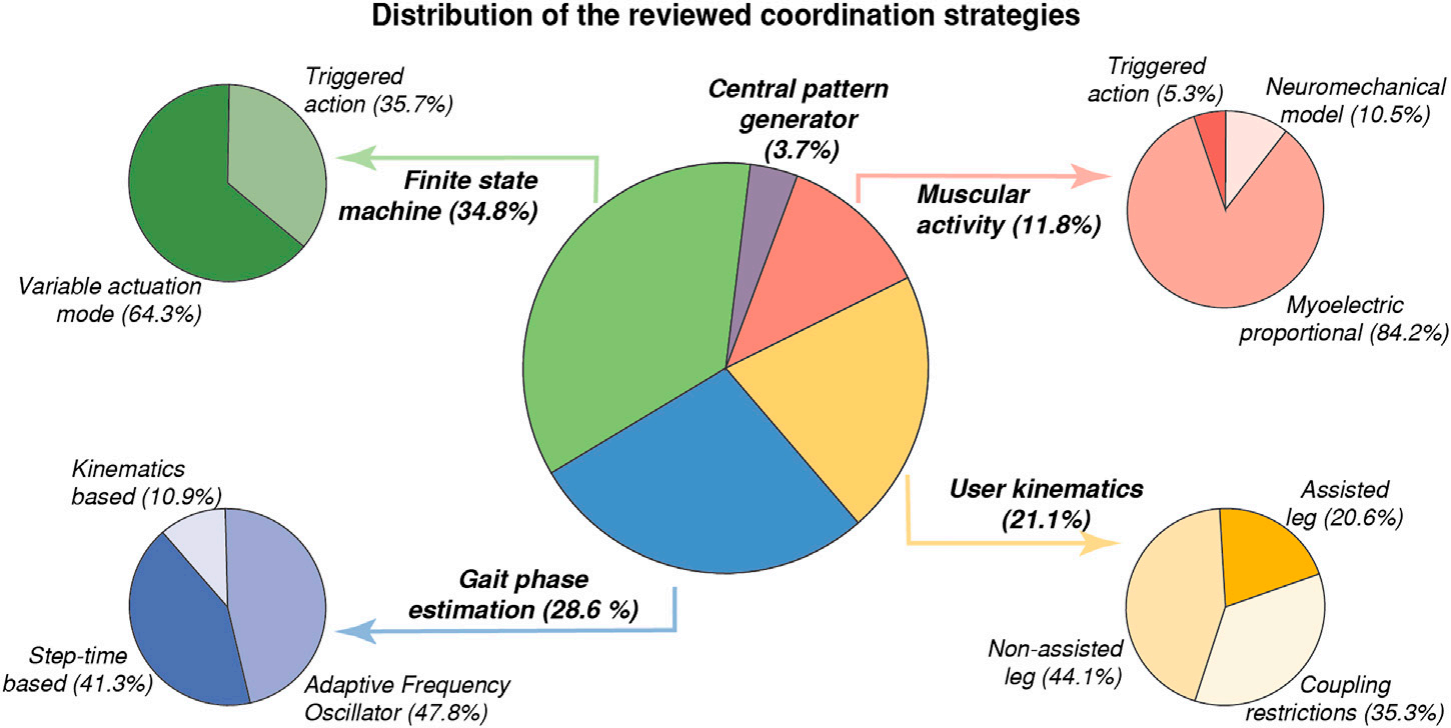
Articles distribution according to the strategy proposed to coordinate the action of a partial robotic exoskeleton with the actual user gait.

In this paper, we have reviewed and classified the main control strategies that aim to synchronize the action of partial robotic exoskeletons with the current gait of their users. The distribution of the analyzed literature is represented in Figure 8. We have grouped the articles in this review according to their coordination strategies. The FSM-based coordination strategy was the most frequent, appearing in 34.8% of the reported articles due to its simplicity and favorable results. The second most common coordination strategy was based on continuous gait phase estimation, which occurred in 28.6% of the total reviewed articles. Among all of them, the methods based on the user’s kinematics were 21.1%, while the approaches based on muscular activity or CPG were only 11.8% and 3.7%, respectively.

**FIGURE 9:**
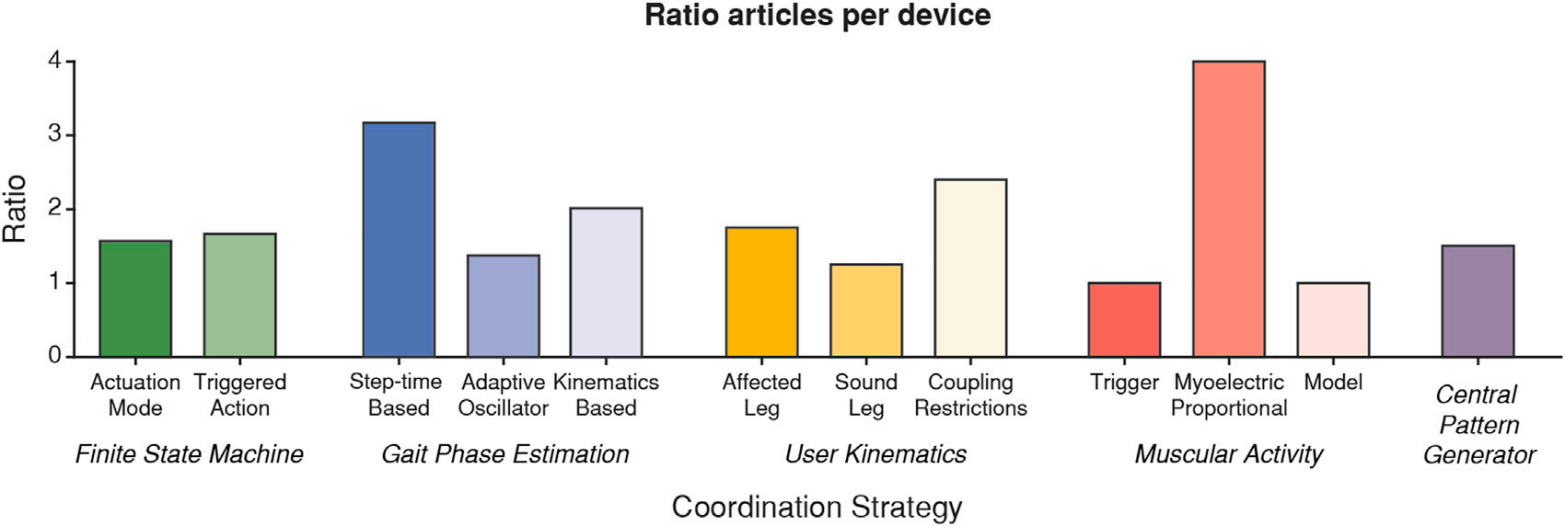
Average number of articles per device in each of the coordination strategies analyzed.

Regarding the number of devices that have been used in each coordination methodology, Figure 9 represents the ratio of articles per device that have been reported in each strategy. This ratio indicates the average number of papers that reported results with the same device, so a high ratio means that the published articles involved few different devices, and therefore, the application of the control paradigm is not as widespread as it could seem. Remarkably, the highest ratios correspond to the time-based gait phase estimation and the myoelectric proportional coordination strategies. This is because the Soft Exosuit from Harvard University (Bae et al., 2015, 2018a, 2018b; Ding et al., 2016a, 2016b, 2017, 2018; Lee et al., 2016, Lee et al., 2017 G.; Awad et al., 2017a, 2017b; Kim et al., 2019; Siviy et al., 2020) was used in 68.4% of articles that estimated the continuous gait phase using the step time, whereas the HAL prototype (Kawamoto et al., 2010; Nilsson et al., 2014; Sczesny-Kaiser et al., 2019; Tan et al., 2018, 2020; Watanabe et al., 2014, 2020) and the ankle-foot-orthosis from the University of Michigan (Cain et al., 2007; Gordon and Ferris, 2007; Sawicki and Ferris, 2008; Kinnaird and Ferris, 2009; Kao et al., 2010; Koller et al., 2015, 2017) supposed, each one, the 43.8% of articles that reported results using a myoelectric proportional controller.

**FIGURE 10:**
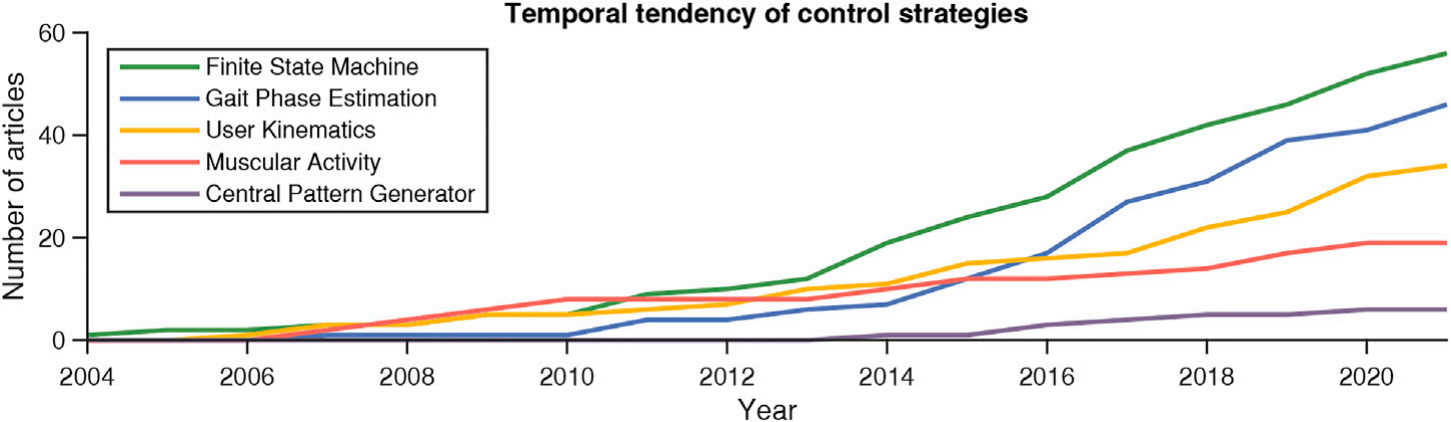
Cumulative frequency of the number of published articles related to each of the identified coordination methodologies between partial exoskeletons and human movements.

Figure 10 illustrates the cumulative frequency of articles related to each identified coordination strategy. As previously mentioned, FSM-based methods have been postulated to be the most frequent technique. However, the increasing tendency of algorithms that estimate the continuous gait phase is remarkable, surpassing muscular coordination and kinematics-based techniques, although their use started several years later. This implies the high interest aroused by these algorithms as tools for successfully coordinating the action of partial exoskeletons.

**FIGURE 11:**
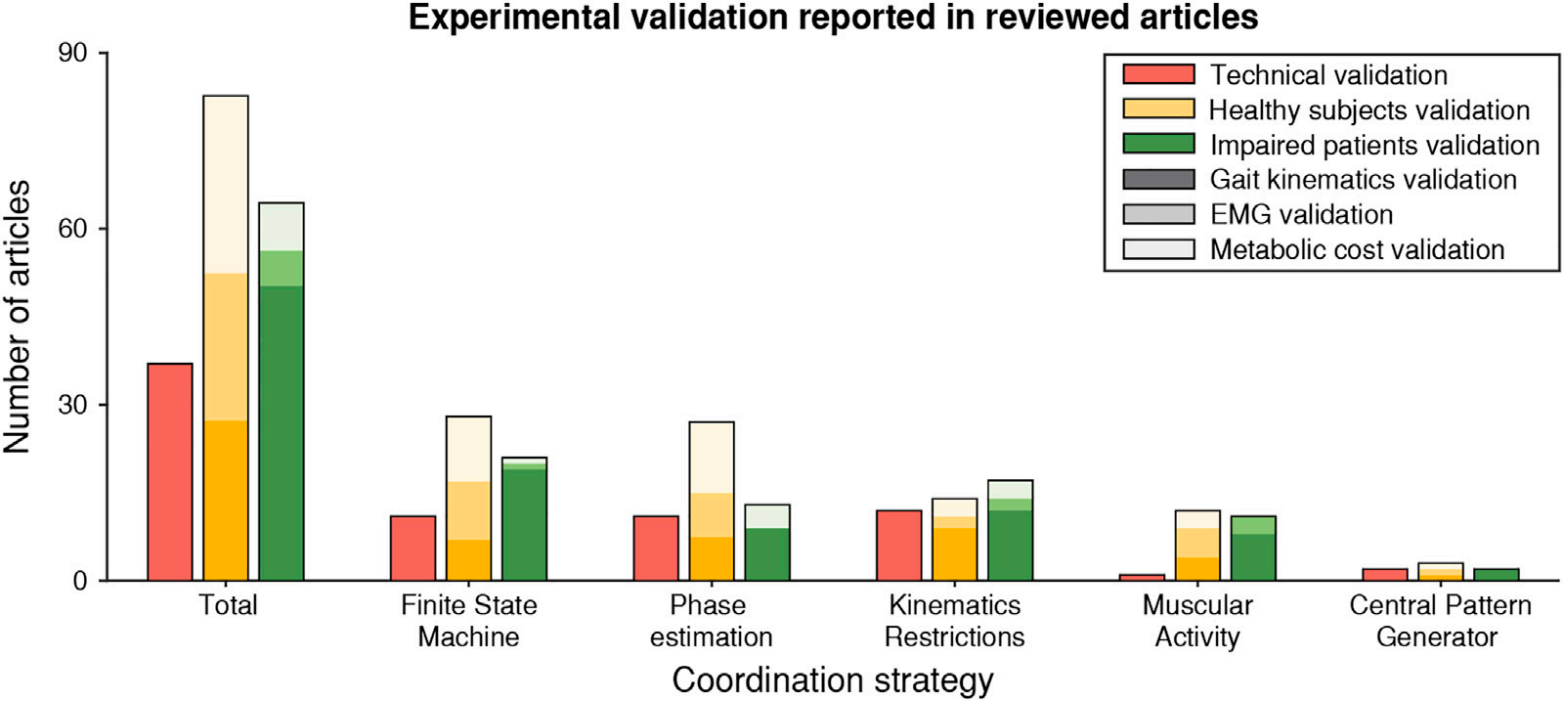
Experimental validations of the proposed coordination strategies. We represent the overall distributions and the detailed distributions for each of the five main coordination methodologies. Each color (red, amber, and green) represents a kind of validation (technical, with unimpaired walking subjects, or with impaired walking subjects, respectively). The lightness of the color indicates the outcome used during the validation: darkest, medium, and lightest correspond to gait kinematics, EMG, and metabolic cost validations, respectively.

Regarding the experimental validations that the authors proposed for their devices, we have identified three main kinds: 1) technical validations that only assessed the generation of the assistance without analyzing its effects, 2) validations where the assistance was provided to unimpaired walking subjects, and 3) validations where the assistance was provided to impaired walking subjects. An overview of the distribution of the experimental validations in the reviewed articles is depicted in Figure 11.

Regarding the technical validations, it is a common practice that some authors reported the feasibility of their devices to provide the commanded assistance. However, some of them merely present these technical results without assessing the effects on the targeted population in subsequent studies (Aoyagi et al., 2007; Fleischer and Hommel, 2008; Shorter et al., 2011; Kim et al., 2015; Wu et al., 2015; Zhang et al., 2016; Huo et al., 2019). Therefore, the validations of these exoskeletons are very limited and, although they can generate the desired assistance, they lack the assessment of the main objective of the device, i.e., assisting the gait of the user.

The main difference between experimental validation with impaired and unimpaired walking subjects is remarkable. Device validations with impaired walking subjects were mainly focused on the effects of the gait kinematics, as indicated by the high ratio (78.1%) of papers reporting such results. In contrast, the articles with unimpaired walking subject validations reported this information only in 33.3% of the articles, while focusing equally on muscular response (31%) and metabolic cost (35.7%). Compared to these ratios, the validations with impaired walking subjects reported muscular and metabolic results only in 9.4 and 12.5% of the cases, respectively.

These differences between the validation with impaired or unimpaired walking subjects show that the literature focused on different objectives for different kinds of users. While the assistance of unimpaired walking subjects was intended to reduce their muscular effort and metabolic cost during gait, the assistance of impaired walking subjects focused on the functional aspects of gait. While this is logical because the primary purpose of the assistance for these subjects is to achieve an autonomous and stable gait, it also shows a lack of understanding of how impaired walking subjects react to the assistance provided; especially whether they were able to integrate the robot action and consequently adapted their muscular activity due to the robotic assistance.

Among the validations performed on impaired walking subjects, 73.1% focused on the instantaneous effects of the applied assistance during walking. However, some authors have explored the effects of the robot’s action on the gait of the impaired walking subject after long-term rehabilitation therapies, and 26.9% of the studies that involved impaired walking subjects focused on the therapeutic benefits of using robotic devices. However, these studies focused on six different devices: anklebot (Roy et al., 2013; Forrester et al., 2016), AlterG Bionic Leg (Wong et al., 2012; Stein et al., 2014), HAL exoskeleton (Kawamoto et al., 2009; Nilsson et al., 2014; Sczesny-Kaiser et al., 2019; Tan et al., 2018; Watanabe et al., 2014, 2020), T-Flex (Gomez-Vargas et al., 2021), the first (Banala et al., 2009) and the second version (Srivastava et al., 2015) of the ALEX prototype and the Samsung’s GEMS device (Lee et al., 2019).

The promising results obtained in these studies showed that robotic therapies based on coordinated gait assistance of the impaired limb are valid approaches to rehabilitate subjects with impaired gait. However, the role of these strategies in the muscular recruitment and physiological response of users is not yet clear, although these aspects are quite relevant to the success of rehabilitation therapy. Consequently, more studies are still needed to fully validate rehabilitation therapies that could be performed using the control algorithms and devices detailed in this review.

**FIGURE 12:**
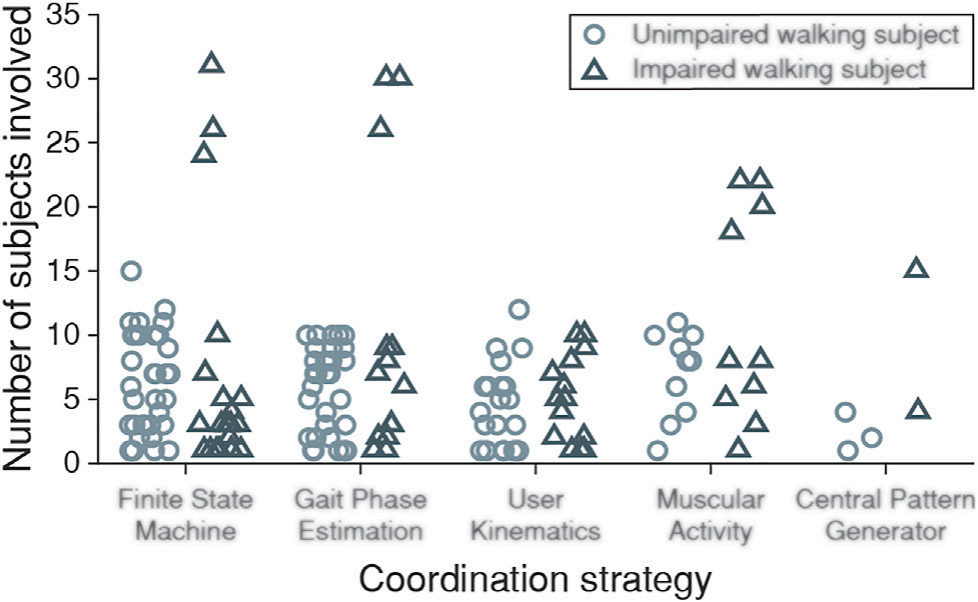
Number of subjects involved in each reported experimental validation grouped by coordination strategies. Light circular markers represent unimpaired walking subjects while dark triangular markers represent impaired walking subjects.

Another interesting aspect to consider is the number of subjects involved in the experimental validations. As shown in Figure 12, most of the reviewed papers involved a small number of subjects, limiting the level of significance of these works. The average numbers of subjects involved in the experimental validations were 5.6 for unimpaired walking subjects and 7.65 for impaired walking subjects. In this regard, only 11 of the reviewed works involved more than 15 subjects, all involving impaired walking subjects. Using a reduced sample size can be justified in studies that validate a device’s technical approach. However, rehabilitation or motor control studies should include a larger sample size to guarantee the statistical power of the conclusions.

Regarding the differences between the reported algorithms and devices, the variety is remarkable, not only in the coordination strategies, which is the scope of this document but also, in the low-level controllers and their actuation principles. There is a lack of comparative studies that establish the effects of these aspects in the assistance provided to the user. According to the reviewed literature, only a few studies have tried to compare the performance of the proposed device, usually focusing on the therapeutic outcome (Forrester et al., 2016; Lee et al., 2019; Sczesny-Kaiser et al., 2019; Stein et al., 2014; Watanabe et al., 2014, 2020), although other authors have compared the performance of two different controllers with the same device (Cain et al., 2007; Vallery et al., 2009; Koller et al., 2017; Hassan et al., 2018; Martinez et al., 2019). However, it is not yet clear which is the best method to coordinate robot assistance with the user’s gait.

As previously noted by other authors (Pinto-Fernandez et al., 2020; Baud et al., 2021), we have identified a high variability in the experimental validations that authors carried out with their devices. The absence of common protocols to evaluate the effects of robotic assistance makes it extremely difficult to extrapolate the results published by different articles for comparison. In this regard, we encourage following a common benchmark when assessing lower limb exoskeletons and their effects, as Torricelli et al. previously proposed (Torricelli et al., 2015). This would include common clinical scales to evaluate gait quality, as well as concrete assessments to evaluate robot performance, which would also be applicable to the partial exoskeletons reviewed in this document. From this perspective, previous comparisons between robotic devices that did not follow the same validation protocol should be viewed with caution as they could lead to misunderstandings.

A standardization in the assessment of robotic exoskeleton performance is required to ensure proper transition of the exoskeletons from research to clinical and domestic environments. It is necessary the definition of new “Gold-standards” to objectively assess the performance of these devices and the implications in wearer’s activities. These metrics would unify the validation process of robotic exoskeletons and would justify the scientific evidence and clinical relevance of their role in gait assistance or rehabilitation. In this regard, understanding and estimating the torque and force transmission from the robotic device to the user and the biomechanical effects of this interaction is crucial, as it could boost the therapeutic outcomes involving exoskeletons in rehabilitation therapies (Lerner et al., 2017a; George et al., 2021). These methods would enable the comparison between systems as they directly quantify the exoskeleton performance and establish a common paradigm that can be used independently of the evaluated device.

In this document, we reviewed the coordination strategies of partial robotic exoskeletons in a hardware-independent mode. Therefore, we grouped the articles only according to the coordination strategies, without considering the final implementation of the system. In fact, we considered not only single-joint or unilateral exoskeletons, but also strategies that were only related to software development (Mishra et al., 2014; Gui et al., 2017) or implemented in complete (Durandau et al., 2019; Martinez et al., 2018, 2019) or bilateral (Sawicki and Ferris, 2008; Lerner et al., 2017a, 2017b) exoskeletons whose strategies could be adapted to be used in partial robotic exoskeletons. The descriptions of the coordination strategies that we proposed were as general as possible so that other researchers could adapt them to different exoskeleton configurations independently from the original implementation. In this sense, for example, an FSM strategy can be applied regardless of the detected concrete events, or an EMG strategy can be used independently from the muscle measured and involved in the control.

Although these differences in the morphology did not affect the implementation of the coordination methods included in this review, they could affect the results obtained by the assistance. We have found multiple actuator systems and low-level controllers that include DC motors coupled to gearboxes with position (Wei et al., 2019) or impedance controllers (Villa-Parra et al., 2017) and compliant actuators under torque control, such as series elastic actuators (Giovacchini et al., 2015) or cable-driven systems (Siviy et al., 2020). This variety does not affect the implementation of the coordination strategies but still it is relevant for the interaction between the user and the device as well as the outcomes of the assistance.

These actuation and interface differences also highlight the need of common “gold-standard” metrics that globally assess the performance of these devices independently from their concrete implementational details. In this sense, other aspects, such as safety or stability, should also be objectively assessed for these wearable robotic devices, as they are key-factor to consider for a proper translation of these prototypes into real-life devices. Even the control strategies of these devices require to be assessed to ensure the robustness of future commercial robotic exoskeletons.

Independent of these implementational aspects, we can compare the coordination strategies according to each strategy’s working principle and their input and output information. As mentioned above, FSM algorithms are the most common approaches that we found. This is because they are the simplest methods from the processing and sensory points of view. Although simpler, they can adapt the action of the partial exoskeleton to discrete events, ensuring the correct coordination of both systems, human and robot, at specific instants.

In contrast to these methods, the strategies based on the estimation of the continuous gait phase lead to a continuous signal that can be used by other stages of the controller. This signal was a more versatile source of information, as it enabled its use beyond some discrete events. Regarding the methodologies for estimating this phase, the AO based approaches have a more adaptive behavior than the step-time based approaches, as they can instantaneously react to changes in gait velocity, instead of only responding to gait events. In addition, AO methodologies are simpler and require less computational effort than kinematics-based phase estimation approaches that generally rely on previous data analysis or artificial intelligence techniques.

The methods based on muscular activity require the most complex sensory systems, as they need to acquire muscular activity in real time, reject electromagnetic noise, and ensure that the movement of the robot did not interfere with the acquisition process. Additionally, neurological patients who cannot reliably recruit the involved muscles are not suitable to use this approach.

Additionally, if subjects cannot successfully generate movement in the paretic leg, they could not use those devices coordinated by the kinematics of the assisted leg or by coupling restrictions between joints. However, this disability did not interfere with the coordination strategy if it is based on the movements of the non-paretic leg, although these methodologies are more complex and usually require previous experimental data. Finally, strategies based on CPGs that simulate the generation of cyclic patterns by biological organisms are quite promising. However, the complexity of these methods and the reduced number of related papers suggest that more research is needed to completely validate their use.

Although all the reported methodologies have arisen promising results, the previously mentioned differences have implications for the safety and clinical application of these control approaches. The simpler the control algorithm, the safer implementation and wider the range of suitable users, although their performance could be slightly worse. In this sense, FSM and AO algorithms could be suitable for a greater patient typology than EMG based algorithms, although they would imply not considering the muscular activity into the therapy.

The performed review has demonstrated that this technology has reached the readiness level that ensures the proper control of partial exoskeletons. Additionally, reported muscular recruitment and metabolic cost effects are clues that point to the potential of these devices to be integrated into neural gait motor control, especially in unimpaired walking subjects. However, a research effort is still needed to validate this integration in neurologically impaired subjects.

Independent of the synchronization strategy followed, successful coordination between robot action and user’s gait will lead to the embodiment of the technology. Devices capable of matching their action with the one expected by the user will lead in integrating the exoskeleton into the body schema so it will be treated as part of the body instead of an external tool (Li et al., 2021). However, a research effort is required to demonstrate the capability of this technology of being embodied, as it has already been demonstrated for wheelchairs (Arnhoff and Mehl, 1963; Pazzaglia and Molinari, 2016) or prostheses (Penner et al., 2019; Bekrater-Bodmann, 2020).

This embodiment is the necessary next step for these robotic wearable devices. It will maximize the potential benefits of gait assistance, not only during rehabilitation therapies but also in non-supervised environments while assisting daily life activities.

## 5 Conclusion

The reviewed state of the art on coordination strategies between user gait and partial exoskeletons indicates that these techniques are mature enough to generate proper assistance for walking to both impaired and unimpaired walking subjects. More specifically, the algorithms based on FSM were the strategy most frequent, being validated repeatedly. However, the algorithms that estimate the continuous gait phase are being postulated as more robust alternatives, gaining relevance in recent years, especially those methods based on Adaptive Frequency Oscillators, due to their robustness and adaptability to gait speed changes. However, in the current state of the art, there is still a lack of studies that systematically analyze and isolate the role played by these coordination strategies, independent of the physical device on which they were implemented.

Although the theoretical objective of most robotic exoskeletons is to assist impaired walking subjects, there is a lack of information about how these subjects integrate the provided assistance in the gait neural control. Currently, the neurophysiological consequences of robotic assistance in impaired walking subjects remain elusive, including adaptation of muscle recruitment. In this context, research efforts should focus on the final states of the impaired walking users, deepening the understanding of physiological and cognitive interaction with the device, to lead to more effective neurorehabilitation and assistive approaches.

## References

[B1] Acosta-Sojo Y. Stirling L. (2022). Individuals Differ in Muscle Activation Patterns during Early Adaptation to a Powered Ankle Exoskeleton. Appl. Ergon. 98, 103593. 10.1016/j.apergo.2021.103593 34600306

[B2] Aguirre-Ollinger G. (2015). Exoskeleton Control for Lower-Extremity Assistance Based on Adaptive Frequency Oscillators: Adaptation of Muscle Activation and Movement Frequency. Proc. Inst. Mech. Eng. H. 229, 52–68. 10.1177/0954411914567213 25655955

[B3] Aguirre-Ollinger G. (2013). “Learning Muscle Activation Patterns via Nonlinear Oscillators: Application to Lower-Limb Assistance,” in 2013 IEEE/RSJ International Conference on Intelligent Robots and Systems, Tokyo, Japan, 3-7 Nov. 2013. Editor Amato N. (IEEE), 1182–1189. 10.1109/IROS.2013.6696500

[B4] Aguirre-Ollinger G. Narayan A. Yu H. (2019). Phase-Synchronized Assistive Torque Control for the Correction of Kinematic Anomalies in the Gait Cycle. IEEE Trans. Neural Syst. Rehabil. Eng. 27, 2305–2314. 10.1109/TNSRE.2019.2944665 31567098

[B5] Aguirre-Ollinger G. Yu H. (2021). Lower-Limb Exoskeleton with Variable-Structure Series Elastic Actuators: Phase-Synchronized Force Control for Gait Asymmetry Correction. IEEE Trans. Robot. 37, 763–779. 10.1109/TRO.2020.3034017

[B6] Aoyagi D. Ichinose W. E. Harkema S. J. Reinkensmeyer D. J. Bobrow J. E. (2007). A Robot and Control Algorithm that Can Synchronously Assist in Naturalistic Motion during Body-Weight-Supported Gait Training Following Neurologic Injury. IEEE Trans. Neural Syst. Rehabil. Eng. 15, 387–400. 10.1109/TNSRE.2007.903922 17894271

[B7] Arazpour M. Ahmadi F. Bahramizadeh M. Samadian M. Mousavi M. E. Bani M. A. (2016). Evaluation of Gait Symmetry in Poliomyelitis Subjects. Prosthet. Orthot. Int. 40, 689–695. 10.1177/0309364615596063 26269446

[B8] Arnhoff F. N. Mehl M. C. (1963). Body Image Deterioration in Paraplegia. J. Nerv. Ment. Dis. 137. 10.1097/00005053-196307000-00010

[B9] Awad L. N. Bae J. Kudzia P. Long A. Hendron K. Holt K. G. (2017a). Reducing Circumduction and Hip Hiking during Hemiparetic Walking through Targeted Assistance of the Paretic Limb Using a Soft Robotic Exosuit. Am. J. Phys. Med. Rehabil. 96, S157–S164. 10.1097/PHM.0000000000000800 28777105 PMC7479995

[B10] Awad L. N. Bae J. O’Donnell K. De Rossi S. M. M. Hendron K. Sloot L. H. (2017b). A Soft Robotic Exosuit Improves Walking in Patients after Stroke. Sci. Transl. Med. 9, eaai9084. 10.1126/scitranslmed.aai9084 28747517

[B11] Bacek T. Moltedo M. Serrien B. Langlois K. Vanderborght B. Lefeber D. (2022). Human Musculoskeletal and Energetic Adaptations to Unilateral Robotic Knee Gait Assistance. IEEE Trans. Biomed. Eng. 69, 1141–1150. 10.1109/TBME.2021.3114737 34559629

[B12] Bae J. Awad L. N. Long A. O'Donnell K. Hendron K. Holt K. G. (2018a). Biomechanical Mechanisms Underlying Exosuit-Induced Improvements in Walking Economy after Stroke. J. Exp. Biol. 221. 10.1242/jeb.168815 PMC5868931 29361587

[B13] Bae J. Maria De Rossi S. M. O'Donnell K. Hendron K. L. Awad L. N. Teles Dos Santos T. R. (2015). “A Soft Exosuit for Patients with Stroke: Feasibility Study with a Mobile Off-Board Actuation Unit,” in IEEE International Conference on Rehabilitation Robotics, Singapore, 11-14 Aug. 2015 (IEEE). 10.1109/ICORR.2015.7281188

[B14] Bae J. Siviy C. Rouleau M. Menard N. Odonnell K. Geliana I. (2018b). “A Lightweight and Efficient Portable Soft Exosuit for Paretic Ankle Assistance in Walking after Stroke,” in 2018 IEEE International Conference on Robotics and Automation (ICRA), Brisbane, QLD, Australia, 21-25 May 2018 (IEEE), 2820–2827. 10.1109/ICRA.2018.8461046

[B15] Banala S. K. Agrawal S. K. Scholz J. P. (2007). “Active Leg Exoskeleton (ALEX) for Gait Rehabilitation of Motor-Impaired Patients,” in 2007 IEEE 10th International Conference on Rehabilitation Robotics ICORR, Noordwijk, Netherlands, 13-15 June 2007 (IEEE), 401–407. 10.1109/ICORR.2007.4428456

[B16] Banala S. K. Kim S. H. Agrawal S. K. Scholz J. P. (2009). Robot Assisted Gait Training with Active Leg Exoskeleton (ALEX). IEEE Trans. Neural Syst. Rehabil. Eng. 17, 2–8. 10.1109/TNSRE.2008.2008280 19211317

[B17] Baser O. Kizilhan H. Kilic E. (2020). Employing Variable Impedance (Stiffness/damping) Hybrid Actuators on Lower Limb Exoskeleton Robots for Stable and Safe Walking Trajectory Tracking. J. Mech. Sci. Technol. 34, 2597–2607. 10.1007/s12206-020-0534-4

[B18] Baud R. Manzoori A. R. Ijspeert A. Bouri M. (2021). Review of Control Strategies for Lower-Limb Exoskeletons to Assist Gait. J. NeuroEngineering Rehabil. 18, 119. 10.1186/s12984-021-00906-3 PMC8314580 34315499

[B19] Bekrater-Bodmann R. (2020). Perceptual Correlates of Successful Body-Prosthesis Interaction in Lower Limb Amputees: Psychometric Characterisation and Development of the Prosthesis Embodiment Scale. Sci. Rep. 10, 1–13. 10.1038/s41598-020-70828-y 32848166 PMC7450092

[B20] Beyl P. Knaepen K. Duerinck S. Van Damme M. Vanderborght B. Meeusen R. (2011). Safe and Compliant Guidance by a Powered Knee Exoskeleton for Robot-Assisted Rehabilitation of Gait. Adv. Robot. 25, 513–535. 10.1163/016918611X558225

[B21] Blaya J. A. Herr H. (2004). Adaptive Control of a Variable-Impedance Ankle-Foot Orthosis to Assist Drop-Foot Gait. IEEE Trans. Neural Syst. Rehabil. Eng. 12, 24–31. 10.1109/TNSRE.2003.823266 15068184

[B22] Bortole M. Venkatakrishnan A. Zhu F. Moreno J. C. Francisco G. E. Pons J. L. (2015). The H2 Robotic Exoskeleton for Gait Rehabilitation after Stroke: Early Findings from a Clinical Study. J. NeuroEngineering Rehabil. 12, 54. 10.1186/s12984-015-0048-y PMC4469252 26076696

[B23] Bougrinat Y. Achiche S. Raison M. (2019). Design and Development of a Lightweight Ankle Exoskeleton for Human Walking Augmentation. Mechatronics 64, 102297. 10.1016/j.mechatronics.2019.102297

[B24] Cain S. M. Gordon K. E. Ferris D. P. (2007). Locomotor Adaptation to a Powered Ankle-Foot Orthosis Depends on Control Method. J. NeuroEngineering Rehabil. 4, 1–13. 10.1186/1743-0003-4-48 PMC2234414 18154649

[B25] Cardona M. Destarac M. Cena C. G. (2020). “Robotics for Rehabilitation: A State of the Art,” in SpringerBriefs in Applied Sciences and Technology (Singapore: Springer), 1–11. 10.1007/978-981-15-4732-4_1

[B26] Cempini M. Marconi D. Muscolo M. Moise M. Fantozzi M. Cortese M. (2015). “Relevance of Series-Elastic Actuation in Rehabilitation and Assistance Robotic: Two Cases of Study,” in 2015 IEEE 1st International Forum on Research and Technologies for Society and Industry, RTSI 2015 - Proceedings (BioRobotics Institute, Scuola Superiore sant’Anna, viale Rinaldo Piaggio, Pontedera, PI, Italy, 76–81. 10.1109/RTSI.2015.7325074

[B27] Chen B. Zi B. Qin L. Pan Q. (2020). State-of-the-art Research in Robotic Hip Exoskeletons: A General Review. J. Orthop. Transl. 20, 4–13. 10.1016/j.jot.2019.09.006 PMC6939102 31908928

[B28] Chen B. Zi B. Wang Z. Qin L. Liao W.-H. (2019). Knee Exoskeletons for Gait Rehabilitation and Human Performance Augmentation: A State-Of-The-Art. Mech. Mach. Theory 134, 499–511. 10.1016/j.mechmachtheory.2019.01.016

[B29] Chinimilli P. T. Rezayat Sorkhabadi S. M. Zhang W. (2020). Assessment of Human Dynamic Gait Stability with a Lower Extremity Assistive Device. IEEE Trans. Neural Syst. Rehabil. Eng. 28, 669–678. 10.1109/TNSRE.2020.2970207 32011260

[B30] Choi H. Park Y. J. Seo K. Lee J. Lee S.-e. Shim Y. (2018). A Multifunctional Ankle Exoskeleton for Mobility Enhancement of Gait-Impaired Individuals and Seniors. IEEE Robot. Autom. Lett. 3, 411–418. 10.1109/LRA.2017.2734239

[B31] Conner B. C. Luque J. Lerner Z. F. (2020). Adaptive Ankle Resistance from a Wearable Robotic Device to Improve Muscle Recruitment in Cerebral Palsy. Ann. Biomed. Eng. 48, 1309–1321. 10.1007/s10439-020-02454-8 31950309 PMC7096247

[B32] Di Natali C. Poliero T. Sposito M. Graf E. Bauer C. Pauli C. (2019). Design and Evaluation of a Soft Assistive Lower Limb Exoskeleton. Robotica 37, 2014–2034. 10.1017/S0263574719000067

[B33] Ding Y. Galiana I. Asbeck A. T. De Rossi S. M. M. Bae J. Santos T. R. T. (2017). Biomechanical and Physiological Evaluation of Multi-Joint Assistance with Soft Exosuits. IEEE Trans. Neural Syst. Rehabil. Eng. 25, 119–130. 10.1109/TNSRE.2016.2523250 26849868

[B34] Ding Y. Galiana I. Siviy C. Panizzolo F. A. Walsh C. (2016a). “IMU-based Iterative Control for Hip Extension Assistance with a Soft Exosuit,” in 2016 IEEE International Conference on Robotics and Automation (ICRA), Stockholm, Sweden, 16-21 May 2016 (IEEE), 3501. –3508. 10.1109/ICRA.2016.7487530

[B35] Ding Y. Kim M. Kuindersma S. Walsh C. J. (2018). Human-in-the-loop Optimization of Hip Assistance with a Soft Exosuit during Walking. Sci. Robot. 3, 1–9. 10.1126/scirobotics.aar5438 33141683

[B36] Ding Y. Panizzolo F. A. Siviy C. Malcolm P. Galiana I. Holt K. G. (2016b). Effect of Timing of Hip Extension Assistance during Loaded Walking with a Soft Exosuit. J. NeuroEngineering Rehabil. 13, 87. 10.1186/s12984-016-0196-8 PMC5048481 27716439

[B37] dos Santos W. M. Caurin G. A. P. Siqueira A. A. G. (2017). Design and Control of an Active Knee Orthosis Driven by a Rotary Series Elastic Actuator. Control Eng. Pract. 58, 307–318. 10.1016/j.conengprac.2015.09.008

[B38] Durandau G. Farina D. Asín-Prieto G. Dimbwadyo-Terrer I. Lerma-Lara S. Pons J. L. (2019). Voluntary Control of Wearable Robotic Exoskeletons by Patients with Paresis via Neuromechanical Modeling. J. NeuroEngineering Rehabil. 16, 91. 10.1186/s12984-019-0559-z PMC6637518 31315633

[B39] Dzeladini F. Wu A. R. Renjewski D. Arami A. Burdet E. van Asseldonk E. (2016). “Effects of a Neuromuscular Controller on a Powered Ankle Exoskeleton during Human Walking,” in 2016 6th IEEE International Conference on Biomedical Robotics and Biomechatronics (BioRob), Singapore, 26-29 June 2016 (IEEE), 617–622. 10.1109/BIOROB.2016.7523694

[B40] El Zahraa Wehbi F. Huo W. Amirat Y. Rafei M. E. Khalil M. Mohammed S. (2017). “Active Impedance Control of a Knee-Joint Orthosis during Swing Phase,” in 2017 International Conference on Rehabilitation Robotics (ICORR), London, UK, 17-20 July 2017 (IEEE), 435–440. 10.1109/ICORR.2017.8009286 28813858

[B41] Fahmi Bin Miskon M. Yusof M. B. A. J. (2014). “Review of Trajectory Generation of Exoskeleton Robots,” in 2014 IEEE International Symposium on Robotics and Manufacturing Automation (ROMA), Kuala Lumpur, Malaysia, 15-16 Dec. 2014, 12–17. 10.1109/ROMA.2014.7295854

[B42] Fang Y. Lerner Z. F. (2021). Feasibility of Augmenting Ankle Exoskeleton Walking Performance with Step Length Biofeedback in Individuals with Cerebral Palsy. IEEE Trans. Neural Syst. Rehabil. Eng. 29, 442–449. 10.1109/TNSRE.2021.3055796 33523814 PMC7968126

[B43] Farris R. J. Quintero H. A. Goldfarb M. (2011). Preliminary Evaluation of a Powered Lower Limb Orthosis to Aid Walking in Paraplegic Individuals. IEEE Trans. Neural Syst. Rehabil. Eng. 19, 652–659. 10.1109/TNSRE.2011.2163083 21968791 PMC3367884

[B44] Fleischer C. Hommel G. (2008). A Human--Exoskeleton Interface Utilizing Electromyography. IEEE Trans. Robot. 24, 872–882. 10.1109/TRO.2008.926860

[B45] Forrester L. W. Roy A. Hafer-Macko C. Krebs H. I. Macko R. F. (2016). Task-specific Ankle Robotics Gait Training after Stroke: a Randomized Pilot Study. J. NeuroEngineering Rehabil. 13, 51. 10.1186/s12984-016-0158-1 PMC4890526 27255156

[B46] Fox S. Aranko O. Heilala J. Vahala P. (2019). Exoskeletons. Jmtm 31, 1261–1280. 10.1108/JMTM-01-2019-0023

[B47] Galle S. Malcolm P. Collins S. H. De Clercq D. (2017). Reducing the Metabolic Cost of Walking with an Ankle Exoskeleton: Interaction between Actuation Timing and Power. J. NeuroEngineering Rehabil. 14, 1–16. 10.1186/s12984-017-0235-0 PMC5408443 28449684

[B48] Gasparri G. M. Luque J. Lerner Z. F. (2019). Proportional Joint-Moment Control for Instantaneously Adaptive Ankle Exoskeleton Assistance. IEEE Trans. Neural Syst. Rehabil. Eng. 27, 751–759. 10.1109/TNSRE.2019.2905979 30908231

[B49] George J. A. Gunnell A. J. Archangeli D. Hunt G. Ishmael M. Foreman K. B. (2021). Robust Torque Predictions from Electromyography across Multiple Levels of Active Exoskeleton Assistance Despite Non-linear Reorganization of Locomotor Output. Front. Neurorobot. 15. 10.3389/fnbot.2021.700823 PMC8595105 34803646

[B50] Giovacchini F. Vannetti F. Fantozzi M. Cempini M. Cortese M. Parri A. (2015). A Light-Weight Active Orthosis for Hip Movement Assistance. Robotics Aut. Syst. 73, 123–134. 10.1016/j.robot.2014.08.015

[B51] Gomez-Vargas D. Ballen-Moreno F. Barria P. Aguilar R. Azorín J. M. Munera M. (2021). The Actuation System of the Ankle Exoskeleton T-FLEX: First Use Experimental Validation in People with Stroke. Brain Sci. 11, 412. 10.3390/brainsci11040412 33805216 PMC8064364

[B52] Gordon K. E. Ferris D. P. (2007). Learning to Walk with a Robotic Ankle Exoskeleton. J. Biomechanics 40, 2636–2644. 10.1016/j.jbiomech.2006.12.006 17275829

[B53] Grazi L. Crea S. Parri A. Cortese M. Giovacchini F. Cempini M. (2015). “Gastrocnemius Myoelectric Control of a Robotic Hip Exoskeleton,” in 2015 37th Annual International Conference of the IEEE Engineering in Medicine and Biology Society (EMBC), Milan, Italy, 25-29 Aug. 2015 (IEEE), 3881–3884. 10.1109/EMBC.2015.7319241 26737141

[B54] Gui K. Liu H. Zhang D. (2017). A Generalized Framework to Achieve Coordinated Admittance Control for Multi-Joint Lower Limb Robotic Exoskeleton. IEEE Int. Conf. Rehabil. Robot. 2017, 228–233. 10.1109/ICORR.2017.8009251 28813823

[B55] Han Y. Zhu S. Zhou Y. Gao H. (2019). An Admittance Controller Based on Assistive Torque Estimation for a Rehabilitation Leg Exoskeleton. Intel. Serv. Robot. 12, 381–391. 10.1007/s11370-019-00289-4

[B56] Hassan M. Kadone H. Suzuki K. Sankai Y. (2012). “Exoskeleton Robot Control Based on Cane and Body Joint Synergies,” in 2012 IEEE/RSJ International Conference on Intelligent Robots and Systems, Vilamoura-Algarve, Portugal, 7-12 Oct. 2012 (IEEE), 1609–1614. 10.1109/IROS.2012.6386248

[B57] Hassan M. Kadone H. Ueno T. Hada Y. Sankai Y. Suzuki K. (2018). Feasibility of Synergy-Based Exoskeleton Robot Control in Hemiplegia. IEEE Trans. Neural Syst. Rehabil. Eng. 26, 1233–1242. 10.1109/TNSRE.2018.2832657 29877848

[B58] Haufe F. L. Kober A. M. Wolf P. Riener R. Xiloyannis M. (2021). Learning to Walk with a Wearable Robot in 880 Simple Steps: a Pilot Study on Motor Adaptation. J. NeuroEngineering Rehabil. 18, 1–14. 10.1186/s12984-021-00946-9 PMC8561899 34724940

[B59] Hidayah R. Bishop L. Jin X. Chamarthy S. Stein J. Agrawal S. K. (20201984–1993). Gait Adaptation Using a Cable-Driven Active Leg Exoskeleton (C-ALEX) with Post-Stroke Participants. IEEE Trans. Neural Syst. Rehabil. Eng. 28, 1984–1993. 10.1109/TNSRE.2020.3009317 32746320

[B60] Holanda L. J. Silva P. M. M. Amorim T. C. Lacerda M. O. Simão C. R. Morya E. (2017). Robotic Assisted Gait as a Tool for Rehabilitation of Individuals with Spinal Cord Injury: A Systematic Review. J. NeuroEngineering Rehabil. 14, 1–7. 10.1186/s12984-017-0338-7 PMC5715997 29202845

[B61] Horst R. W. (2009). “A Bio-Robotic Leg Orthosis for Rehabilitation and Mobility Enhancement,” in 2009 Annual International Conference of the IEEE Engineering in Medicine and Biology Society, Minneapolis, MN, USA, 3-6 Sept. 2009 (IEEE), 5030–5033. 10.1109/IEMBS.2009.5333581 19964374

[B62] Huo W. Arnez-Paniagua V. Ding G. Amirat Y. Mohammed S. (2019). Adaptive Proxy-Based Controller of an Active Ankle Foot Orthosis to Assist Lower Limb Movements of Paretic Patients. Robotica 37, 2147–2164. 10.1017/S0263574719000250

[B63] Ijspeert A. J. (2008). Central Pattern Generators for Locomotion Control in Animals and Robots: A Review. Neural Netw. 21, 642–653. 10.1016/j.neunet.2008.03.014 18555958

[B64] Ishmael M. K. Tran M. Lenzi T. (2019). “ExoProsthetics: Assisting Above-Knee Amputees with a Lightweight Powered Hip Exoskeleton,” in 2019 IEEE 16th International Conference on Rehabilitation Robotics (ICORR), Toronto, ON, Canada, 24-28 June 2019, 925–930. 10.1109/ICORR.2019.8779412 31374748

[B65] Jackson R. W. Collins S. H. (2015). An Experimental Comparison of the Relative Benefits of Work and Torque Assistance in Ankle Exoskeletons. J. Appl. Physiology 119, 541–557. 10.1152/japplphysiol.01133.2014 26159764

[B66] Jackson R. W. Collins S. H. (2019). Heuristic-Based Ankle Exoskeleton Control for Co-adaptive Assistance of Human Locomotion. IEEE Trans. Neural Syst. Rehabil. Eng. 27, 2059–2069. 10.1109/TNSRE.2019.2936383 31425120

[B67] Jin S. Iwamoto N. Hashimoto K. Yamamoto M. (2017). Experimental Evaluation of Energy Efficiency for a Soft Wearable Robotic Suit. IEEE Trans. Neural Syst. Rehabil. Eng. 25, 1192–1201. 10.1109/TNSRE.2016.2613886 28113402

[B68] Jin X. Cui X. Agrawal S. K. (2015). “Design of a Cable-Driven Active Leg Exoskeleton (C-ALEX) and Gait Training Experiments with Human Subjects,” in 2015 IEEE International Conference on Robotics and Automation (ICRA), Seattle, WA, USA, 26-30 May 2015, 5578–5583. 10.1109/ICRA.2015.7139979

[B69] Jin X. Prado A. Agrawal S. K. (2018). Retraining of Human Gait - Are Lightweight Cable-Driven Leg Exoskeleton Designs Effective? IEEE Trans. Neural Syst. Rehabil. Eng. 26, 847–855. 10.1109/TNSRE.2018.2815656 29641389

[B70] Kang I. Hsu H. Young A. (2019). The Effect of Hip Assistance Levels on Human Energetic Cost Using Robotic Hip Exoskeletons. IEEE Robot. Autom. Lett. 4, 430–437. 10.1109/lra.2019.2890896

[B71] Kang I. Kunapuli P. Young A. J. (2020). Real-Time Neural Network-Based Gait Phase Estimation Using a Robotic Hip Exoskeleton. IEEE Trans. Med. Robot. Bionics 2, 28–37. 10.1109/TMRB.2019.2961749

[B72] Kao P.-C. Lewis C. L. Ferris D. P. (2010). Invariant Ankle Moment Patterns when Walking with and without a Robotic Ankle Exoskeleton. J. Biomechanics 43, 203–209. 10.1016/j.jbiomech.2009.09.030 PMC2813403 19878952

[B73] Kawamoto H. Hayashi T. Sakurai T. Eguchi K. Sankai Y. (2009). “Development of Single Leg Version of HAL for Hemiplegia,” in 2009 Annual International Conference of the IEEE Engineering in Medicine and Biology Society, Minneapolis, MN, USA, 3-6 Sept. 2009, 5038–5043. 10.1109/IEMBS.2009.5333698 19964376

[B74] Kawamoto H. Kadone H. Sakurai T. Sankai Y. (2015). “Modification of Hemiplegic Compensatory Gait Pattern by Symmetry-Based Motion Controller of HAL,” in 2015 37th Annual International Conference of the IEEE Engineering in Medicine and Biology Society (EMBC), Milan, Italy, 25-29 Aug. 2015 (IEEE), 4803–4807. 10.1109/EMBC.2015.7319468 26737368

[B75] Kawamoto H. Kandone H. Sakurai T. Ariyasu R. Ueno Y. Eguchi K. (2014). “Development of an Assist Controller with Robot Suit HAL for Hemiplegic Patients Using Motion Data on the Unaffected Side,” in 2014 36th Annual International Conference of the IEEE Engineering in Medicine and Biology Society, Chicago, IL, USA, 26-30 Aug. 2014 (IEEE), 3077–3080. 10.1109/EMBC.2014.6944273 25570641

[B76] Kawamoto H. Sankai Y. (2005). Power Assist Method Based on Phase Sequence and Muscle Force Condition for HAL. Adv. Robot. 19, 717–734. 10.1163/1568553054455103

[B77] Kawamoto H. Taal S. Niniss H. Hayashi T. Kamibayashi K. Eguchi K. (2010). “Voluntary Motion Support Control of Robot Suit HAL Triggered by Bioelectrical Signal for Hemiplegia,” in 2010 Annual International Conference of the IEEE Engineering in Medicine and Biology, Buenos Aires, Argentina, 31 Aug.-4 Sept. 2010, 462–466. 10.1109/IEMBS.2010.5626191 21095652

[B78] Kim J.-H. Shim M. Ahn D. H. Son B. J. Kim S.-Y. Kim D. Y. (2015). Design of a Knee Exoskeleton Using Foot Pressure and Knee Torque Sensors. Int. J. Adv. Robotic Syst. 12, 112. 10.5772/60782

[B79] Kim J. Hwang S. Sohn R. Lee Y. Kim Y. (2011). Development of an Active Ankle Foot Orthosis to Prevent Foot Drop and Toe Drag in Hemiplegic Patients: A Preliminary Study. Appl. Bionics Biomechanics 8, 377–384. 10.3233/ABB-2011-0008

[B80] Kim J. Lee G. Heimgartner R. Arumukhom Revi D. Karavas N. Nathanson D. (2019). Reducing the Metabolic Rate of Walking and Running with a Versatile, Portable Exosuit. Science 365, 668–672. 10.1126/science.aav7536 31416958

[B81] Kim S. J. Na Y. Lee D. Y. Chang H. Kim J. (2020). Pneumatic AFO Powered by a Miniature Custom Compressor for Drop Foot Correction. IEEE Trans. Neural Syst. Rehabil. Eng. 28, 1781–1789. 10.1109/TNSRE.2020.3003860 32746300

[B82] Kinnaird C. R. Ferris D. P. (2009). Medial Gastrocnemius Myoelectric Control of a Robotic Ankle Exoskeleton. IEEE Trans. Neural Syst. Rehabil. Eng. 17, 31–37. 10.1109/TNSRE.2008.2008285 19211321 PMC2819404

[B83] Knaepen K. Beyl P. Duerinck S. Hagman F. Lefeber D. Meeusen R. (2014). Human-Robot Interaction: Kinematics and Muscle Activity inside a Powered Compliant Knee Exoskeleton. IEEE Trans. Neural Syst. Rehabil. Eng. 22, 1128–1137. 10.1109/TNSRE.2014.2324153 24846650

[B84] Koller J. R. David Remy C. Ferris D. P. (2017). “Comparing Neural Control and Mechanically Intrinsic Control of Powered Ankle Exoskeletons,” in 2017 International Conference on Rehabilitation Robotics (ICORR), London, UK, 17-20 July 2017, 294–299. 10.1109/ICORR.2017.8009262 28813834

[B85] Koller J. R. Jacobs D. A. Ferris D. P. Remy C. D. (2015). Learning to Walk with an Adaptive Gain Proportional Myoelectric Controller for a Robotic Ankle Exoskeleton. J. NeuroEngineering Rehabil. 12, 97. 10.1186/s12984-015-0086-5 PMC4634144 26536868

[B86] Lai W.-Y. Ma H. Liao W.-H. Fong D. T.-P. Chan K.-M. (2013). “HIP-KNEE Control for Gait Assistance with Powered Knee Orthosis,” in 2013 IEEE International Conference on Robotics and Biomimetics (ROBIO), Shenzhen, China, 12-14 Dec. 2013 (IEEE), 762–767. 10.1109/ROBIO.2013.6739554

[B87] Lee D. Kwak E. C. McLain B. J. Kang I. Young A. J. (2020). Effects of Assistance during Early Stance Phase Using a Robotic Knee Orthosis on Energetics, Muscle Activity, and Joint Mechanics during Incline and Decline Walking. IEEE Trans. Neural Syst. Rehabil. Eng. 28, 914–923. 10.1109/TNSRE.2020.2972323 32054583

[B88] Lee G. Ding Y. Bujanda I. G. Karavas N. Zhou Y. M. Walsh C. J. (2017a). “Improved Assistive Profile Tracking of Soft Exosuits for Walking and Jogging with Off-Board Actuation,” in 2017 IEEE/RSJ International Conference on Intelligent Robots and Systems (IROS), Vancouver, BC, Canada, 24-28 Sept. 2017 (IEEE), 1699–1706. 10.1109/IROS.2017.8205981

[B89] Lee H.-J. Lee S.-H. Seo K. Lee M. Chang W. H. Choi B.-O. (2019). Training for Walking Efficiency with a Wearable Hip-Assist Robot in Patients with Stroke. Stroke 50, 3545–3552. 10.1161/STROKEAHA.119.025950 31623545

[B90] Lee H.-J. Lee S. Chang W. H. Seo K. Shim Y. Choi B.-O. (2017b). A Wearable Hip Assist Robot Can Improve Gait Function and Cardiopulmonary Metabolic Efficiency in Elderly Adults. IEEE Trans. Neural Syst. Rehabil. Eng. 25, 1. 10.1109/TNSRE.2017.2664801 28186902

[B91] Lee S.-H. Lee H.-J. Chang W. H. Choi B.-O. Lee J. Kim J. (2017c). Gait Performance and Foot Pressure Distribution during Wearable Robot-Assisted Gait in Elderly Adults. J. NeuroEngineering Rehabil. 14, 1–10. 10.1186/s12984-017-0333-z PMC5706419 29183379

[B92] Lee S. Crea S. Malcolm P. Galiana I. Asbeck A. Walsh C. (2016). “Controlling Negative and Positive Power at the Ankle with a Soft Exosuit,” in 2016 IEEE International Conference on Robotics and Automation (ICRA), Stockholm, Sweden, 16-21 May 2016 (IEEE), 3509–3515. 10.1109/ICRA.2016.7487531

[B93] Lenzi T. Carrozza M. C. Agrawal S. K. (2013). Powered Hip Exoskeletons Can Reduce the User's Hip and Ankle Muscle Activations during Walking. IEEE Trans. Neural Syst. Rehabil. Eng. 21, 938–948. 10.1109/TNSRE.2013.2248749 23529105

[B94] Lerner Z. F. Damiano D. L. Bulea T. C. (2017a). Relationship between Assistive Torque and Knee Biomechanics during Exoskeleton Walking in Individuals with Crouch Gait. IEEE Int. Conf. Rehabil. Robot. 2017, 491–497. 10.1109/ICORR.2017.8009296 28813868 PMC10436701

[B95] Lerner Z. F. Damiano D. L. Park H.-S. Gravunder A. J. Bulea T. C. (2017b). A Robotic Exoskeleton for Treatment of Crouch Gait in Children with Cerebral Palsy: Design and Initial Application. IEEE Trans. Neural Syst. Rehabil. Eng. 25, 650–659. 10.1109/TNSRE.2016.2595501 27479974 PMC7995637

[B96] Lerner Z. F. Gasparri G. M. Bair M. O. Lawson J. L. Luque J. Harvey T. A. (2018). An Untethered Ankle Exoskeleton Improves Walking Economy in a Pilot Study of Individuals with Cerebral Palsy. IEEE Trans. Neural Syst. Rehabil. Eng. 26, 1985–1993. 10.1109/TNSRE.2018.2870756 30235140 PMC6217810

[B97] Li D. Y. Becker A. Shorter K. A. Bretl T. Hsiao-Wecksler E. T. (2011). Estimating System State during Human Walking with a Powered Ankle-Foot Orthosis. IEEE/ASME Trans. Mechatron. 16, 835–844. 10.1109/TMECH.2011.2161769

[B98] Li W.-Z. Cao G.-Z. Zhu A.-B. (2021). Review on Control Strategies for Lower Limb Rehabilitation Exoskeletons. IEEE Access 9, 123040–123060. 10.1109/ACCESS.2021.3110595

[B99] Lim B. Kyungrock Kim K. Jusuk Lee J. Junwon Jang J. Youngbo Shim Y. (2015). “An Event-Driven Control to Achieve Adaptive Walking Assist with Gait Primitives,” in 2015 IEEE/RSJ International Conference on Intelligent Robots and Systems (IROS), Hamburg, Germany, 28 Sept.-2 Oct. 2015, 5870–5875. 10.1109/IROS.2015.7354211

[B100] Liu X. Wang Q. (2020). Real-Time Locomotion Mode Recognition and Assistive Torque Control for Unilateral Knee Exoskeleton on Different Terrains. IEEE/ASME Trans. Mechatron. 25, 2722–2732. 10.1109/TMECH.2020.2990668

[B101] Lora-Millan J. S. Moreno J. C. Rocon E. (2020). “Assessment of Gait Symmetry, Torque Interaction and Muscular Response Due to the Unilateral Assistance provided by an Active Knee Orthosis in Healthy Subjects,” in 2020 8th IEEE RAS/EMBS International Conference for Biomedical Robotics and Biomechatronics (BioRob), New York, NY, USA, 29 Nov.-1 Dec. 2020 (IEEE), 229–234. 10.1109/BioRob49111.2020.9224414

[B102] Louie D. R. Eng J. J. (2016). Powered Robotic Exoskeletons in Post-stroke Rehabilitation of Gait: A Scoping Review. J. NeuroEngineering Rehabil. 13. 10.1186/s12984-016-0162-5 PMC4898381 27278136

[B103] Lovasz E.-C. Mărgineanu D. T. Ciupe V. Maniu I. Gruescu C. M. Zăbavă E. S. (2017). Design and Control Solutions for Haptic Elbow Exoskeleton Module Used in Space Telerobotics. Mech. Mach. Theory 107, 384–398. 10.1016/j.mechmachtheory.2016.08.004

[B104] Ma H. Zhong C. Chen B. Chan K.-M. Liao W.-H. (2018). User-Adaptive Assistance of Assistive Knee Braces for Gait Rehabilitation. IEEE Trans. Neural Syst. Rehabil. Eng. 26, 1994–2005. 10.1109/TNSRE.2018.2868693 30188836

[B105] Ma Y. Wu X. Yi J. Wang C. Chen C. (2019). A Review on Human-Exoskeleton Coordination towards Lower Limb Robotic Exoskeleton Systems. Int. J. Robot. Autom. 34. 10.2316/J.2019.206-0193

[B106] Malcolm P. Galle S. Van Den Berghe P. De Clercq D. (2018). Exoskeleton Assistance Symmetry Matters: Unilateral Assistance Reduces Metabolic Cost, but Relatively Less Than Bilateral Assistance. J. NeuroEngineering Rehabil. 15. 10.1186/s12984-018-0381-z PMC6085709 30092800

[B107] Martinez A. Durrough C. Goldfarb M. (2020). A Single-Joint Implementation of Flow Control: Knee Joint Walking Assistance for Individuals with Mobility Impairment. IEEE Trans. Neural Syst. Rehabil. Eng. 28, 934–942. 10.1109/TNSRE.2020.2977339 32142447

[B108] Martinez A. Lawson B. Durrough C. Goldfarb M. (2019). A Velocity-Field-Based Controller for Assisting Leg Movement during Walking with a Bilateral Hip and Knee Lower Limb Exoskeleton. IEEE Trans. Robot. 35, 307–316. 10.1109/TRO.2018.2883819

[B109] Martinez A. Lawson B. Goldfarb M. (2018). A Controller for Guiding Leg Movement during Overground Walking with a Lower Limb Exoskeleton. IEEE Trans. Robot. 34, 183–193. 10.1109/TRO.2017.2768035 28813848

[B110] McCain E. M. Dick T. J. M. Giest T. N. Nuckols R. W. Lewek M. D. Saul K. R. (2019). Mechanics and Energetics of Post-stroke Walking Aided by a Powered Ankle Exoskeleton with Speed-Adaptive Myoelectric Control. J. NeuroEngineering Rehabil. 16, 57. 10.1186/s12984-019-0523-y PMC6521500 31092269

[B111] Meng W. Liu Q. Zhou Z. Ai Q. Sheng B. Xie S. (2015). Recent Development of Mechanisms and Control Strategies for Robot-Assisted Lower Limb Rehabilitation. Mechatronics 31, 132–145. 10.1016/j.mechatronics.2015.04.005

[B112] Meuleman J. van Asseldonk E. van Oort G. Rietman H. van der Kooij H. (2016). LOPES II-Design and Evaluation of an Admittance Controlled Gait Training Robot with Shadow-Leg Approach. IEEE Trans. Neural Syst. Rehabil. Eng. 24, 352–363. 10.1109/TNSRE.2015.2511448 26731771

[B113] Mills K. Blanch P. Chapman A. R. McPoil T. G. Vicenzino B. (2010). Foot Orthoses and Gait: A Systematic Review and Meta-Analysis of Literature Pertaining to Potential Mechanisms. Br. J. Sports Med. 44, 1035–1046. 10.1136/bjsm.2009.066977 19996330

[B114] Mishra A. Ghosh R. Coscia M. Kukreja S. Chisari C. Micera S. (2014). “A Neurally Inspired Robotic Control Algorithm for Gait Rehabilitation in Hemiplegic Stroke Patients,” in 5th IEEE RAS/EMBS International Conference on Biomedical Robotics and Biomechatronics, Sao Paulo, Brazil, 12-15 Aug. 2014 (IEEE), 650–655. 10.1109/BIOROB.2014.6913852

[B115] Mishra A. Sunan H. Yu H. Thakor N. V. (2013). “Bipedal Locomotion Modeled as the Central Pattern Generator (CPG) and Regulated by Self Organizing Map for Model of Cortex,” in 2013 IEEE Point-of-Care Healthcare Technologies (PHT), Bangalore, India, 16-18 Jan. 2013 (IEEE), 50–53. 10.1109/PHT.2013.6461282

[B116] Mizukami N. Takeuchi S. Tetsuya M. Tsukahara A. Yoshida K. Matsushima A. (2018). Effect of the Synchronization-Based Control of a Wearable Robot Having a Non-exoskeletal Structure on the Hemiplegic Gait of Stroke Patients. IEEE Trans. Neural Syst. Rehabil. Eng. 26, 1011–1016. 10.1109/TNSRE.2018.2817647 29752236

[B117] Mooney L. M. Herr H. M. (2016). Biomechanical Walking Mechanisms Underlying the Metabolic Reduction Caused by an Autonomous Exoskeleton. J. NeuroEngineering Rehabil. 13, 4. 10.1186/s12984-016-0111-3 PMC4730720 26817449

[B118] Mooney L. M. Rouse E. J. Herr H. M. (2014a). Autonomous Exoskeleton Reduces Metabolic Cost of Human Walking. J. NeuroEngineering Rehabil. 11. 10.1186/1743-0003-11-151 PMC4236484 25367552

[B119] Mooney L. M. Rouse E. J. Herr H. M. (2014b). Autonomous Exoskeleton Reduces Metabolic Cost of Human Walking during Load Carriage. J. NeuroEngineering Rehabil. 11, 80. 10.1186/1743-0003-11-80 PMC4036406 24885527

[B120] Mooney L. M. Rouse E. J. Herr H. M. (2014c). “Autonomous Exoskeleton Reduces Metabolic Cost of Walking,” in 2014 36th Annual International Conference of the IEEE Engineering in Medicine and Biology Society, Chicago, IL, USA, 26-30 Aug. 2014 (IEEE), 3065–3068. 10.1109/EMBC.2014.6944270 25570638

[B121] Nilsson A. Vreede K. Häglund V. Kawamoto H. Sankai Y. Borg J. (2014). Gait Training Early after Stroke with a New Exoskeleton - the Hybrid Assistive Limb: a Study of Safety and Feasibility. J. NeuroEngineering Rehabilitation 11, 92. 10.1186/1743-0003-11-92 PMC4065313 24890413

[B122] Nunes P. F. dos Santos W. M. Siqueira A. A. G. (2018). Control Strategy Based on Kinetic Motor Primitives for Lower Limbs Exoskeletons. IFAC-PapersOnLine 51, 402–406. 10.1016/j.ifacol.2019.02.003

[B123] Orekhov G. Fang Y. Cuddeback C. F. Lerner Z. F. (2021). Usability and Performance Validation of an Ultra-lightweight and Versatile Untethered Robotic Ankle Exoskeleton. J. NeuroEngineering Rehabil. 18, 1–16. 10.1186/s12984-021-00954-9 PMC8579560 34758857

[B124] Orekhov G. Fang Y. Luque J. Lerner Z. F. (2020). Ankle Exoskeleton Assistance Can Improve Over-ground Walking Economy in Individuals with Cerebral Palsy. IEEE Trans. Neural Syst. Rehabil. Eng. 28, 461–467. 10.1109/TNSRE.2020.2965029 31940542 PMC7050636

[B125] Oymagil A. M. Hitt J. K. Sugar T. Fleeger J. (2007). “Control of a Regenerative Braking Powered Ankle Foot Orthosis,” in 2007, IEEE 10th International Conference on Rehabilitation Robotics, Noordwijk, Netherlands, 13-15 June 2007 (IEEE), 28–34. 10.1109/ICORR.2007.4428402

[B126] Parri A. Yan T. Giovacchini F. Cortese M. Muscolo M. Fantozzi M. (2017). “A Portable Active Pelvis Orthosis for Ambulatory Movement Assistance,” in Proceedings of the 2nd International Symposium on Wearable Robotics, WeRob2016, Segovia, Spain, October 18-21, 2016, 75–80. 10.1007/978-3-319-46532-6_13

[B127] Pazzaglia M. Molinari M. (2016). The Embodiment of Assistive Devices-From Wheelchair to Exoskeleton. Phys. Life Rev. 16, 163–175. 10.1016/j.plrev.2015.11.006 26708357

[B128] Peng Z. Luo R. Huang R. Yu T. Hu J. Shi K. (2020). Data-Driven Optimal Assistance Control of a Lower Limb Exoskeleton for Hemiplegic Patients. Front. Neurorobot. 14. 10.3389/fnbot.2020.00037 PMC7347968 32719595

[B129] Penner D. Abrams A. M. H. Overath P. Vogt J. Beckerle P. (2019). Robotic Leg Illusion: System Design and Human-In-The-Loop Evaluation. IEEE Trans. Human-Mach. Syst. 49, 372–380. 10.1109/THMS.2019.2896447

[B130] Pinto-Fernandez D. Torricelli D. Sanchez-Villamanan M. D. C. Aller F. Mombaur K. Conti R. (2020). Performance Evaluation of Lower Limb Exoskeletons: A Systematic Review. IEEE Trans. Neural Syst. Rehabil. Eng. 28, 1573–1583. 10.1109/TNSRE.2020.2989481 32634096

[B131] Pons J. L. (2008). “Wearable Robots,” in Wearable Robots: Biomechatronic Exoskeletons. Editor Pons J. L. (Chichester, UK: John Wiley & Sons). 10.1002/9780470987667

[B132] Popovic D. B. Tomovic R. Stein R. B. (1991). Finite State Models for Gait with Hybrid Assistive Systems. Proc. Annu. Int. Conf. IEEE Eng. Med. Biol. Soc. 13, 928–930. 10.1109/IEMBS.1991.684266

[B133] Popovic D. Stein R. B. Tomovic R. (1995). Nonanalytical Methods for Motor Control. World Scientific.

[B134] Ronsse R. De Rossi S. M. M. Vitiello N. Lenzi T. Carrozza M. C. Ijspeert A. J. (2013). Real-Time Estimate of Velocity and Acceleration of Quasi-Periodic Signals Using Adaptive Oscillators. IEEE Trans. Robot. 29, 783–791. 10.1109/TRO.2013.2240173

[B135] Ronsse R. Lenzi T. Vitiello N. Koopman B. van Asseldonk E. De Rossi S. M. M. (2011). Oscillator-based Assistance of Cyclical Movements: Model-Based and Model-free Approaches. Med. Biol. Eng. Comput. 49, 1173–1185. 10.1007/s11517-011-0816-1 21881902

[B136] Roy A. Krebs H. I. Barton J. E. Macko R. F. Forrester L. W. (2013). “Anklebot-assisted Locomotor Training after Stroke: A Novel Deficit-Adjusted Control Approach,” in 2013 IEEE International Conference on Robotics and Automation, Karlsruhe, Germany, 6-10 May 2013 (IEEE), 2175–2182. 10.1109/ICRA.2013.6630869

[B137] Ruiz Garate V. Parri A. Yan T. Munih M. Molino Lova R. Vitiello N. (2017). Experimental Validation of Motor Primitive-Based Control for Leg Exoskeletons during Continuous Multi-Locomotion Tasks. Front. Neurorobot. 11. 10.3389/fnbot.2017.00015 PMC5355439 28367121

[B138] Ruiz Garate V. Parri A. Yan T. Munih M. Molino Lova R. Vitiello N. (2016). Walking Assistance Using Artificial Primitives: A Novel Bioinspired Framework Using Motor Primitives for Locomotion Assistance through a Wearable Cooperative Exoskeleton. IEEE Robot. Autom. Mag. 23, 83–95. 10.1109/MRA.2015.2510778

[B139] Sanchez-Villamañan M. D. C. Gonzalez-Vargas J. Torricelli D. Moreno J. C. Pons J. L. (2019). Compliant Lower Limb Exoskeletons: a Comprehensive Review on Mechanical Design Principles. J. NeuroEngineering Rehabil. 16, 55. 10.1186/s12984-019-0517-9 PMC6506961 31072370

[B140] Sanz-Morere C. B. Fantozzi M. Parri A. Giovacchini F. Baldoni A. Crea S. (2018). “A Bioinspired Control Strategy for the CYBERLEGs Knee-Ankle-Foot Orthosis: Feasibility Study with Lower-Limb Amputees,” in 2018 7th IEEE International Conference on Biomedical Robotics and Biomechatronics (Biorob), Enschede, Netherlands, 26-29 Aug. 2018 (IEEE), 503–508. 10.1109/BIOROB.2018.8487692

[B141] Sawicki G. S. Ferris D. P. (2009). A Pneumatically Powered Knee-Ankle-Foot Orthosis (KAFO) with Myoelectric Activation and Inhibition. J. NeuroEngineering Rehabil. 6, 1–16. 10.1186/1743-0003-6-23 PMC2717982 19549338

[B142] Sawicki G. S. Ferris D. P. (2008). Mechanics and Energetics of Level Walking with Powered Ankle Exoskeletons. J. Exp. Biol. 211, 1402–1413. 10.1242/jeb.009241 18424674

[B143] Sczesny-Kaiser M. Trost R. Aach M. Schildhauer T. A. Schwenkreis P. Tegenthoff M. (2019). A Randomized and Controlled Crossover Study Investigating the Improvement of Walking and Posture Functions in Chronic Stroke Patients Using HAL Exoskeleton - the HALESTRO Study (HAL-Exoskeleton STROke Study). Front. Neurosci. 13, 1–13. 10.3389/fnins.2019.00259 30983953 PMC6450263

[B144] Seo K. Lee J. Lee Y. Ha T. Shim Y. (2016). “Fully Autonomous Hip Exoskeleton Saves Metabolic Cost of Walking,” in 2016 IEEE International Conference on Robotics and Automation (ICRA), Stockholm, Sweden, 16-21 May 2016, 4628–4635. 10.1109/ICRA.2016.7487663

[B145] Shamaei K. Cenciarini M. Adams A. A. Gregorczyk K. N. Schiffman J. M. Dollar A. M. (2015). Biomechanical Effects of Stiffness in Parallel with the Knee Joint during Walking. IEEE Trans. Biomed. Eng. 62, 2389–2401. 10.1109/TBME.2015.2428636 25955513

[B146] Shamaei K. Cenciarini M. Adams A. A. Gregorczyk K. N. Schiffman J. M. Dollar A. M. (2014a). Design and Evaluation of a Quasi-Passive Knee Exoskeleton for Investigation of Motor Adaptation in Lower Extremity Joints. IEEE Trans. Biomed. Eng. 61, 1809–1821. 10.1109/TBME.2014.2307698 24845291

[B147] Shamaei K. Napolitano P. C. Dollar A. M. (2013). A Quasi-Passive Compliant Stance Control Knee-Ankle-Foot Orthosis. IEEE Int. Conf. Rehabil. Robot. 2013, 6650471. 10.1109/ICORR.2013.6650471 24187288

[B148] Shamaei K. Napolitano P. C. Dollar A. M. (2014b). Design and Functional Evaluation of a Quasi-Passive Compliant Stance Control Knee-Ankle-Foot Orthosis. IEEE Trans. Neural Syst. Rehabil. Eng. 22, 258–268. 10.1109/TNSRE.2014.2305664 24608684

[B149] Sharbafi M. A. Barazesh H. Iranikhah M. Seyfarth A. (2018). Leg Force Control through Biarticular Muscles for Human Walking Assistance. Front. Neurorobot. 12, 1–13. 10.3389/fnbot.2018.00039 30050426 PMC6050398

[B150] Shi B. Chen X. Yue Z. Yin S. Weng Q. Zhang X. (2019). Wearable Ankle Robots in Post-stroke Rehabilitation of Gait: A Systematic Review. Front. Neurorobot. 13. 10.3389/fnbot.2019.00063 PMC6700322 31456681

[B151] Shorter K. A. Kogler G. F. Loth E. Durfee W. K. Hsiao-Wecksler E. T. (2011). A Portable Powered Ankle-Foot Orthosis for Rehabilitation. Jrrd 48, 459. 10.1682/JRRD.2010.04.0054 21674394

[B152] Siviy C. Bae J. Baker L. Porciuncula F. Baker T. Ellis T. D. (2020). Offline Assistance Optimization of a Soft Exosuit for Augmenting Ankle Power of Stroke Survivors during Walking. IEEE Robot. Autom. Lett. 5, 828–835. 10.1109/LRA.2020.2965072 33748413 PMC7971105

[B153] Sridar S. Qiao Z. Muthukrishnan N. Zhang W. Polygerinos P. (2018). A Soft-Inflatable Exosuit for Knee Rehabilitation: Assisting Swing Phase during Walking. Front. Robot. AI 5, 1–9. 10.3389/frobt.2018.00044 33500930 PMC7805964

[B154] Sridar S. Qiao Z. Rascon A. Biemond A. Beltran A. Maruyama T. (2020). Evaluating Immediate Benefits of Assisting Knee Extension with a Soft Inflatable Exosuit. IEEE Trans. Med. Robot. Bionics 2, 216–225. 10.1109/TMRB.2020.2988305

[B155] Srivastava S. Kao P.-C. Kim S. H. Stegall P. Zanotto D. Higginson J. S. (2015). Assist-as-Needed Robot-Aided Gait Training Improves Walking Function in Individuals Following Stroke. IEEE Trans. Neural Syst. Rehabil. Eng. 23, 956–963. 10.1109/TNSRE.2014.2360822 25314703 PMC6050016

[B156] Steele K. M. Jackson R. W. Shuman B. R. Collins S. H. (2017). Muscle Recruitment and Coordination with an Ankle Exoskeleton. J. Biomechanics 59, 50–58. 10.1016/j.jbiomech.2017.05.010 PMC5644499 28623037

[B157] Stein J. Bishop L. Stein D. J. Wong C. K. (2014). Gait Training with a Robotic Leg Brace after Stroke. Am. J. Phys. Med. Rehabil. 93, 987–994. 10.1097/PHM.0000000000000119 24901757

[B158] Takahashi K. Z. Lewek M. D. Sawicki G. S. (2015). A Neuromechanics-Based Powered Ankle Exoskeleton to Assist Walking Post-stroke: A Feasibility Study. J. NeuroEngineering Rehabilitation 12, 23. 10.1186/s12984-015-0015-7 PMC4367918 25889283

[B159] Talatian H. Karami M. Moradi H. Vossoughi G. (2021). “Design and Implementation of an Intelligent Control System for a Lower-Limb Exoskeleton to Reduce Human Energy Consumption,” in 2021 10th International Conference on Modern Circuits and Systems Technologies, MOCAST, Thessaloniki, Greece, 5-7 July 2021 (IEEE), 1–4. 10.1109/MOCAST52088.2021.9493401

[B160] Tamburella F. Tagliamonte N. L. Pisotta I. Masciullo M. Arquilla M. Van Asseldonk E. H. F. (2020). Neuromuscular Controller Embedded in a Powered Ankle Exoskeleton: Effects on Gait, Clinical Features and Subjective Perspective of Incomplete Spinal Cord Injured Subjects. IEEE Trans. Neural Syst. Rehabil. Eng. 28, 1157–1167. 10.1109/TNSRE.2020.2984790 32248116

[B161] Tan C. K. Kadone H. Watanabe H. Marushima A. Hada Y. Yamazaki M. (2020). Differences in Muscle Synergy Symmetry between Subacute Post-stroke Patients with Bioelectrically-Controlled Exoskeleton Gait Training and Conventional Gait Training. Front. Bioeng. Biotechnol. 8. 10.3389/fbioe.2020.00770 PMC7403486 32850696

[B162] Tan C. K. Kadone H. Watanabe H. Marushima A. Yamazaki M. Sankai Y. (2018). Lateral Symmetry of Synergies in Lower Limb Muscles of Acute Post-stroke Patients after Robotic Intervention. Front. Neurosci. 12, 1–13. 10.3389/fnins.2018.00276 29922121 PMC5996914

[B163] Tomovic R. McGhee R. B. (1966). A Finite State Approach to the Synthesis of Bioengineering Control Systems. IEEE Trans. Hum. Factors Electron HFE-7, 65–69. 10.1109/THFE.1966.232325

[B164] Torricelli D. Vargas J. G. Veneman J. F. Mombaur K. Tsagarakis N. Del-Ama A. J. (2015). Benchmarking Bipedal Locomotion. IEEE Robot. Autom. Mag. 22, 103–115. 10.1109/MRA.2015.244827

[B165] Tricomi E. Lotti N. Missiroli F. Zhang X. Xiloyannis M. Muller T. (2022). Underactuated Soft Hip Exosuit Based on Adaptive Oscillators to Assist Human Locomotion. IEEE Robot. Autom. Lett. 7, 936–943. 10.1109/lra.2021.3136240

[B166] Nguyen T. Komeda T. Miyoshi T. Ota L. (2013). “The Powered Gait Training System Using Feedback from Own Walking Information,” in 2013 ISSNIP Biosignals and Biorobotics Conference: Biosignals and Robotics for Better and Safer Living (BRC), Rio de Janeiro, Brazil, 18-20 Feb. 2013 (IEEE), 1–5. 10.1109/BRC.2013.6487529

[B167] Tsukahara A. Hashimoto M. (2016). “Pilot Study of Single-Legged Walking Support Using Wearable Robot Based on Synchronization Control for Stroke Patients,” in 2016 IEEE International Conference on Robotics and Biomimetics, ROBIO, Qingdao, China, 3-7 Dec. 2016, 886–891. 10.1109/ROBIO.2016.7866436

[B168] Tucker M. R. Olivier J. Pagel A. Bleuler H. Bouri M. Lambercy O. (2015). Control Strategies for Active Lower Extremity Prosthetics and Orthotics: a Review. J. NeuroEngineering Rehabilitation 12, 1. 10.1186/1743-0003-12-1 PMC4326520 25557982

[B169] Unluhisarcikli O. Pietrusinski M. Weinberg B. Bonato P. Mavroidis C. (2011). “Design and Control of a Robotic Lower Extremity Exoskeleton for Gait Rehabilitation,” in 2011 IEEE International Conference on Intelligent Robots and Systems, San Francisco, CA, USA, 25-30 Sept. 2011, 4893–4898. 10.1109/IROS.2011.6094973

[B170] Vallery H. Buss M. (2006). “Complementary Limb Motion Estimation Based on Interjoint Coordination Using Principal Components Analysis,” in 2006 IEEE Conference on Computer Aided Control System Design, 2006 IEEE International Conference on Control Applications, 2006 IEEE International Symposium on Intelligent Control, Munich, Germany, 4-6 Oct. 2006, 933–938. 10.1109/CACSD-CCA-ISIC.2006.4776770

[B171] Vallery H. Ekkelenkamp R. Buss M. van der Kooij H. (2007). “Complementary Limb Motion Estimation Based on Interjoint Coordination: Experimental Evaluation,” in 2007 IEEE 10th International Conference on Rehabilitation Robotics, ICORR’07, Noordwijk, Netherlands, 13-15 June 2007 (IEEE), 798–803. 10.1109/ICORR.2007.4428516

[B172] Vallery H. Van Asseldonk E. H. F. Buss M. Van Der Kooij H. (2009). Reference Trajectory Generation for Rehabilitation Robots: Complementary Limb Motion Estimation. IEEE Trans. Neural Syst. Rehabil. Eng. 17, 23–30. 10.1109/TNSRE.2008.2008278 19211320

[B173] van Dijk W. Meijneke C. van der Kooij H. (2017). Evaluation of the Achilles Ankle Exoskeleton. IEEE Trans. Neural Syst. Rehabil. Eng. 25, 151–160. 10.1109/TNSRE.2016.2527780 26886997

[B174] Veneman J. F. Kruidhof R. Hekman E. E. G. Ekkelenkamp R. Van Asseldonk E. H. F. van der Kooij H. (2007). Design and Evaluation of the LOPES Exoskeleton Robot for Interactive Gait Rehabilitation. IEEE Trans. Neural Syst. Rehabil. Eng. 15, 379–386. 10.1109/tnsre.2007.903919 17894270

[B175] Villa-Parra A. Delisle-Rodriguez D. Souza Lima J. Frizera-Neto A. Bastos T. (2017). Knee Impedance Modulation to Control an Active Orthosis Using Insole Sensors. Sensors 17, 2751. 10.3390/s17122751 PMC5750722 29182569

[B176] Wang W. J. Li J. Li W. D. Sun L. N. (2013). An Echo-Based Gait Phase Determination Method of Lower Limb Prosthesis. Amr 706-708, 629–634. 10.4028/www.scientific.net/amr.706-708.629

[B177] Ward J. Sugar T. Boehler A. Standeven J. Engsberg J. R. (2011). Stroke Survivors' Gait Adaptations to a Powered Ankle-Foot Orthosis. Adv. Robot. 25, 1879–1901. 10.1163/016918611X588907 25339789 PMC4203663

[B178] Watanabe H. Marushima A. Kadone H. Ueno T. Shimizu Y. Kubota S. (2020). Effects of Gait Treatment with a Single-Leg Hybrid Assistive Limb System after Acute Stroke: A Non-randomized Clinical Trial. Front. Neurosci. 13. 10.3389/fnins.2019.01389 PMC6987474 32038125

[B179] Watanabe H. Tanaka N. Inuta T. Saitou H. Yanagi H. (2014). Locomotion Improvement Using a Hybrid Assistive Limb in Recovery Phase Stroke Patients: a Randomized Controlled Pilot Study. Archives Phys. Med. Rehabilitation 95, 2006–2012. 10.1016/j.apmr.2014.07.002 25010538

[B180] Wei D. Li Z. Wei Q. Su H. Song B. He W. (2021). Human-in-the-Loop Control Strategy of Unilateral Exoskeleton Robots for Gait Rehabilitation. IEEE Trans. Cogn. Dev. Syst. 13, 57–66. 10.1109/TCDS.2019.2954289

[B181] Winfree K. N. Stegall P. Agrawal S. K. (2011). Design of a Minimally Constraining, Passively Supported Gait Training Exoskeleton: ALEX II. IEEE Int. Conf. Rehabil. Robot. 2011, 5975499. 10.1109/ICORR.2011.5975499 22275695

[B182] Witte K. A. Zhang J. Jackson R. W. Collins S. H. (2015). “Design of Two Lightweight, High-Bandwidth Torque-Controlled Ankle Exoskeletons,” in Proceedings - IEEE International Conference on Robotics and Automation, Seattle, WA, USA, 26-30 May 2015, 1223–1228. 10.1109/ICRA.2015.7139347

[B183] Wong C. K. Bishop L. Stein J. (2012). A Wearable Robotic Knee Orthosis for Gait Training. Prosthetics Orthot. Int. 36, 113–120. 10.1177/0309364611428235 22082495

[B184] Wu Q. Wang X. Du F. Zhang X. (2015). Design and Control of a Powered Hip Exoskeleton for Walking Assistance. Int. J. Adv. Robotic Syst. 12, 18. 10.5772/59757

[B185] Xia H. Kwon J. Pathak P. Ahn J. Shull P. B. Park Y.-L. (2020). “Design of A Multi-Functional Soft Ankle Exoskeleton for Foot-Drop Prevention, Propulsion Assistance, and Inversion/Eversion Stabilization,” in 2020 8th IEEE RAS/EMBS International Conference for Biomedical Robotics and Biomechatronics (BioRob), New York, NY, USA, 29 Nov.-1 Dec. 2020 (IEEE), 118–123. 10.1109/BioRob49111.2020.9224420

[B186] Xie L. Huang L. (2019). Wirerope-driven Exoskeleton to Assist Lower-Limb Rehabilitation of Hemiplegic Patients by Using Motion Capture. Aa 40, 48–54. 10.1108/AA-11-2018-0221

[B187] Xu D. Liu X. Wang Q. (2019). Knee Exoskeleton Assistive Torque Control Based on Real-Time Gait Event Detection. IEEE Trans. Med. Robot. Bionics 1, 158–168. 10.1109/TMRB.2019.2930352

[B188] Yan T. Cempini M. Oddo C. M. Vitiello N. (2015a). Review of Assistive Strategies in Powered Lower-Limb Orthoses and Exoskeletons. Robotics Aut. Syst. 64, 120–136. 10.1016/j.robot.2014.09.032

[B189] Yan T. Parri A. Fantozzi M. Cortese M. Muscolo M. Cempini M. (2015b). “A Novel Adaptive Oscillators-Based Control for a Powered Multi-Joint Lower-Limb Orthosis,” in IEEE International Conference on Rehabilitation Robotics, Singapore, 11-14 Aug. 2015. 10.1109/ICORR.2015.7281230

[B190] Yeung L.-F. Lau C. C. Y. Lai C. W. K. Soo Y. O. Y. Chan M.-L. Tong R. K. Y. (2021). Effects of Wearable Ankle Robotics for Stair and Over-ground Training on Sub-acute Stroke: a Randomized Controlled Trial. J. NeuroEngineering Rehabil. 18, 19. 10.1186/s12984-021-00814-6 PMC7847008 33514393

[B191] Yeung L.-F. Ockenfeld C. Pang M.-K. Wai H.-W. Soo O.-Y. Li S.-W. (2017). “Design of an Exoskeleton Ankle Robot for Robot-Assisted Gait Training of Stroke Patients,” in 2017 International Conference on Rehabilitation Robotics (ICORR), London, UK, 17-20 July 2017, 211–215. 10.1109/ICORR.2017.8009248 28813820

[B192] Young A. J. Foss J. Gannon H. Ferris D. P. (2017). Influence of Power Delivery Timing on the Energetics and Biomechanics of Humans Wearing a Hip Exoskeleton. Front. Bioeng. Biotechnol. 5, 1–11. 10.3389/fbioe.2017.00004 28337434 PMC5340778

[B193] Zanotto D. Stegall P. Agrawal S. K. (2014). “Adaptive Assist-As-Needed Controller to Improve Gait Symmetry in Robot-Assisted Gait Training,” in 2014 IEEE International Conference on Robotics & Automation (ICRA), Hong Kong, China, 31 May-7 June 2014, 724–729. 10.1109/icra.2014.6906934

[B194] Zhang B. Wang S. a. Zhou M. Xu W. (2021). An Adaptive Framework of Real-Time Continuous Gait Phase Variable Estimation for Lower-Limb Wearable Robots. Robotics Aut. Syst. 143, 103842. 10.1016/j.robot.2021.103842

[B195] Zhang C. Liu G. Li C. Zhao J. Yu H. Zhu Y. (2016). Development of a Lower Limb Rehabilitation Exoskeleton Based on Real-Time Gait Detection and Gait Tracking. Adv. Mech. Eng. 8, 168781401562798. 10.1177/1687814015627982

[B196] Zhang J. Fiers P. Witte K. A. Jackson R. W. Poggensee K. L. Atkeson C. G. (2017). Human-in-the-loop Optimization of Exoskeleton Assistance during Walking. Science 356, 1280–1284. 10.1126/science.aal5054 28642437

[B197] Zhao G. Sharbafi M. Vlutters M. Van Asseldonk E. Seyfarth A. (2017). Template Model Inspired Leg Force Feedback Based Control Can Assist Human Walking. IEEE Int. Conf. Rehabil. Robot. 2017, 473–478. 10.1109/ICORR.2017.8009293 28813865

[B198] Zhou Z. Liao Y. Wang C. Wang Q. (2016). “Preliminary Evaluation of Gait Assistance during Treadmill Walking with a Light-Weight Bionic Knee Exoskeleton,” in 2016 IEEE International Conference on Robotics and Biomimetics (ROBIO) (IEEE), Qingdao, China, 3-7 Dec. 2016, 1173–1178. 10.1109/ROBIO.2016.7866484

[B199] Zoss A. B. Kazerooni H. Chu A. Zoss A. B. Kazerooni H. Chu A. (2006). Biomechanical Design of the Berkeley Lower Extremity Exoskeleton (BLEEX). IEEE/ASME Trans. Mechatron. 11, 128–138. 10.1109/TMECH.2006.871087

