# Supplementary material for: Coordination Between Partial Robotic Exoskeletons and Human Gait: A Comprehensive Review on Control Strategies
Source: Front Bioeng Biotechnol. 2022 May 25;10:842294. doi: 10.3389/fbioe.2022.842294 (PMC9174608; doi:10.3389/fbioe.2022.842294)
Supplement: Supplementary file 1 [file Table1.DOCX]

Supplementary Material

# Supplementary Table 1: Device summary.

| *#* | Device | Actuated Joints | Actuator and Low-level control | Coordination Strategy | Experimental Validation | Results |
| --- | --- | --- | --- | --- | --- | --- |
| *Devices coordinated by using Finite-State-Machines* | | | | | | |
| *1* | MIT ankle robot (Blaya and Herr, 2004) | Ankle | Series Elastic Actutator (SEA) with impedance control | Impedance level depending on the gait sate | 2 drop-foot patients | Improvement of swing kinematics and reduction of slap-foot |
| *2* | Quasi-passive compliant stance control orthosis (CSCO) (Shamaei et al., 2013, 2014a, 2014b, 2015) | Knee | Variable stiffness actuator (VSA) with stiffness control | Actuator stiffness depending on the gait state | One healthy subject (Shamaei et al., 2013) | Technically validated |
|  |  |  |  |  | 3 healthy subjects (Shamaei et al., 2014a) | Motor adaptation implied assistance could replace knee function |
|  |  |  |  |  | 3 healthy subjects (Shamaei et al., 2014b) | More natural kinematics pattern when compared with commercial stance control orthosis |
|  |  |  |  |  | 9 healthy subjects (Shamaei et al., 2015) | Knee moment adapted because of the assistance |
| *3* | Polycentric knee exoskeleton (Kim et al., 2015) | Knee | DC motor coupled to Harmonic Drive with impedance or position control | Controller dependent on gait state (impedance control during swing, position control during stance) | Healthy subjects | Technically validated |
| *4* | BioKEX (Zhou et al., 2016) | Knee | Geared motor with impedance control | Tailored impedance level depending on the gait state | 3 healthy subjects | Metabolic cost compensation of wearing the exoskeleton |
| *5* | BioKEX II (Liu and Wang, 2020) | Knee | Geared motor with torque control | FSM to detect gait states, torque generation accordingly | 5 healthy subjects | Technically validated |
|  |  |  |  |  | 1 stroke patient | Symmetry improvements |
| *6* | Active Knee Orthosis from Sao Paolo University (dos Santos et al., 2017) | Knee | SEA with impedance control | Impedance level depending on the gait state | One healthy subject | Technically validated |
| *7* | EICOSI (Wehbi et al., 2017) | Knee | Cable transmission coupled to DC motor with variable impedance control | Impedance level depending on the gait state | 4 healthy subjects | Muscular effort reduced |
| *8* | Active Ankle-Foot orthosis from Yonsei University (Kim et al., 2011) | Ankle | SEA that is shortened or lengthened to induce dorsiflexion or plantarflexion | Torque direction according to gait state | 3 hemiparetic patients | Improvements in temporal-spatial gait parameters by preventing foot drop and toe drag |
| *9* | Anklebot (Forrester et al., 2016; Roy et al., 2013) | Ankle | SEA with impedance control | Variable impedance and assistive torque profiles depending on biomechanical models and current gait state (Roy et al., 2013) | One chronic stroke patient | Reduced foot-drop |
|  |  |  |  | Treadmill rehabilitation vs Seated rehabilitation (Forrester et al., 2016) | 26 chronic hemiparetic patients (n=14 treadmill group; n=12 seated group) | Treadmill rehabilitation more effective six-weeks after intervention |
| *10* | Samsung Multi-functional Ankle Exoskeleton (Choi et al., 2018) | Ankle | DC motor coupled to ball-screw mechanism with torque control | Torque profile depending on gait state | One healthy subject | Technically validated |
| *11* | AlterG/Tibion Bionic Leg (Horst, 2009; Stein et al., 2014; Wong et al., 2012) | Knee | DC motor coupled to a controllable gear mechanism with torque control | Assistance provided during stance | 3 chronic stroke patients (Wong et al., 2012) | Improved balance and functional gait |
|  |  |  |  |  | 24 chronic stroke patients (n=12 CGT group; n=12 robotic therapy group) (Stein et al., 2014) | No significant differences between groups |
| *12* | Knee-ankle-foot orthosis for poliomyelitis subjects (Arazpour et al., 2016) | Knee | Geared DC motor with position control | Restrict flexion during stance, assistance during swing | 7 poliomyelitis subjects | Improved symmetry |
| *13* | Ankle Foot Orthoses from Arizona University (Oymagil et al., 2007; Ward et al., 2011) | Ankle | Robotic Tendon with position control for the tendon compression | Storage/release energy depending on the gait state (Ward et al., 2011) | 3 stroke patients | No significant differences between groups |
|  |  |  |  | Assistive profile triggered by heel-strike and scaled to previous step duration (Oymagil et al., 2007) | 2 healthy subjects | Technically validated |
| *14* | XoSoft (Di Natali et al., 2019) | Hip & knee | Clutch-controlled elastic bands to store/release energy | FSM to detect stance/swing. Clutch action depending on gait state | One chronic stroke patient | Gait and pattern improved |
| *15* | KAIST Pneumatic AFO (Kim et al., 2020) | Ankle | Pneumatic actuator with torque control | FSM to detect stance/swing.  Free motion during stance, assistive torque during swing | 5 hemiparetic drop-foot patients | Ankle dorsiflexion peek improved |
| *16* | Knee Asistive Device from Arizona State University (Chinimilli et al., 2020) | Knee | Rotatory SEA with automatic impedance tuning | FSM and fuzzy logic to detect gait phases and tailor impedance models | 11 healthy subjects | Unilateral assistance leads to stability increase in the non-assisted limb. |
| *17* | Powered Knee Exoskeleton from Georgia Institute of Technology (Lee et al., 2020) | Knee | DC motor coupled to planetary gearbox with torque control | FSM based on FSRs to detect early stance, late stance and swing phases. Torque profile is provided during early stance | 12 healthy subjects assisted during incline and decline walking | Reduced knee extensor activity in assisted leg  ≈50% subjects reduced metabolic cost (compensations in the unassisted limb) |
| *18* | ALLOR (Villa-Parra et al., 2017) | Knee | DC motor with Harmonic Drive with variable impedance control | FSM based on insole pressure sensors to detect gait states and select impedance model | 3 healthy subjects | Technically validated |
| *19* | Bionic Knee Exoskeleton from Peking University (Xu et al., 2019) | Knee | DC motor coupled to a double-stage timing belt transmission with variable impedance control | SVM based on knee angle to detect gait states and select impedance model | 5 healthy subjects | Technically validated |
| *20* | HAL (Kawamoto et al., 2009; Kawamoto and Sankai, 2005) | Hip & Knees | DC motor coupled to a Harmonic Drive with position and velocity control | Cybernic Autonomous Control: gait state detection based on Floor Reaction Force (Kawamoto and Sankai, 2005) | One hemiplegic patient (Kawamoto et al., 2009) | Impaired pattern closer to natural gait |
| *21* | MIT Autonomous ankle (Acosta-Sojo and Stirling, 2022; Mooney et al., 2014c, 2014a, 2014b; Mooney and Herr, 2016) | Ankle (bilateral) | Winch actuator coupled to a cord with work control | Assistive profile triggered by heel-strike | 2 healthy subjects (Mooney et al., 2014c) | Metabolic cost reduced |
|  |  |  |  |  | 7 healthy subjects (Mooney et al., 2014a) | Metabolic cost reduced |
|  |  |  |  |  | 7 healthy subjects carrying a load (Mooney et al., 2014b) | Metabolic cost reduced |
|  |  |  |  |  | 6 healthy subjects (Mooney and Herr, 2016) | Mechanical power provided by the exoskeleton replace power at biological joints |
|  |  |  |  | Assistive profile triggered by push-off (Acosta-Sojo and Stirling, 2022) | 15 healthy subjects (Acosta-Sojo and Stirling, 2022) | 60% subjects reduced Medial Gastrocnemius and 80% increased Tibialis Anterior activity  Muscular response varies between subjects |
| *22* | Portable powered ankle-foot orthosisfrom Illinois University (Shorter et al., 2011) | Ankle | Bidirectional Pneumatic Rotary Actuator with non-regulated torque control | Footswitch signals trigger the torque application | One cauda equine syndrome patient  Three healthy subjects | Technically validated |
| *23* | Ankle Robot (Yeung et al., 2017, 2021) | Ankle | DC motor coupled to a gearbox with torque control | FSM to detect gait states and execute different motor profiles. | Three chronic stroke patients(Yeung et al., 2017) | Reduced drop foot |
|  |  |  |  |  | 31 sub-acute stroke patients (n=17 control; n=14 assisted) (Yeung et al., 2021) | Assisted training led to greater functional gait improvements |
| *24* | KNEXO (Beyl et al., 2011; Knaepen et al., 2014) | Knee | Pneumatic artificial muscle (PAM) with trajectory proxy-based sliding mode control | Target reference triggered by heel-strike and scaled to last step duration | 10 healthy subjects (Beyl et al., 2011) | Technically validated |
|  |  |  |  |  | A Multiple Sclerosis patient (Beyl et al., 2011) | Gait timing symmetry improved |
|  |  |  |  |  | 10 healthy subjects (Knaepen et al., 2014) | No significant EMG differences between low and high assistance |
| *25* | Multi-Functional Soft Ankle Exoskeleton (Xia et al., 2020) | Ankle | Bowden cables coupled to motors with torque control | Torque/force reference triggered by gait events | 5 healthy subjects | Kinematic and EMG results qualitatively confirmed feasibility for foot-drop prevention and propulsion assistance. |
| *26* | Ankle Exoskeleton from Northern Arizona University (Lerner et al., 2018) | Ankle (bilateral) | Bowden cables coupled to DC motors with torque control | Torque profile triggered by gait events detection | 5 Cerebral Palsy patients | Reduced metabolic cost of waling |
| *27* | T-Flex (Gomez-Vargas et al., 2021) | Ankle | Artificial tendons actuated by servomotors with torque control | Gait phases detected by Inertial Sensor placed in the foot | 10 stroke patients | Biomechanical improvements in 70% of the patients |
| *28* | NIH Powered Knee Exoskeleton (Lerner et al., 2017b, 2017a) | Knee (bilateral) | DC motor coupled to a gear box with torque control | Torque profiles triggered by a FSM that detects gait states based on insole pressure sensors and inertial sensors | 1 Cerebral Palsy patient with crouch gait (Lerner et al., 2017b) | Knee extension improvements  No changes in knee extensors activity |
|  |  |  |  |  | 4 Cerebral Palsy patient with crouch gait (Lerner et al., 2017a) | Reduced crouch gait  Increased knee flexors activity |
| *29* | Soft-inflatable exosuit from Arizona State University (Sridar et al., 2018, 2020) | Knee extension  (bilateral) | Inflatable actuator with pressure control | Pressure profiles triggered by gait event detection based on insole sensors | 3 healthy subjects (Sridar et al., 2018) | Reduced quadriceps activity |
|  |  | Knee extension |  |  | 7 healthy subjects (Sridar et al., 2020) | Reduced quadriceps activity |
|  |  |  |  |  | 3 stroke patients (Sridar et al., 2020) | Knee extension promoted  Timed up and go test reduced |
| *30* | Ankle Exoskeleton from Carnegie Mellon University (Jackson and Collins, 2015, 2019; Steele et al., 2017; Witte et al., 2015; Zhang et al., 2017) | Ankle | Cable driven SEA with torque control (Witte et al., 2015) | Torque profile triggered by heel strike and scaled to last step duration | One healthy subject (Witte et al., 2015) | Technically validated |
|  |  |  | Cable driven SEA with torque control or work control (Jackson and Collins, 2015; Steele et al., 2017) |  | 8 healthy subjects (Jackson and Collins, 2015) | Effort reduced  Metabolic cost reduced with work assistance but increased with torque assistance |
|  |  |  |  |  | 10 healthy patients(Steele et al., 2017) | Reduced muscular recruitment  Muscular coordination affected by assistance |
|  |  |  | Cable driven SEA with human-in-the-loop metabolic cost optimized torque profile tracking (Zhang et al., 2017) | Torque profile triggered by heel strike and scaled to last step duration | 11 healthy subjects | Metabolic cost reduced with optimized assistive torque |
|  |  |  | Cable driven SEA with human-in-the-loop EMG optimized torque profile tracking (Jackson and Collins, 2019) |  | 10 healthy subjects | Soleus activity and metabolic cost reduced |
| *31* | Ankle Exoskeleton from Ghent University (Galle et al., 2017; Malcolm et al., 2018) | Ankle (bilateral) | PAM with work control | Torque profile triggered by heel strike and scaled to last step duration | 10 healthy subjects (Galle et al., 2017) | Metabolic cost reduced |
|  |  |  |  |  | 11 healthy subjects (Malcolm et al., 2018) | Metabolic cost reduction most depending on the assistance symmetry than the total assistance |
| *32* | Unilateral Exoskeleton Robot from Shenzhen University (Wei et al., 2019) | Hip & knee | DC motor coupled to a Harmonic Drive with human- in-the-loop position control | Trajectory triggered by heel strike and scaled to last step duration | 3 hemiparetic subjects | Joints’ ranges of motion improved |
| *33* | Samsung Hip Exoskeleton (Lim et al., 2015) | Hip (bilateral) | Tendon-based remote mechanism with torque control | FSM to detect gait states. Torque profile triggered by gait events and scaled to last step duration | One healthy subject | Gait regularity improved |
| *34* | Assistive Knee Brace from Hong-Kong University (Ma et al., 2018) | Knee | DC motor coupled to a magneto-rheological brake with impedance control | FSM with Fuzzy Expert System to detect gait state and tailor impedance model. Kinematic reference pattern is adapted to individual gait features. | One post-surgical patient | Active participation of the weak leg promoted |
| *35* | Powered Knee Orthosis from Vrije Universiteit Brussel (Bacek et al., 2021) | Knee | SEA with torque control | FSM to detect gait states. Torque profile triggered by heel strike and scaled to last step duration | 7 healthy subjects | No changes in metabolic consumption.  Increased muscular activity in both legs.  Joint kinematics adaptation to assistance |
| Devices coordinated by estimating continuous gait phase | | | | | | |
| *36* | Soft Exosuit from Harvard University (Awad et al., 2017a, 2017b; Bae et al., 2015, 2018a, 2018b; Ding et al., 2016b, 2016a, 2017, 2018; Kim et al., 2019; Lee et al., 2016, 2017a; Siviy et al., 2020) | Ankle | Bowden Cables coupled to motors with cable position control to force delivery (Awad et al., 2017b, 2017a; Bae et al., 2015) | Gait phase estimation from the duration of previous steps | 3 chronic stroke patients (Bae et al., 2015) | Gait symmetry improved |
|  |  |  |  |  | 9 chronic stroke patients (Awad et al., 2017b) | Reduced propulsion interlimb asymmetry and metabolic cost of walking |
|  |  |  |  |  | 8 chronic stroke patients (Awad et al., 2017a) | Reduced hip hiking and circumduction |
|  |  |  | Bowden Cables coupled to motors with offline human-in-the-loop optimized Force profile tracked by admittance control (Siviy et al., 2020) |  | 6 chronic stroke patients (Siviy et al., 2020) | Delivered positive power contributed to total ankle power |
|  |  |  | Bowden Cables coupled to motors with Iterative Force-based cable trajectory tracking (Bae et al., 2018a) |  | 7 chronic stroke patients (Bae et al., 2018a) | Reduced asymmetry in CoM power generation |
|  |  |  | Bowden Cables coupled to motors with Iterative Force-based cable trajectory tracking (cascade position-velocity loop) (Bae et al., 2018b) |  | 3 chronic stroke patients (Bae et al., 2018b) | Electrical power consumption reduced. Similar effects on patients compared with previous studies |
|  |  | Hip | Bowden Cables coupled to motors with Iterative Force-based cable trajectory tracking (Ding et al., 2016a, 2016b) |  | 8 healthy subjects carrying loads (Ding et al., 2016a, 2016b) | Technically validated. Reduction of metabolic cost |
|  |  | Hip & Ankle (bilateral) | Bowden Cables coupled to motors with cable position control to force delivery (Lee et al., 2016) |  | 7 healthy subjects (Lee et al., 2016) | Technically validated. Reduction of metabolic cost |
|  |  |  | Bowden Cables coupled to motors with Iterative Force-based cable trajectory tracking (Ding et al., 2017) |  | 8 healthy subjects carrying loads (Ding et al., 2017) | Metabolic cost and muscular activity reduction without alter human kinematics |
|  |  | Hip (Bilateral) | Bowden Cables coupled to motors with force profile tracking by admittance control (Kim et al., 2019; Lee et al., 2017a) |  | 3 healthy subjects (Lee et al., 2017a) | Technically validated |
|  |  |  |  |  | 9 healthy subjects (Kim et al., 2019) | Reduced metabolic cost of walking and running |
|  |  |  | Bowden Cables coupled to motors with Human-in-the-loop optimized force profile tracked by admittance control (Ding et al., 2018) |  | 8 healthy subjects (Ding et al., 2018) | Reduced metabolic cost (more efficient that previous works) |
| *37* | Soft Robotic Suit from Kyushu University (Jin et al., 2017) | Hip (bilateral) | Elastic belt coupled to geared motor with torque control | Gait phase estimation from the duration of previous steps. Torque profile key-points defined at certain gait phases. | 9 elderly subjects | Gait characteristics improved and metabolic cost reduced |
| *38* | Hip exoskeleton from Georgia Institute of Technology (Kang et al., 2019, 2020; Young et al., 2017) | Hip (bilateral) | Pneumatic piston actuator with burst control (Young et al., 2017) | Gait phase estimation from the duration of previous steps (Young et al., 2017) | 10 healthy subjects | Metabolic cost reduction |
|  |  |  | SEA with torque control (Kang et al., 2019, 2020) | Gait phase estimation from the duration of previous steps (Kang et al., 2019) | 10 healthy subjects | Metabolic cost reduction |
|  |  |  |  | Neural network model based on hip and thigh angles to estimate gait phase and command torque profile (Kang et al., 2020) | 10 healthy subjects | Technically validated |
| *39* | UPEC Active Ankle Foot Orthosis (Huo et al., 2019) | Ankle | Geared DC motor with Trajectory tracking by adaptive proxy-based sliding mode control | Trajectory generation depending on gait phase estimation based on time between key-events | 2 healthy subjects  2 paretic patients | Technically validated |
| *40* | WAXO (Bougrinat et al., 2019) | Ankle | Bowden Cables coupled to a DC motor with open-loop torque control | Gait phase estimation from the duration of previous steps. Plantar-flexion torque profile triggered at certain phase. | One healthy subject | Decrease of the Gastrocnemius activity |
| *41* | Myosuit (Haufe et al., 2021) | Knee (bilateral) | Bowden Cables coupled to a DC motor with force control | Gait phase estimation from the duration of previous steps. Force profile defined at certain gait phases to reinforce knee extension. | 8 healthy suject (3 sessions of 20 minutes) | Kinematic and muscular effects after the first minute; energetic benefits after 10 minutes. |
| *42* | ALEX II (Lenzi et al., 2013) | Hip | Rotatory motor coupled to a gear box with torque control | Gait phase detection relying on AO and cycle duration | 10 healthy subjects | Reduced muscular activation at hip and ankle joint |
| *43* | ALEX III (Zanotto et al., 2014) | Hip & Knee | Rotatory motor coupled to a gear box with force-field control | AO to detect gait phase and synchronize the application of the target trajectory | 3 healthy subjects | Improved gait symmetry |
| *44* | AO to estimate assistive torque (Ronsse et al., 2011) | Hip & Knee (bilateral) | LOPES Platform (Veneman et al., 2007) | Assistive torque prediction by an AO and non-linear filter using the hip angle | 9 healthy subjects | Energy expenditure reduction with assistance (no reduction compared to free walking) |
| *45* | Cyberlegs Active Pelvis Orthosis (Giovacchini et al., 2015; Grazi et al., 2015; Ruiz Garate et al., 2016) | Hip (bilateral) | SEA with torque control | Assistive torque prediction by an AO and non-linear filter using the hip angle (Giovacchini et al., 2015) | One healthy subject | Technically validated |
|  |  |  |  | Proportional myoelectric control (gastrocnemius) during ankle push-off (determined by an AO with vGRF input) (Grazi et al., 2015) | One healthy subject | Muscular activity reduction |
|  |  |  |  | Gait phase estimation by an AO based on hips angle   1. Joint torque estimation directly from dynamic primitives (Ruiz Garate et al., 2016) 2. Joint torque estimation from muscular activity primitive through human model (Ruiz Garate et al., 2016) | 7 healthy subjects | Metabolic cost reduction compared to unassisted mode |
| *46* | Cyberlegs Active Pelvis Orthosis & Knee-Ankle Foot Orthosis (Ruiz Garate et al., 2017; Sanz-Morere et al., 2018; Yan et al., 2015) | Hip, Knee & Ankle | SEA with position and torque control (hip & knee) Variable stiffness actuator (ankle) | Hip assistive torque prediction by an AO and non-linear filter using the hip angle (Giovacchini et al., 2015). Finite state machine for Knee and ankle (Yan et al., 2015) | 2 healthy subjects (Yan et al., 2015) | Technically validated |
|  |  |  |  |  | 2 amputee (Sanz-Morere et al., 2018) | Technically validated |
|  |  |  |  | Gait phase estimation by an AO based on the unassisted hip angle. Joint torque estimation from muscular activity primitive through human model (Ruiz Garate et al., 2017) | 7 healthy subjects | Increased walking velocity with assistance |
| *47* | Samsung GEMS (Lee et al., 2017b, 2017c, 2019; Seo et al., 2016) | Hip (bilateral) | Geared motor with torque control | Gait phase estimation by an PSAO based on the hip angle | 5 healthy subjects (Seo et al., 2016) | Metabolic cost reduced, step length and stride time increased with assistance |
|  |  |  |  |  | 30 elderly subjects (Lee et al., 2017b) | Gait function improved  Muscular effort and metabolic cost reduced |
|  |  |  |  |  | 30 elderly subjects (Lee et al., 2017c) | Maximum foot force and pressure distribution increased |
|  |  |  |  |  | 26 chronic stroke patients (n=14 experimental group; n=12 control group) (Lee et al., 2019) | Better results with GEMS therapy than conventional one |
| *48* | Knee Exoskeleton from National University of Singapore (Aguirre-Ollinger et al., 2019; Aguirre-Ollinger and Yu, 2021) | Knee | Bowden cables actuated by SEA with force control | Gait phase estimation by an AO based on the impaired hip angle. Assistive torque synchronized with the maximum unimpaired knee flexion phase (Aguirre-Ollinger et al., 2019). | 9 healthy subjects wearing an artificial knee flexion impairment | No significant differences between impaired and unimpaired leg |
|  |  |  |  | Gait phase estimation by an AO based on the impaired hip angle. Assistive torque synchronized by delaying 180º the phase of unimpaired gait events. (Aguirre-Ollinger and Yu, 2021) | 8 healthy subjects wearing an artificial knee flexion impairment | Symmetry improvements in the target gait event to levels comparable to unobstructed gait. |
| *49* | Hip Exoskeleton from Sydney University of Technology (Aguirre-Ollinger, 2013, 2015) | Hip | DC motor coupled to harmonic drive with admittance control | Gait phase estimation by an AO based on the assisted hip angle  Assistive torque from users’ EMG envelope (Aguirre-Ollinger, 2013) | 2 healthy subjects | Technically validated |
|  |  |  |  | Gait phase estimation by an AO based on the assisted hip angle. Assistive torque from a biomechanical model (Aguirre-Ollinger, 2015) | 10 healthy subjects | Muscular effort reduced |
| *50* | Hip & Knee exoskeleton from Nanjing Institute of Technology (Han et al., 2019) | Hip & Knee | Geared motor (cable transmission at knee) with admittance control | Gait phase estimated by an AO based on the joint torque  Sinusoidal torque profile | One healthy subject | Technically validated |
| *51* | Achilles Ankle Exoskeleton (van Dijk et al., 2017) | Ankle (bilateral) | Linear SEA with torque control | Gait phase estimated by an AO based on the foot pressure  Spring simulated torque profile | 4 healthy subjects | No differences in metabolic cost |
| *52* | Lightweight Hip Exoskeleton from University of Utah (Ishmael et al., 2019) | Hip | SEA with torque control | Gait phase estimated by an AO based on the joint angle  Gaussian torque profile application synchronized with gait phase | One unilateral above-knee amputee subject | Reduced metabolic cost of walking |
| *53* | Sharif powered exoskeleton (Talatian et al., 2021) | Knee | SEA with torque control | Gait phase estimated by an AO based on knee angle.  Assistive torque profile from knee flexor-extensor EMG | One healthy subject | Reduced muscular activity of knee flexor |
| *54* | Underactuated Soft Hip Exosuit from Heidelberg University (Tricomi et al., 2021) | Hip (bilateral) | DC motor coupled to artificial tendons with position control | Gait phase estimated by an AO based on the hip angle.  Position reference is a sinusoidal function of the estimated gait phase | 6 healthy subjects | No disturbances in gait kinematics  Reduced muscular effort in hip flexors |
| *55* | ANdROS (Aoyagi et al., 2007; Unluhisarcikli et al., 2011) | Hip & Knee | DC motor coupled to gear box with impedance control | Gait phase estimation using the movement of both knees and hips (Aoyagi et al., 2007) | No subjects validation | Technically validated |
| *56* | Portable powered ankle foot orthosis from Illinois University (Li et al., 2011) | Ankle | Bidirectional Pneumatic Rotary Actuator with non-regulated torque control | Continuous phase estimation based on cross-correlation with a learned model | Five healthy subjects  One neurological patient | Coordination strategy technically validated |
| *57* | Single-joint hip exoskeleton from Xi’an Jiaotong University (Zhang et al., 2021) | Hip | DC motor coupled to gear box with torque control | Continuous phase estimation based on particle swarm optimization and learning user kinematics | 7 healthy subjects | Technically validated |
| Devices coordinated based on user kinematics | | | | | | |
| *58* | Powered Knee Orthosis from Hong Kong University (Lai et al., 2013) | Knee | Geared motor with position control | Knee trajectory estimated from hip velocity | One healthy subject | Technically validated |
| *59* | PH-Exos (Wu et al., 2015) | Hip (bilateral) | Bowden cables coupled to servo motor with fuzzy velocity control | Motion intention estimated from pressure measurement at Rectus Femoris and Biceps Femoris | One healthy subjects | Technically validated |
| *60* | Ankle Exoskeleton from Northern Arizona University (Conner et al., 2020; Fang and Lerner, 2021; Gasparri et al., 2019; Orekhov et al., 2020) | Ankle (bilateral) | Bowden Cables coupled to DC motors with assistive torque control | Assistance is proportional to ankle moment (Fang and Lerner, 2021; Gasparri et al., 2019; Orekhov et al., 2020) | 1 Healthy subject and 4 Cerebral Palsy patients (Gasparri et al., 2019) | Technically validated |
|  |  |  |  |  | 1 Healthy subject and 2 Cerebral Palsy patients (Gasparri et al., 2019) | Reduced metabolic cost of waling |
|  |  |  |  |  | 6 Cerebral Palsy patients (Orekhov et al., 2020) | Increased gait speed  Reduced metabolic cost of waling  Reduced soleus muscle activity |
|  |  |  |  |  | 7 Cerebral Palsy patients (Fang and Lerner, 2021) | Step length biofeedback plus assistance improved biomechanics and did not affect muscle activity compared with only assistance |
|  |  |  | Bowden Cables coupled to DC motors with resistive torque control | Resistance is proportional to ankle moment (Conner et al., 2020) | 8 Spastic Cerebral Palsy patients | Increased plantar-flexor activity and decreased dorsiflexor activity |
| *61* | Untethered Ankle Exoskeleton from Northern Arizona University (Orekhov et al., 2021) | Ankle (bilateral) | Bowden Cables coupled to DC motors with assistive torque control | Assistance is proportional to ankle moment during stance state of the gait | 6 healthy subjects (inclined walking) | Reduced metabolic cost of waling |
|  |  |  |  |  | 5 Cerebral Palsy patients (stairs ascent) | Increased kinematic outcomes without affecting the metabolic consumption |
| *62* | Echo-Control (Wang et al., 2013) | Software development | | Gait phase estimation of the affected limb based on the kinematics of the sound leg | Three healthy subjects | Technically validated |
| *63* | HAL – Echo control (Kawamoto et al., 2014, 2015) | Hip & Knee (bilateral) | DC motor coupled to a Harmonic Drive with position and velocity control | Replication of sound leg movement during swing (Kawamoto et al., 2014) | One chronic stroke patient | Significantly improved gait symmetry |
|  |  |  |  | Replication of sound leg movement during swing and stance (Kawamoto et al., 2015) | One chronic stroke patient | Significantly improved gait symmetry |
| *64* | Powered gait training system (Nguyen et al., 2013) | Hip & Knee | Linear motor actuators with position control | Healthy leg trajectory delayed | One healthy subject | Technically validated |
| *65* | Lower limb exoskeleton from Harbin Institute of Technology (Zhang et al., 2016) | Hip & Knee | SEA with position control | Healthy leg trajectory delayed | No subjects validation | Technically validated |
| *66* | BioComEx (Baser et al., 2020) | Hip, Knee & Ankle (bilateral) | VSA (ankle) and SEA (hip and knee) with position (assisted leg) and impedance (unassisted leg) control | Healthy leg trajectory delayed | One unilateral impaired subject | Technically validated |
| *67* | Wirerope-driven exoskeleton from South China University of Technology (Xie and Huang, 2019) | Hip & Knee | Wire-rope couple to DC servo motor with position control | Replication of the movement of the healthy side recorded by video-cameras | 10 hemiplegic patients | Similar range of motion at impaired and unimpaired joints |
| *68* | Knee-ankle foot orthosis from Spanish National Research Council (Lora-Millan et al., 2020) | Knee | DC motor coupled to a Harmonic Drive with impedance control | Movement of the non-actuated leg is used to synchronize the assistance applied over the assisted leg | 12 healthy subjects | Improved symmetry  Muscular activity was mostly unaffected although were more frequently increased than decreased |
| 69 | AIDER (Peng et al., 2020) | Hip & Knee (bilateral) | DC motor coupled to a Harmonic Drive with position control using actor-critic neural network | Sound limb is considered the leader and the exoskeleton the follower according to the leader-follower multi-agent system framework | 3 healthy subjects | Technically validated |
| *70* | PCA-CLME (Vallery et al., 2007; Vallery and Buss, 2006) | Hip & Knee (bilateral) | LOPES platform (Veneman et al., 2007) | PCA of healthy leg movements (Vallery and Buss, 2006) | 8 healthy subjects simulating above knee amputation (Vallery et al., 2007) | Technically validated |
| *71* | BLUE-CLME (Vallery et al., 2009) | Hip & Knee (bilateral) | LOPES platform (Veneman et al., 2007) | BLUE-CLME of healthy leg movements | 9 healthy subjects walking over a treadmill | Better results than PCA-CLME by reducing results variability |
| *72* | HAL - CLME (Hassan et al., 2012, 2018) | Hip & Knee | DC motor coupled to harmonic Drive with position control | PCA-CLME with the movement of the healthy leg and a cane (Hassan et al., 2012) | One healthy subject | Technically validated |
|  |  |  |  | PCA-CLME with cane vs CAC (Hassan et al., 2018) | 5 hemiparetic patients | 3/5 patients were able to walk  Grater increasing in the Range of Motion with CAC  More symmetric gait with PCA-CLME |
| *73* | PCA to estimate motor torque primitive (Nunes et al., 2018) | Knee | SEA with torque control | PCA of motor torque computed from kinematic data using OpenSim | One healthy subject with an active knee orthosis | Knee position profile not hampered by the use of the exoskeleton |
| *74* | ALEX (Banala et al., 2007, 2009) | Hip & Knee | Linear actuator with torque control | Force field control around a path | 3+3 healthy subjects (experimental + control group) (Banala et al., 2007) | Reduced deviation from prescribed pattern |
|  |  |  |  |  | 2 chronic stroke patients (Banala et al., 2009) | Improved gait pattern |
| *75* | ALEX II (Srivastava et al., 2015; Winfree et al., 2011) | Hip & Knee | Rotatory motor coupled to a gear box with torque control | Force field control with tangencial force to advance across the path | 6 healthy subjects (Winfree et al., 2011) | Foot trajectory closer to prescribed. |
|  |  |  |  |  | 9 chronic stroke patients (Srivastava et al., 2015) | Improved paretic gait pattern after therapy |
| *76* | C-ALEX (Hidayah et al., 2020; Jin et al., 2015, 2018) | Hip & Knee | Bowden cable with torque control | Force field control with tangencial force to advance across the path | 6 healthy subjects (Jin et al., 2015) | Foot trajectory closer to prescribed. |
|  |  |  |  |  | 9 healthy subjects (Jin et al., 2018) | Step height increased after use, confirming gait retraining |
|  |  |  |  |  | 10 chronic hemiparetic stroke patients (Hidayah et al., 2020) | Step length and height improved |
| *77* | Vanderbilt Path/Flow control (Martinez et al., 2018, 2019; Martínez et al., 2020) | Hip & Knee (Bilateral) | Indego commercial exoskeleton (Farris et al., 2011) | Path control (Martinez et al., 2018) | Five healthy subjects | Leg guidance provided while enabling step-to-step variability in timing and length |
|  |  |  |  | Flow control (Martinez et al., 2019) | Five healthy patients | Lower guidance error and disturbance combination than path control  Combined guidance and assisting behaviour |
|  |  | Knee | Bowden Cables coupled to motors with torque control | Single Joint flow control (Martínez et al., 2020) | Three unilaterally impaired patients | Improved gait kinematic pattern |
| *78* | VPP model for exoskeletons control (Sharbafi et al., 2018; Zhao et al., 2017) | Hip & Knee (bilateral) | LOPES-II platform (Meuleman et al., 2016) | Target torque pattern from VPP model for stable walking (Zhao et al., 2017) | 4 healthy subjects | Unaltered hip and knee kinematics  Gastrocnemius activity and metabolic cost reduced |
|  |  | Biarticular (hip & knee, bilateral) | Compliant adjustable actuator with stiffness modulation | VPP model for stable walking (Sharbafi et al., 2018) | Simulation with a musculoskeletal model | Muscular activity and energy cost of walking reduced |
| Devices coordinated by using muscular activity | | | | | | |
| *79* | HAL (Kawamoto et al., 2010; Nilsson et al., 2014; Sczesny-Kaiser et al., 2019; Tan et al., 2018, 2020; Watanabe et al., 2014, 2020) | Hip & Knee | DC motor coupled to a Harmonic Drive with torue control | Assistance triggered by knee flexor activity | One hemiplegic patient – Standing experiment (Kawamoto et al., 2010) | Increased range of motion of paretic knee. |
|  |  |  |  | Cybernic Autonomous Control (CAC): muscular activity triggered predefined movements  Cybernic Voluntary Control (CVC): torque proportional to muscular activity of flexors and extensors of hips and knee | 8 stroke patients (Nilsson et al., 2014) | Improved walking ability (10MWT and FAC scores) |
|  |  |  |  | CVC & CAC vs CGT (Conventional Gait Training) | 22 sub-acute stroke patients (n=11 CGT group; n=11 HAL group) (Watanabe et al., 2014) | Significantly greater FAC improvements in HAL group than in CGT group |
|  |  |  |  |  | 22 acute stroke patients (n=10 CGT group; n=12 HAL group) (Watanabe et al., 2020) |  |
|  |  |  |  | CVC vs CGT | 18 chronic stroke patients (n=9 CVC+CPT; n=9 CPT+CVC) (Sczesny-Kaiser et al., 2019) | No significant differences between CVC and CGT  Mixed approach CVC and CGT improved gait and balance |
|  |  |  |  | CVC | 8 acute stroke patients (Tan et al., 2018) | Reduced differences between lateral muscular synergies after intervention |
|  |  |  |  | CVC vs CGT | 20 acute stroke patients (n=9 CGT group; n=11 HAL group) (Tan et al., 2020) | Symmetry of muscular synergies timing improved respect to CGT group |
| *80* | Ankle foor orthosis form University of Michigan (Cain et al., 2007; Gordon and Ferris, 2007; Kao et al., 2010; Kinnaird and Ferris, 2009; Koller et al., 2015, 2017; Sawicki and Ferris, 2008) | Ankle | PAM with plantar flexor torque control | Proportional myoelectric control from soleus EMG (Gordon and Ferris, 2007) | 10 healthy subjects | Reduced soleus recruitment |
|  |  |  |  | Proportional myoelectric control from Medial Gastrocnemius EMG (Kinnaird and Ferris, 2009) | 10 healthy subjects | Reduced soleus and medial gastrocnemius activity |
|  |  |  |  | 1. Proportional soleus myoelectric control 2. Footswitch on/off control (Cain et al., 2007) | 6 healthy subjects each experimental group | Muscular activity reduced and kinematics closer to normal with myoelectric control |
|  |  |  |  | Proportional myoelectric control from soleus EMG with fixed gain (Kao et al., 2010) | 11 healthy subjects | Reduced soleus activity and different kinematic patterns; similar ankle torque pattern with and without exoskeleton. |
|  |  | Ankle (bilateral) |  | Proportional myoelectric control from soleus EMG with fixed gain (Sawicki and Ferris, 2008) | 9 healthy subjects | Reduced soleus activity, similar to normal ankle pattern and reduction of metabolic cost. |
|  |  |  |  | Proportional myoelectric control from soleus EMG with adaptive gain (Koller et al., 2015) | 8 healthy subjects | Metabolic reduction with a lower adaptation time. |
|  |  |  |  | (1) Proportional myoelectric control  (2) Torque profile triggered by heel strikes (Koller et al., 2017) | 8 healthy subjects | Metabolic reduction, no differences between strategies  Reduced ankle power with mechanically triggered assistance compared to myoelectric control. |
| *81* | Knee-ankle foot orthosis from University of Michigan (Sawicki and Ferris, 2009) | Knee & Ankle | PAMs with torque control | 1. Direct proportional myoelectric control 2. Myoelectric control with flexor inhibition | 3 healthy subjects | Myoelectric control with flexor inhibition supposed a more natural gait.  Knee torques are ineffective due to the structure of the device. |
| *82* | Powered ankle exoskeleton from North Carolina State University (McCain et al., 2019; Takahashi et al., 2015) | Ankle | PAMs with plantarflexion torque control | Proportional myoelectric propulsion (PMP) from soleus EMG (Takahashi et al., 2015) | 5 chronic stroke patients | Increased paretic plantarflexor torque |
|  |  |  |  | PMP from soleus EMG increased by gait velocity (McCain et al., 2019) | 6 chronic stroke patients | Increased paretic plantarflexor power |
| *83* | TUPLEE (Fleischer and Hommel, 2008) | Knee | Linear actuator with torque control | Torque reference calculated by model of flexors and extensors knee muscle | One healthy subject | Technically validated |
| *84* | Neuromechanical model for exoskeletons control (Durandau et al., 2019) | Hip, knee & ankle (bilateral) | H2 prototype (Bortole et al., 2015) | Joint torque reference calculated by neuromechanical model of 12 MTU’s | 4 healthy subjects  2 chronic stroke patients  1 SCI patient | Functionally validated  Muscular effort reduced with increasing assistance level |
| Devices coordinated by Central Pattern Generators | | | | | | |
| *85* | CPG for Hemiparesis Rehabilitation (Mishra et al., 2014) | Software development | | CPG | Simulation | Technically validated |
| *86* | Curara (Mizukami et al., 2018; Tsukahara and Hashimoto, 2016) | Hip & Knee (bilateral) | Geared servomotor with position control | CPG | One healthy subject simulating hemiplegic gait (Tsukahara and Hashimoto, 2016) | Improved symmetry |
|  |  |  |  |  | 11 chronic stroke patients (Mizukami et al., 2018) | Improved symmetry and gait velocity |
| *87* | Coordinated Admittance Control (Gui et al., 2017) | Software development | | CPG based on EMG data | 4 healthy subjects | Technically validated |
| *88* | Achilles Ankle Exoskeleton (Dzeladini et al., 2016; Tamburella et al., 2020) | Ankle (bilateral) | Linear SEA with torque control | CPG based on floor contact events | 2 healthy subjects (Dzeladini et al., 2016) | Reduced metabolic cost of walking and muscle activity |
|  |  |  |  |  | 4 incomplete spinal cord injury patients during 10-session training (Tamburella et al., 2020) | Improved gait speed and endurance during assisted walking |

# References

Acosta-Sojo, Y., and Stirling, L. (2022). Individuals differ in muscle activation patterns during early adaptation to a powered ankle exoskeleton. *Appl. Ergon.* 98, 103593. doi:10.1016/j.apergo.2021.103593.

Aguirre-Ollinger, G. (2013). Learning muscle activation patterns via nonlinear oscillators: Application to lower-limb assistance. in *2013 IEEE/RSJ International Conference on Intelligent Robots and Systems* IEEE International Conference on Intelligent Robots and Systems., ed. N. Amato (IEEE), 1182–1189. doi:10.1109/IROS.2013.6696500.

Aguirre-Ollinger, G. (2015). Exoskeleton control for lower-extremity assistance based on adaptive frequency oscillators: adaptation of muscle activation and movement frequency. *Proc. Inst. Mech. Eng. H.* 229, 52–68. doi:10.1177/0954411914567213.

Aguirre-Ollinger, G., Narayan, A., and Yu, H. (2019). Phase-Synchronized Assistive Torque Control for the Correction of Kinematic Anomalies in the Gait Cycle. *IEEE Trans. Neural Syst. Rehabil. Eng.* 27, 2305–2314. doi:10.1109/TNSRE.2019.2944665.

Aguirre-Ollinger, G., and Yu, H. (2021). Lower-Limb Exoskeleton With Variable-Structure Series Elastic Actuators: Phase-Synchronized Force Control for Gait Asymmetry Correction. *IEEE Trans. Robot.* 37, 763–779. doi:10.1109/TRO.2020.3034017.

Aoyagi, D., Ichinose, W. E., Harkema, S. J., Reinkensmeyer, D. J., and Bobrow, J. E. (2007). A robot and control algorithm that can synchronously assist in naturalistic motion during body-weight-supported gait training following neurologic injury. *IEEE Trans. Neural Syst. Rehabil. Eng.* 15, 387–400. doi:10.1109/TNSRE.2007.903922.

Arazpour, M., Ahmadi, F., Bahramizadeh, M., Samadian, M., Mousavi, M. E., Bani, M. A., et al. (2016). Evaluation of gait symmetry in poliomyelitis subjects: Comparison of a conventional knee-ankle-foot orthosis and a new powered knee-ankle-foot orthosis. *Prosthet. Orthot. Int.* 40, 689–695. doi:10.1177/0309364615596063.

Awad, L. N., Bae, J., Kudzia, P., Long, A., Hendron, K., Holt, K. G., et al. (2017a). Reducing Circumduction and Hip Hiking During Hemiparetic Walking Through Targeted Assistance of the Paretic Limb Using a Soft Robotic Exosuit. *Am. J. Phys. Med. Rehabil.* 96, S157–S164. doi:10.1097/PHM.0000000000000800.

Awad, L. N., Bae, J., O’Donnell, K., De Rossi, S. M. M., Hendron, K., Sloot, L. H., et al. (2017b). A soft robotic exosuit improves walking in patients after stroke. *Sci. Transl. Med.* 9, eaai9084. doi:10.1126/scitranslmed.aai9084.

Bacek, T., Moltedo, M., Serrien, B., Langlois, K., Vanderborght, B., Lefeber, D., et al. (2021). Human Musculoskeletal and Energetic Adaptations to Unilateral Robotic Knee Gait Assistance. *IEEE Trans. Biomed. Eng.* PP, 1–1. doi:10.1109/TBME.2021.3114737.

Bae, J., Awad, L. N., Long, A., O’Donnell, K., Hendron, K. L., Holt, K. G., et al. (2018a). Biomechanical mechanisms underlying exosuit-induced improvements in walking economy after stroke. *J. Exp. Biol.* 221. doi:10.1242/jeb.168815.

Bae, J., De Rossi, S. M. M., O’Donnell, K., Hendron, K. L., Awad, L. N., Teles Dos Santos, T. R., et al. (2015). A soft exosuit for patients with stroke: Feasibility study with a mobile off-board actuation unit. in *IEEE International Conference on Rehabilitation Robotics* doi:10.1109/ICORR.2015.7281188.

Bae, J., Siviy, C., Rouleau, M., Menard, N., Odonnell, K., Geliana, I., et al. (2018b). A Lightweight and Efficient Portable Soft Exosuit for Paretic Ankle Assistance in Walking After Stroke. in *2018 IEEE International Conference on Robotics and Automation (ICRA)* IEEE International Conference on Robotics and Automation ICRA. (IEEE), 2820–2827. doi:10.1109/ICRA.2018.8461046.

Banala, S. K., Agrawal, S. K., and Scholz, J. P. (2007). Active Leg Exoskeleton (ALEX) for Gait Rehabilitation of Motor-Impaired Patients. in *2007 IEEE 10th International Conference on Rehabilitation Robotics* International Conference on Rehabilitation Robotics ICORR. (IEEE), 401–407. doi:10.1109/ICORR.2007.4428456.

Banala, S. K., Seok Hun Kim, Agrawal, S. K., and Scholz, J. P. (2009). Robot Assisted Gait Training With Active Leg Exoskeleton (ALEX). *IEEE Trans. Neural Syst. Rehabil. Eng.* 17, 2–8. doi:10.1109/TNSRE.2008.2008280.

Baser, O., Kizilhan, H., and Kilic, E. (2020). Employing variable impedance (stiffness/damping) hybrid actuators on lower limb exoskeleton robots for stable and safe walking trajectory tracking. *J. Mech. Sci. Technol.* 34, 2597–2607. doi:10.1007/s12206-020-0534-4.

Beyl, P., Knaepen, K., Duerinck, S., Van Damme, M., Vanderborght, B., Meeusen, R., et al. (2011). Safe and Compliant Guidance by a Powered Knee Exoskeleton for Robot-Assisted Rehabilitation of Gait. *Adv. Robot.* 25, 513–535. doi:10.1163/016918611X558225.

Blaya, J. A., and Herr, H. (2004). Adaptive control of a variable-impedance ankle-foot orthosis to assist drop-foot gait. *IEEE Trans. Neural Syst. Rehabil. Eng.* 12, 24–31. doi:10.1109/TNSRE.2003.823266.

Bortole, M., Venkatakrishnan, A., Zhu, F., Moreno, J. C., Francisco, G. E., Pons, J. L., et al. (2015). The H2 robotic exoskeleton for gait rehabilitation after stroke: early findings from a clinical study. *J. Neuroeng. Rehabil.* 12, 54. doi:10.1186/s12984-015-0048-y.

Bougrinat, Y., Achiche, S., and Raison, M. (2019). Design and development of a lightweight ankle exoskeleton for human walking augmentation. *Mechatronics* 64, 102297. doi:10.1016/j.mechatronics.2019.102297.

Cain, S. M., Gordon, K. E., and Ferris, D. P. (2007). Locomotor adaptation to a powered ankle-foot orthosis depends on control method. *J. Neuroeng. Rehabil.* 4, 1–13. doi:10.1186/1743-0003-4-48.

Chinimilli, P. T., Rezayat Sorkhabadi, S. M., and Zhang, W. (2020). Assessment of Human Dynamic Gait Stability With a Lower Extremity Assistive Device. *IEEE Trans. Neural Syst. Rehabil. Eng.* 28, 669–678. doi:10.1109/TNSRE.2020.2970207.

Choi, H., Park, Y. J., Seo, K., Lee, J., Lee, S. E., and Shim, Y. (2018). A Multifunctional Ankle Exoskeleton for Mobility Enhancement of Gait-Impaired Individuals and Seniors. *IEEE Robot. Autom. Lett.* 3, 411–418. doi:10.1109/LRA.2017.2734239.

Conner, B. C. B. C., Luque, J., and Lerner, Z. F. Z. F. (2020). Adaptive Ankle Resistance from a Wearable Robotic Device to Improve Muscle Recruitment in Cerebral Palsy. *Ann. Biomed. Eng.* 48, 1309–1321. doi:10.1007/s10439-020-02454-8.

Di Natali, C., Poliero, T., Sposito, M., Graf, E., Bauer, C., Pauli, C., et al. (2019). Design and Evaluation of a Soft Assistive Lower Limb Exoskeleton. *Robotica* 37, 2014–2034. doi:10.1017/S0263574719000067.

Ding, Y., Galiana, I., Asbeck, A. T., De Rossi, S. M. M., Bae, J., Santos, T. R. T., et al. (2017). Biomechanical and Physiological Evaluation of Multi-Joint Assistance With Soft Exosuits. *IEEE Trans. Neural Syst. Rehabil. Eng.* 25, 119–130. doi:10.1109/TNSRE.2016.2523250.

Ding, Y., Galiana, I., Siviy, C., Panizzolo, F. A., and Walsh, C. (2016a). IMU-based iterative control for hip extension assistance with a soft exosuit. in *2016 IEEE International Conference on Robotics and Automation (ICRA)* IEEE International Conference on Robotics and Automation ICRA. (IEEE), 3501–3508. doi:10.1109/ICRA.2016.7487530.

Ding, Y., Kim, M., Kuindersma, S., and Walsh, C. J. (2018). Human-in-the-loop optimization of hip assistance with a soft exosuit during walking. *Sci. Robot.* 3, 1–9. doi:10.1126/scirobotics.aar5438.

Ding, Y., Panizzolo, F. A., Siviy, C., Malcolm, P., Galiana, I., Holt, K. G., et al. (2016b). Effect of timing of hip extension assistance during loaded walking with a soft exosuit. *J. Neuroeng. Rehabil.* 13, 87. doi:10.1186/s12984-016-0196-8.

dos Santos, W. M., Caurin, G. A. P., and Siqueira, A. A. G. (2017). Design and control of an active knee orthosis driven by a rotary Series Elastic Actuator. *Control Eng. Pract.* 58, 307–318. doi:10.1016/j.conengprac.2015.09.008.

Durandau, G., Farina, D., Asín-Prieto, G., Dimbwadyo-Terrer, I., Lerma-Lara, S., Pons, J. L., et al. (2019). Voluntary control of wearable robotic exoskeletons by patients with paresis via neuromechanical modeling. *J. Neuroeng. Rehabil.* 16, 91. doi:10.1186/s12984-019-0559-z.

Dzeladini, F., Wu, A. R., Renjewski, D., Arami, A., Burdet, E., van Asseldonk, E., et al. (2016). Effects of a neuromuscular controller on a powered ankle exoskeleton during human walking. in *2016 6th IEEE International Conference on Biomedical Robotics and Biomechatronics (BioRob)* (IEEE), 617–622. doi:10.1109/BIOROB.2016.7523694.

Fang, Y., and Lerner, Z. F. (2021). Feasibility of Augmenting Ankle Exoskeleton Walking Performance With Step Length Biofeedback in Individuals With Cerebral Palsy. *IEEE Trans. Neural Syst. Rehabil. Eng.* 29, 442–449. doi:10.1109/TNSRE.2021.3055796.

Farris, R. J., Quintero, H. A., and Goldfarb, M. (2011). Preliminary evaluation of a powered lower limb orthosis to aid walking in paraplegic individuals. *IEEE Trans. Neural Syst. Rehabil. Eng.* 19, 652–659. doi:10.1109/TNSRE.2011.2163083.

Fleischer, C., and Hommel, Gü. (2008). A Human--Exoskeleton Interface Utilizing Electromyography. *IEEE Trans. Robot.* 24, 872–882. doi:10.1109/TRO.2008.926860.

Forrester, L. W., Roy, A., Hafer-Macko, C., Krebs, H. I., and Macko, R. F. (2016). Task-specific ankle robotics gait training after stroke: a randomized pilot study. *J. Neuroeng. Rehabil.* 13, 51. doi:10.1186/s12984-016-0158-1.

Galle, S., Malcolm, P., Collins, S. H., and De Clercq, D. (2017). Reducing the metabolic cost of walking with an ankle exoskeleton: interaction between actuation timing and power. *J. Neuroeng. Rehabil.* 14, 1–16. doi:10.1186/s12984-017-0235-0.

Gasparri, G. M., Luque, J., and Lerner, Z. F. (2019). Proportional Joint-Moment Control for Instantaneously Adaptive Ankle Exoskeleton Assistance. *IEEE Trans. Neural Syst. Rehabil. Eng.* 27, 751–759. doi:10.1109/TNSRE.2019.2905979.

Giovacchini, F., Vannetti, F., Fantozzi, M., Cempini, M., Cortese, M., Parri, A., et al. (2015). A light-weight active orthosis for hip movement assistance. *Rob. Auton. Syst.* 73, 123–134. doi:10.1016/j.robot.2014.08.015.

Gomez-Vargas, D., Ballen-Moreno, F., Barria, P., Aguilar, R., Azorín, J. M., Munera, M., et al. (2021). The Actuation System of the Ankle Exoskeleton T-FLEX: First Use Experimental Validation in People with Stroke. *Brain Sci.* 11, 412. doi:10.3390/brainsci11040412.

Gordon, K. E., and Ferris, D. P. (2007). Learning to walk with a robotic ankle exoskeleton. *J. Biomech.* 40, 2636–2644. doi:10.1016/j.jbiomech.2006.12.006.

Grazi, L., Crea, S., Parri, A., Tingfang Yan, Cortese, M., Giovacchini, F., et al. (2015). Gastrocnemius myoelectric control of a robotic hip exoskeleton. in *2015 37th Annual International Conference of the IEEE Engineering in Medicine and Biology Society (EMBC)* (IEEE), 3881–3884. doi:10.1109/EMBC.2015.7319241.

Gui, K., Liu, H., and Zhang, D. (2017). A generalized framework to achieve coordinated admittance control for multi-joint lower limb robotic exoskeleton. in *2017 International Conference on Rehabilitation Robotics (ICORR)* International Conference on Rehabilitation Robotics ICORR., ed. L. Amirabdollahian, F and Burdet, E and Masia (IEEE), 228–233. doi:10.1109/ICORR.2017.8009251.

Han, Y., Zhu, S., Zhou, Y., and Gao, H. (2019). An admittance controller based on assistive torque estimation for a rehabilitation leg exoskeleton. *Intell. Serv. Robot.* 12, 381–391. doi:10.1007/s11370-019-00289-4.

Hassan, M., Kadone, H., Suzuki, K., and Sankai, Y. (2012). Exoskeleton robot control based on cane and body joint synergies. in *2012 IEEE/RSJ International Conference on Intelligent Robots and Systems* IEEE International Conference on Intelligent Robots and Systems. (IEEE), 1609–1614. doi:10.1109/IROS.2012.6386248.

Hassan, M., Kadone, H., Ueno, T., Hada, Y., Sankai, Y., and Suzuki, K. (2018). Feasibility of Synergy-Based Exoskeleton Robot Control in Hemiplegia. *IEEE Trans. Neural Syst. Rehabil. Eng.* 26, 1233–1242. doi:10.1109/TNSRE.2018.2832657.

Haufe, F. L., Kober, A. M., Wolf, P., Riener, R., and Xiloyannis, M. (2021). Learning to walk with a wearable robot in 880 simple steps: a pilot study on motor adaptation. *J. Neuroeng. Rehabil.* 18, 1–14. doi:10.1186/s12984-021-00946-9.

Hidayah, R., Bishop, L., Jin, X., Chamarthy, S., Stein, J., and Agrawal, S. K. (2020). Gait Adaptation Using a Cable-Driven Active Leg Exoskeleton (C-ALEX) With Post-Stroke Participants. *IEEE Trans. Neural Syst. Rehabil. Eng.* 28, 1984–1993. doi:10.1109/TNSRE.2020.3009317.

Horst, R. W. (2009). A bio-robotic leg orthosis for rehabilitation and mobility enhancement. in *2009 Annual International Conference of the IEEE Engineering in Medicine and Biology Society* (IEEE), 5030–5033. doi:10.1109/IEMBS.2009.5333581.

Huo, W., Arnez-Paniagua, V., Ding, G., Amirat, Y., and Mohammed, S. (2019). Adaptive Proxy-Based Controller of an Active Ankle Foot Orthosis to Assist Lower Limb Movements of Paretic Patients. *Robotica* 37, 2147–2164. doi:10.1017/S0263574719000250.

Ishmael, M. K., Tran, M., and Lenzi, T. (2019). ExoProsthetics: Assisting above-knee amputees with a lightweight powered hip exoskeleton. *IEEE Int. Conf. Rehabil. Robot.* 2019-June, 925–930. doi:10.1109/ICORR.2019.8779412.

Jackson, R. W., and Collins, S. H. (2015). An experimental comparison of the relative benefits of work and torque assistance in ankle exoskeletons. *J. Appl. Physiol.* 119, 541–557. doi:10.1152/japplphysiol.01133.2014.

Jackson, R. W., and Collins, S. H. (2019). Heuristic-Based Ankle Exoskeleton Control for Co-Adaptive Assistance of Human Locomotion. *IEEE Trans. Neural Syst. Rehabil. Eng.* 27, 2059–2069. doi:10.1109/TNSRE.2019.2936383.

Jin, S., Iwamoto, N., Hashimoto, K., and Yamamoto, M. (2017). Experimental Evaluation of Energy Efficiency for a Soft Wearable Robotic Suit. *IEEE Trans. Neural Syst. Rehabil. Eng.* 25, 1192–1201. doi:10.1109/TNSRE.2016.2613886.

Jin, X., Cui, X., and Agrawal, S. K. (2015). Design of a cable-driven active leg exoskeleton (C-ALEX) and gait training experiments with human subjects. *Proc. - IEEE Int. Conf. Robot. Autom.* 2015-June, 5578–5583. doi:10.1109/ICRA.2015.7139979.

Jin, X., Prado, A., and Agrawal, S. K. (2018). Retraining of Human Gait - Are Lightweight Cable-Driven Leg Exoskeleton Designs Effective? *IEEE Trans. Neural Syst. Rehabil. Eng.* 26, 847–855. doi:10.1109/TNSRE.2018.2815656.

Kang, I., Hsu, H., and Young, A. (2019). The Effect of Hip Assistance Levels on Human Energetic Cost Using Robotic Hip Exoskeletons. *IEEE Robot. Autom. Lett.* 4, 430–437. doi:10.1109/lra.2019.2890896.

Kang, I., Kunapuli, P., and Young, A. J. (2020). Real-Time Neural Network-Based Gait Phase Estimation Using a Robotic Hip Exoskeleton. *IEEE Trans. Med. Robot. Bionics* 2, 28–37. doi:10.1109/TMRB.2019.2961749.

Kao, P.-C., Lewis, C. L., and Ferris, D. P. (2010). Invariant ankle moment patterns when walking with and without a robotic ankle exoskeleton. *J. Biomech.* 43, 203–209. doi:10.1016/j.jbiomech.2009.09.030.

Kawamoto, H., Hayashi, T., Sakurai, T., Eguchi, K., and Sankai, Y. (2009). Development of single leg version of HAL for hemiplegia. *Proc. 31st Annu. Int. Conf. IEEE Eng. Med. Biol. Soc. Eng. Futur. Biomed. EMBC 2009*, 5038–5043. doi:10.1109/IEMBS.2009.5333698.

Kawamoto, H., Kadone, H., Sakurai, T., and Sankai, Y. (2015). Modification of hemiplegic compensatory gait pattern by symmetry-based motion controller of HAL. in *2015 37th Annual International Conference of the IEEE Engineering in Medicine and Biology Society (EMBC)* (United States: IEEE), 4803–4807. doi:10.1109/EMBC.2015.7319468.

Kawamoto, H., Kandone, H., Sakurai, T., Ariyasu, R., Ueno, Y., Eguchi, K., et al. (2014). Development of an assist controller with robot suit HAL for hemiplegic patients using motion data on the unaffected side. in *2014 36th Annual International Conference of the IEEE Engineering in Medicine and Biology Society* (United States: IEEE), 3077–3080. doi:10.1109/EMBC.2014.6944273.

Kawamoto, H., and Sankai, Y. (2005). Power assist method based on Phase Sequence and muscle force condition for HAL. *Adv. Robot.* 19, 717–734. doi:10.1163/1568553054455103.

Kawamoto, H., Taal, S., Niniss, H., Hayashi, T., Kamibayashi, K., Eguchi, K., et al. (2010). Voluntary motion support control of Robot Suit HAL triggered by bioelectrical signal for hemiplegia. *2010 Annu. Int. Conf. IEEE Eng. Med. Biol. Soc. EMBC’10*, 462–466. doi:10.1109/IEMBS.2010.5626191.

Kim, J.-H., Shim, M., Ahn, D. H., Son, B. J., Kim, S.-Y., Kim, D. Y., et al. (2015). Design of a Knee Exoskeleton Using Foot Pressure and Knee Torque Sensors. *Int. J. Adv. Robot. Syst.* 12, 112. doi:10.5772/60782.

Kim, J., Hwang, S., Sohn, R., Lee, Y., and Kim, Y. (2011). Development of an active ankle foot orthosis to prevent foot drop and toe drag in hemiplegic patients: A preliminary study. *Appl. Bionics Biomech.* 8, 377–384. doi:10.3233/ABB-2011-0008.

Kim, J., Lee, G., Heimgartner, R., Revi, D. A., Karavas, N., Nathanson, D., et al. (2019). Reducing the metabolic rate of walking and running with a versatile, portable exosuit. *Science (80-. ).* 365, 668–672. doi:10.1126/science.aav7536.

Kim, S. J., Na, Y., Lee, D. Y., Chang, H., and Kim, J. (2020). Pneumatic AFO Powered by a Miniature Custom Compressor for Drop Foot Correction. *IEEE Trans. Neural Syst. Rehabil. Eng.* 28, 1781–1789. doi:10.1109/TNSRE.2020.3003860.

Kinnaird, C. R., and Ferris, D. P. (2009). Medial gastrocnemius myoelectric control of a robotic ankle exoskeleton. *IEEE Trans. Neural Syst. Rehabil. Eng.* 17, 31–37. doi:10.1109/TNSRE.2008.2008285.

Knaepen, K., Beyl, P., Duerinck, S., Hagman, F., Lefeber, D., and Meeusen, R. (2014). Human–Robot Interaction: Kinematics and Muscle Activity Inside a Powered Compliant Knee Exoskeleton. *IEEE Trans. Neural Syst. Rehabil. Eng.* 22, 1128–1137. doi:10.1109/TNSRE.2014.2324153.

Koller, J. R., David Remy, C., and Ferris, D. P. (2017). Comparing neural control and mechanically intrinsic control of powered ankle exoskeletons. *IEEE Int. Conf. Rehabil. Robot.*, 294–299. doi:10.1109/ICORR.2017.8009262.

Koller, J. R., Jacobs, D. A., Ferris, D. P., and Remy, C. D. (2015). Learning to walk with an adaptive gain proportional myoelectric controller for a robotic ankle exoskeleton. *J. Neuroeng. Rehabil.* 12, 97. doi:10.1186/s12984-015-0086-5.

Lai, W.-Y., Ma, H., Liao, W.-H., Fong, D. T.-P., and Chan, K.-M. (2013). HIP-KNEE control for gait assistance with Powered Knee Orthosis. in *2013 IEEE International Conference on Robotics and Biomimetics (ROBIO)* (IEEE), 762–767. doi:10.1109/ROBIO.2013.6739554.

Lee, D., Kwak, E. C., McLain, B. J., Kang, I., and Young, A. J. (2020). Effects of Assistance during Early Stance Phase Using a Robotic Knee Orthosis on Energetics, Muscle Activity, and Joint Mechanics during Incline and Decline Walking. *IEEE Trans. Neural Syst. Rehabil. Eng.* 28, 914–923. doi:10.1109/TNSRE.2020.2972323.

Lee, G., Ding, Y., Bujanda, I. G., Karavas, N., Zhou, Y. M., and Walsh, C. J. (2017a). Improved assistive profile tracking of soft exosuits for walking and jogging with off-board actuation. in *2017 IEEE/RSJ International Conference on Intelligent Robots and Systems (IROS)* (IEEE), 1699–1706. doi:10.1109/IROS.2017.8205981.

Lee, H.-J., Lee, S.-H., Seo, K., Lee, M., Chang, W. H., Choi, B.-O., et al. (2019). Training for Walking Efficiency With a Wearable Hip-Assist Robot in Patients With Stroke. *Stroke* 50, 3545–3552. doi:10.1161/STROKEAHA.119.025950.

Lee, H. J., Lee, S., Chang, W. H., Seo, K., Shim, Y., Choi, B. O., et al. (2017b). A Wearable Hip Assist Robot Can Improve Gait Function and Cardiopulmonary Metabolic Efficiency in Elderly Adults. *IEEE Trans. Neural Syst. Rehabil. Eng.* 25, 1549–1557. doi:10.1109/TNSRE.2017.2664801.

Lee, S., Crea, S., Malcolm, P., Galiana, I., Asbeck, A., and Walsh, C. (2016). Controlling negative and positive power at the ankle with a soft exosuit. in *2016 IEEE International Conference on Robotics and Automation (ICRA)* (IEEE), 3509–3515. doi:10.1109/ICRA.2016.7487531.

Lee, S. H., Lee, H. J., Chang, W. H., Choi, B. O., Lee, J., Kim, J., et al. (2017c). Gait performance and foot pressure distribution during wearable robot-assisted gait in elderly adults. *J. Neuroeng. Rehabil.* 14, 1–10. doi:10.1186/s12984-017-0333-z.

Lenzi, T., Carrozza, M. C., and Agrawal, S. K. (2013). Powered Hip Exoskeletons Can Reduce the User’s Hip and Ankle Muscle Activations During Walking. *IEEE Trans. Neural Syst. Rehabil. Eng.* 21, 938–948. doi:10.1109/TNSRE.2013.2248749.

Lerner, Z. F., Damiano, D. L., and Bulea, T. C. (2017a). Relationship between assistive torque and knee biomechanics during exoskeleton walking in individuals with crouch gait. in *2017 International Conference on Rehabilitation Robotics (ICORR)* (United States: IEEE), 491–497. doi:10.1109/ICORR.2017.8009296.

Lerner, Z. F., Damiano, D. L., Park, H.-S., Gravunder, A. J., and Bulea, T. C. (2017b). A Robotic Exoskeleton for Treatment of Crouch Gait in Children with Cerebral Palsy: Design and Initial Application. *IEEE Trans. Neural Syst. Rehabil. Eng.* 25, 650–659. doi:10.1109/TNSRE.2016.2595501.

Lerner, Z. F., Gasparri, G. M., Bair, M. O., Lawson, J. L., Luque, J., Harvey, T. A., et al. (2018). An untethered ankle exoskeleton improves walking economy in a pilot study of individuals with cerebral palsy. *IEEE Trans. Neural Syst. Rehabil. Eng.* 26, 1985–1993. doi:10.1109/TNSRE.2018.2870756.

Li, D. Y., Becker, A., Shorter, K. A., Bretl, T., and Hsiao-Wecksler, E. T. (2011). Estimating system state during human walking with a powered ankle-foot orthosis. *IEEE/ASME Trans. Mechatronics* 16, 835–844. doi:10.1109/TMECH.2011.2161769.

Lim, B., Kim, K., Lee, J., Jang, J., and Shim, Y. (2015). An event-driven control to achieve adaptive walking assist with gait primitives. *IEEE Int. Conf. Intell. Robot. Syst.* 2015-Decem, 5870–5875. doi:10.1109/IROS.2015.7354211.

Liu, X., and Wang, Q. (2020). Real-Time Locomotion Mode Recognition and Assistive Torque Control for Unilateral Knee Exoskeleton on Different Terrains. *IEEE/ASME Trans. Mechatronics* 25, 2722–2732. doi:10.1109/TMECH.2020.2990668.

Lora-Millan, J. S., Moreno, J. C., and Rocon, E. (2020). Assessment of gait symmetry, torque interaction and muscular response due to the unilateral assistance provided by an active knee orthosis in healthy subjects. in *2020 8th IEEE RAS/EMBS International Conference for Biomedical Robotics and Biomechatronics (BioRob)* (IEEE), 229–234. doi:10.1109/BioRob49111.2020.9224414.

Ma, H., Zhong, C., Chen, B., Chan, K. M., and Liao, W. H. (2018). User-Adaptive Assistance of Assistive Knee Braces for Gait Rehabilitation. *IEEE Trans. Neural Syst. Rehabil. Eng.* 26, 1994–2005. doi:10.1109/TNSRE.2018.2868693.

Malcolm, P., Galle, S., Van Den Berghe, P., and De Clercq, D. (2018). Exoskeleton assistance symmetry matters: Unilateral assistance reduces metabolic cost, but relatively less than bilateral assistance. *J. Neuroeng. Rehabil.* 15. doi:10.1186/s12984-018-0381-z.

Martínez, A., Durrough, C., Goldfarb, M., Martinez, A., Durrough, C., and Goldfarb, M. (2020). A Single-Joint Implementation of Flow Control: Knee Joint Walking Assistance for Individuals with Mobility Impairment. *IEEE Trans. Neural Syst. Rehabil. Eng.* 28, 934–942. doi:10.1109/TNSRE.2020.2977339.

Martinez, A., Lawson, B., Durrough, C., and Goldfarb, M. (2019). A Velocity-Field-Based Controller for Assisting Leg Movement During Walking With a Bilateral Hip and Knee Lower Limb Exoskeleton. *IEEE Trans. Robot.* 35, 307–316. doi:10.1109/TRO.2018.2883819.

Martinez, A., Lawson, B., and Goldfarb, M. (2018). A Controller for Guiding Leg Movement During Overground Walking With a Lower Limb Exoskeleton. *IEEE Trans. Robot.* 34, 183–193. doi:10.1109/TRO.2017.2768035.

McCain, E. M., Dick, T. J. M., Giest, T. N., Nuckols, R. W., Lewek, M. D., Saul, K. R., et al. (2019). Mechanics and energetics of post-stroke walking aided by a powered ankle exoskeleton with speed-adaptive myoelectric control. *J. Neuroeng. Rehabil.* 16, 57. doi:10.1186/s12984-019-0523-y.

Meuleman, J., van Asseldonk, E., van Oort, G., Rietman, H., and van der Kooij, H. (2016). LOPES II—Design and Evaluation of an Admittance Controlled Gait Training Robot With Shadow-Leg Approach. *IEEE Trans. Neural Syst. Rehabil. Eng.* 24, 352–363. doi:10.1109/TNSRE.2015.2511448.

Mishra, A., Ghosh, R., Coscia, M., Kukreja, S., Chisari, C., Micera, S., et al. (2014). A neurally inspired robotic control algorithm for gait rehabilitation in hemiplegic stroke patients. in *5th IEEE RAS/EMBS International Conference on Biomedical Robotics and Biomechatronics* Proceedings of the IEEE RAS-EMBS International Conference on Biomedical Robotics and Biomechatronics. (IEEE), 650–655. doi:10.1109/BIOROB.2014.6913852.

Mizukami, N., Takeuchi, S., Tetsuya, M., Tsukahara, A., Yoshida, K., Matsushima, A., et al. (2018). Effect of the Synchronization-Based Control of a Wearable Robot Having a Non-Exoskeletal Structure on the Hemiplegic Gait of Stroke Patients. *IEEE Trans. Neural Syst. Rehabil. Eng.* 26, 1011–1016. doi:10.1109/TNSRE.2018.2817647.

Mooney, L. M. L. M., and Herr, H. M. H. M. (2016). Biomechanical walking mechanisms underlying the metabolic reduction caused by an autonomous exoskeleton. *J. Neuroeng. Rehabil.* 13, 4. doi:10.1186/s12984-016-0111-3.

Mooney, L. M., Rouse, E. J., and Herr, H. M. (2014a). Autonomous exoskeleton reduces metabolic cost of human walking. *J. Neuroeng. Rehabil.* 11. doi:10.1186/1743-0003-11-151.

Mooney, L. M., Rouse, E. J., and Herr, H. M. (2014b). Autonomous exoskeleton reduces metabolic cost of human walking during load carriage. *J. Neuroeng. Rehabil.* 11, 80. doi:10.1186/1743-0003-11-80.

Mooney, L. M., Rouse, E. J., and Herr, H. M. (2014c). Autonomous exoskeleton reduces metabolic cost of walking. in *2014 36th Annual International Conference of the IEEE Engineering in Medicine and Biology Society* (England: IEEE), 3065–3068. doi:10.1109/EMBC.2014.6944270.

Nguyen, T., Komeda, T., Miyoshi, T., and Ota, L. (2013). The powered gait training system using feedback from own walking information. in *2013 ISSNIP Biosignals and Biorobotics Conference: Biosignals and Robotics for Better and Safer Living (BRC)* (IEEE), 1–5. doi:10.1109/BRC.2013.6487529.

Nilsson, A., Vreede, K., Häglund, V., Kawamoto, H., Sankai, Y., and Borg, J. (2014). Gait training early after stroke with a new exoskeleton – the hybrid assistive limb: a study of safety and feasibility. *J. Neuroeng. Rehabil.* 11, 92. doi:10.1186/1743-0003-11-92.

Nunes, P. F., dos Santos, W. M., and Siqueira, A. A. G. (2018). Control Strategy Based on Kinetic Motor Primitives for Lower Limbs Exoskeletons. *IFAC-PapersOnLine* 51, 402–406. doi:10.1016/j.ifacol.2019.02.003.

Orekhov, G., Fang, Y., Cuddeback, C. F., and Lerner, Z. F. (2021). Usability and performance validation of an ultra-lightweight and versatile untethered robotic ankle exoskeleton. *J. Neuroeng. Rehabil.* 18, 1–16. doi:10.1186/s12984-021-00954-9.

Orekhov, G., Fang, Y., Luque, J., and Lerner, Z. F. (2020). Ankle Exoskeleton Assistance Can Improve Over-Ground Walking Economy in Individuals with Cerebral Palsy. *IEEE Trans. Neural Syst. Rehabil. Eng.* doi:10.1109/TNSRE.2020.2965029.

Oymagil, A. M., Hitt, J. K., Sugar, T., and Fleeger, J. (2007). Control of a Regenerative Braking Powered Ankle Foot Orthosis. in *2007 IEEE 10th International Conference on Rehabilitation Robotics* (IEEE), 28–34. doi:10.1109/ICORR.2007.4428402.

Peng, Z., Luo, R., Huang, R., Yu, T., Hu, J., Shi, K., et al. (2020). Data-Driven Optimal Assistance Control of a Lower Limb Exoskeleton for Hemiplegic Patients. *Front. Neurorobot.* 14. doi:10.3389/fnbot.2020.00037.

Ronsse, R., Lenzi, T., Vitiello, N., Koopman, B., van Asseldonk, E., De Rossi, S. M. M., et al. (2011). Oscillator-based assistance of cyclical movements: model-based and model-free approaches. *Med. Biol. Eng. Comput.* 49, 1173–1185. doi:10.1007/s11517-011-0816-1.

Roy, A., Krebs, H. I., Barton, J. E., Macko, R. F., and Forrester, L. W. (2013). Anklebot-assisted locomotor training after stroke: A novel deficit-adjusted control approach. in *2013 IEEE International Conference on Robotics and Automation* (IEEE), 2175–2182. doi:10.1109/ICRA.2013.6630869.

Ruiz Garate, V., Parri, A., Yan, T., Munih, M., Molino Lova, R., Vitiello, N., et al. (2016). Walking Assistance Using Artificial Primitives: A Novel Bioinspired Framework Using Motor Primitives for Locomotion Assistance Through a Wearable Cooperative Exoskeleton. *IEEE Robot. Autom. Mag.* 23, 83–95. doi:10.1109/MRA.2015.2510778.

Ruiz Garate, V., Parri, A., Yan, T., Munih, M., Molino Lova, R., Vitiello, N., et al. (2017). Experimental Validation of Motor Primitive-Based Control for Leg Exoskeletons during Continuous Multi-Locomotion Tasks. *Front. Neurorobot.* 11. doi:10.3389/fnbot.2017.00015.

Sanz-Morere, C. B., Fantozzi, M., Parri, A., Giovacchini, F., Baldoni, A., Crea, S., et al. (2018). A Bioinspired Control Strategy for the CYBERLEGs Knee-Ankle-Foot Orthosis: Feasibility Study with Lower-Limb Amputees. in *2018 7th IEEE International Conference on Biomedical Robotics and Biomechatronics (Biorob)* (IEEE), 503–508. doi:10.1109/BIOROB.2018.8487692.

Sawicki, G. S., and Ferris, D. P. (2008). Mechanics and energetics of level walking with powered ankle exoskeletons. *J. Exp. Biol.* 211, 1402–1413. doi:10.1242/jeb.009241.

Sawicki, G. S., and Ferris, D. P. (2009). A pneumatically powered knee-ankle-foot orthosis (KAFO) with myoelectric activation and inhibition. *J. Neuroeng. Rehabil.* 6, 1–16. doi:10.1186/1743-0003-6-23.

Sczesny-Kaiser, M., Trost, R., Aach, M., Schildhauer, T. A., Schwenkreis, P., and Tegenthoff, M. (2019). A Randomized and Controlled Crossover Study Investigating the Improvement of Walking and Posture Functions in Chronic Stroke Patients Using HAL Exoskeleton - The HALESTRO Study (HAL-Exoskeleton STROke Study). *Front. Neurosci.* 13, 1–13. doi:10.3389/fnins.2019.00259.

Seo, K., Lee, J., Lee, Y., Ha, T., and Shim, Y. (2016). Fully autonomous hip exoskeleton saves metabolic cost of walking. *Proc. - IEEE Int. Conf. Robot. Autom.* 2016-June, 4628–4635. doi:10.1109/ICRA.2016.7487663.

Shamaei, K., Cenciarini, M., Adams, A. A., Gregorczyk, K. N., Schiffman, J. M., and Dollar, A. M. (2014a). Design and Evaluation of a Quasi-Passive Knee Exoskeleton for Investigation of Motor Adaptation in Lower Extremity Joints. *IEEE Trans. Biomed. Eng.* 61, 1809–1821. doi:10.1109/TBME.2014.2307698.

Shamaei, K., Cenciarini, M., Adams, A. A., Gregorczyk, K. N., Schiffman, J. M., and Dollar, A. M. (2015). Biomechanical Effects of Stiffness in Parallel With the Knee Joint During Walking. *IEEE Trans. Biomed. Eng.* 62, 2389–2401. doi:10.1109/TBME.2015.2428636.

Shamaei, K., Napolitano, P. C., and Dollar, A. M. (2013). A quasi-passive compliant stance control Knee-Ankle-Foot Orthosis. in *2013 IEEE 13th International Conference on Rehabilitation Robotics (ICORR)* (United States: IEEE), 1–6. doi:10.1109/ICORR.2013.6650471.

Shamaei, K., Napolitano, P. C., and Dollar, A. M. (2014b). Design and Functional Evaluation of a Quasi-Passive Compliant Stance Control Knee–Ankle–Foot Orthosis. *IEEE Trans. Neural Syst. Rehabil. Eng.* 22, 258–268. doi:10.1109/TNSRE.2014.2305664.

Sharbafi, M. A., Barazesh, H., Iranikhah, M., and Seyfarth, A. (2018). Leg Force Control Through Biarticular Muscles for Human Walking Assistance. *Front. Neurorobot.* 12, 1–13. doi:10.3389/fnbot.2018.00039.

Shorter, K. A., Kogler, G. F., Loth, E., Durfee, W. K., and Hsiao-Wecksler, E. T. (2011). A portable powered ankle-foot orthosis for rehabilitation. *J. Rehabil. Res. Dev.* 48, 459. doi:10.1682/JRRD.2010.04.0054.

Siviy, C., Bae, J., Baker, L., Porciuncula, F., Baker, T., Ellis, T. D., et al. (2020). Offline Assistance Optimization of a Soft Exosuit for Augmenting Ankle Power of Stroke Survivors During Walking. *IEEE Robot. Autom. Lett.* 5, 828–835. doi:10.1109/LRA.2020.2965072.

Sridar, S., Qiao, Z., Muthukrishnan, N., Zhang, W., and Polygerinos, P. (2018). A soft-inflatable exosuit for knee rehabilitation: Assisting swing phase during walking. *Front. Robot. AI* 5, 1–9. doi:10.3389/frobt.2018.00044.

Sridar, S., Qiao, Z., Rascon, A., Biemond, A., Beltran, A., Maruyama, T., et al. (2020). Evaluating Immediate Benefits of Assisting Knee Extension with a Soft Inflatable Exosuit. *IEEE Trans. Med. Robot. Bionics* 2, 216–225. doi:10.1109/TMRB.2020.2988305.

Srivastava, S., Kao, P.-C. C., Kim, S. H., Stegall, P., Zanotto, D., Higginson, J. S., et al. (2015). Assist-as-Needed Robot-Aided Gait Training Improves Walking Function in Individuals Following Stroke. *IEEE Trans. Neural Syst. Rehabil. Eng.* 23, 956–963. doi:10.1109/TNSRE.2014.2360822.

Steele, K. M., Jackson, R. W., Shuman, B. R., and Collins, S. H. (2017). Muscle recruitment and coordination with an ankle exoskeleton. *J. Biomech.* 59, 50–58. doi:10.1016/j.jbiomech.2017.05.010.

Stein, J., Bishop, L., Stein, D. J., and Wong, C. K. (2014). Gait training with a robotic leg brace after stroke: A randomized controlled pilot study. *Am. J. Phys. Med. Rehabil.* 93, 987–994. doi:10.1097/PHM.0000000000000119.

Takahashi, K. Z., Lewek, M. D., and Sawicki, G. S. (2015). A neuromechanics-based powered ankle exoskeleton to assist walking post-stroke: A feasibility study. *J. Neuroeng. Rehabil.* 12. doi:10.1186/s12984-015-0015-7.

Talatian, H., Karami, M., Moradi, H., and Vossoughi, G. (2021). Design and Implementation of an Intelligent Control System for a Lower-Limb Exoskeleton to Reduce Human Energy Consumption. in *2021 10th International Conference on Modern Circuits and Systems Technologies, MOCAST 2021* (IEEE), 1–4. doi:10.1109/MOCAST52088.2021.9493401.

Tamburella, F., Tagliamonte, N. L., Pisotta, I., Masciullo, M., Arquilla, M., Van Asseldonk, E. H. F., et al. (2020). Neuromuscular Controller Embedded in a Powered Ankle Exoskeleton: Effects on Gait, Clinical Features and Subjective Perspective of Incomplete Spinal Cord Injured Subjects. *IEEE Trans. Neural Syst. Rehabil. Eng.* 28, 1157–1167. doi:10.1109/TNSRE.2020.2984790.

Tan, C. K., Kadone, H., Watanabe, H., Marushima, A., Hada, Y., Yamazaki, M., et al. (2020). Differences in Muscle Synergy Symmetry Between Subacute Post-stroke Patients With Bioelectrically-Controlled Exoskeleton Gait Training and Conventional Gait Training. *Front. Bioeng. Biotechnol.* 8. doi:10.3389/fbioe.2020.00770.

Tan, C. K., Kadone, H., Watanabe, H., Marushima, A., Yamazaki, M., Sankai, Y., et al. (2018). Lateral Symmetry of Synergies in Lower Limb Muscles of Acute Post-stroke Patients After Robotic Intervention. *Front. Neurosci.* 12, 1–13. doi:10.3389/fnins.2018.00276.

Tricomi, E., Lotti, N., Missiroli, F., Zhang, X., Xiloyannis, M., Muller, T., et al. (2021). Underactuated Soft Hip Exosuit Based on Adaptive Oscillators to Assist Human Locomotion. *IEEE Robot. Autom. Lett.* 3766, 1–1. doi:10.1109/lra.2021.3136240.

Tsukahara, A., and Hashimoto, M. (2016). Pilot study of single-legged walking support using wearable robot based on synchronization control for stroke patients. in *2016 IEEE International Conference on Robotics and Biomimetics, ROBIO 2016* (Division of Smart Textiles, Institute for Fiber Engineering (IFES), Interdisciplinary Cluster for Cutting Edge Research (ICCER), Shinshu University, 3-15-1 Tokida, Ueda, Nagano, Japan), 886–891. doi:10.1109/ROBIO.2016.7866436.

Unluhisarcikli, O., Pietrusinski, M., Weinberg, B., Bonato, P., and Mavroidis, C. (2011). Design and control of a robotic lower extremity exoskeleton for gait rehabilitation. in *2011 IEEE International Conference on Intelligent Robots and Systems* (Department of Mechanical and Industrial Engineering, Northeastern University, Boston, MA 02115, United States), 4893–4898. doi:10.1109/IROS.2011.6094973.

Vallery, H., and Buss, M. (2006). Complementary limb motion estimation based on interjoint coordination using principal components analysis. *Proc. IEEE Int. Conf. Control Appl.*, 933–938. doi:10.1109/CACSD-CCA-ISIC.2006.4776770.

Vallery, H., Ekkelenkamp, R., Buss, M., and Van Kooij, H. Der (2007). Complementary limb motion estimation based on interjoint coordination: Experimental evaluation. in *2007 IEEE 10th International Conference on Rehabilitation Robotics, ICORR’07* (IEEE), 798–803. doi:10.1109/ICORR.2007.4428516.

Vallery, H., Van Asseldonk, E. H. F., Buss, M., and Van Der Kooij, H. (2009). Reference trajectory generation for rehabilitation robots: Complementary limb motion estimation. *IEEE Trans. Neural Syst. Rehabil. Eng.* 17, 23–30. doi:10.1109/TNSRE.2008.2008278.

van Dijk, W., Meijneke, C., and van der Kooij, H. (2017). Evaluation of the Achilles Ankle Exoskeleton. *IEEE Trans. Neural Syst. Rehabil. Eng.* 25, 151–160. doi:10.1109/TNSRE.2016.2527780.

Veneman, J. F., Kruidhof, R., Hekman, E. E. G., Ekkelenkamp, R., Van Asseldonk, E. H. F., and van der Kooij, H. (2007). Design and evaluation of the LOPES exoskeleton robot for interactive gait rehabilitation. *IEEE Trans. Neural Syst. Rehabil. Eng.* 15, 379–386. doi:10.1109/TNSRE.2003.818185.

Villa-Parra, A., Delisle-Rodriguez, D., Souza Lima, J., Frizera-Neto, A., and Bastos, T. (2017). Knee Impedance Modulation to Control an Active Orthosis Using Insole Sensors. *Sensors* 17, 2751. doi:10.3390/s17122751.

Wang, W. J., Li, J., Li, W. Da, and Sun, L. N. (2013). An Echo-Based Gait Phase Determination Method of Lower Limb Prosthesis. *Adv. Mater. Res.* 706–708, 629–634. doi:10.4028/www.scientific.net/AMR.706-708.629.

Ward, J., Sugar, T., Boehler, A., Standeven, J., and Engsberg, J. R. (2011). Stroke Survivors’ Gait Adaptations to a Powered Ankle-Foot Orthosis. *Adv. Robot.* 25, 1879–1901. doi:10.1163/016918611X588907.

Watanabe, H., Marushima, A., Kadone, H., Ueno, T., Shimizu, Y., Kubota, S., et al. (2020). Effects of Gait Treatment With a Single-Leg Hybrid Assistive Limb System After Acute Stroke: A Non-randomized Clinical Trial. *Front. Neurosci.* 13. doi:10.3389/fnins.2019.01389.

Watanabe, H., Tanaka, N., Inuta, T., Saitou, H., and Yanagi, H. (2014). Locomotion improvement using a hybrid assistive limb in recovery phase stroke patients: a randomized controlled pilot study. *Arch. Phys. Med. Rehabil.* 95, 2006–2012. doi:10.1016/j.apmr.2014.07.002.

Wehbi, F. el zahraa, Huo, W., Amirat, Y., Rafei, M. El, Khalil, M., and Mohammed, S. (2017). Active impedance control of a knee-joint orthosis during swing phase. in *2017 International Conference on Rehabilitation Robotics (ICORR)* (IEEE), 435–440. doi:10.1109/ICORR.2017.8009286.

Wei, D., Li, Z., Wei, Q., Su, H., Song, B. B., He, W., et al. (2019). Human-in-the-Loop Control Strategy of Unilateral Exoskeleton Robots for Gait Rehabilitation. *IEEE Trans. Cogn. Dev. Syst.* 13, 1–1. doi:10.1109/TCDS.2019.2954289.

Winfree, K. N., Stegall, P., and Agrawal, S. K. (2011). Design of a minimally constraining, passively supported gait training exoskeleton: ALEX II. *IEEE Int. Conf. Rehabil. Robot.* 2011, 5975499. doi:10.1109/ICORR.2011.5975499.

Witte, K. A., Zhang, J., Jackson, R. W., and Collins, S. H. (2015). Design of two lightweight, high-bandwidth torque-controlled ankle exoskeletons. in *Proceedings - IEEE International Conference on Robotics and Automation* (Dept. Mechanical Engineering, Carnegie Mellon University, 5000 Forbes Ave., Pittsburgh, PA, United States), 1223–1228. doi:10.1109/ICRA.2015.7139347.

Wong, C. K., Bishop, L., and Stein, J. (2012). A wearable robotic knee orthosis for gait training. *Prosthetics Orthot. Int.* 36, 113–120. doi:10.1177/0309364611428235.

Wu, Q., Wang, X., Du, F., and Zhang, X. (2015). Design and control of a powered hip exoskeleton for walking assistance. *Int. J. Adv. Robot. Syst.* 12. doi:10.5772/59757.

Xia, H., Kwon, J., Pathak, P., Ahn, J., Shull, P. B., and Park, Y.-L. (2020). Design of A Multi-Functional Soft Ankle Exoskeleton for Foot-Drop Prevention, Propulsion Assistance, and Inversion/Eversion Stabilization. in *2020 8th IEEE RAS/EMBS International Conference for Biomedical Robotics and Biomechatronics (BioRob)* (IEEE), 118–123. doi:10.1109/BioRob49111.2020.9224420.

Xie, L., and Huang, L. (2019). Wirerope-driven exoskeleton to assist lower-limb rehabilitation of hemiplegic patients by using motion capture. *Assem. Autom.* 40, 48–54. doi:10.1108/AA-11-2018-0221.

Xu, D., Liu, X., and Wang, Q. (2019). Knee Exoskeleton Assistive Torque Control Based on Real-Time Gait Event Detection. *IEEE Trans. Med. Robot. Bionics* 1, 158–168. doi:10.1109/TMRB.2019.2930352.

Yan, T., Parri, A., Fantozzi, M., Cortese, M., Muscolo, M., Cempini, M., et al. (2015). A novel adaptive oscillators-based control for a powered multi-joint lower-limb orthosis. in *IEEE International Conference on Rehabilitation Robotics* doi:10.1109/ICORR.2015.7281230.

Yeung, L.-F., Lau, C. C. Y., Lai, C. W. K., Soo, Y. O. Y., Chan, M.-L., and Tong, R. K. Y. (2021). Effects of wearable ankle robotics for stair and over-ground training on sub-acute stroke: a randomized controlled trial. *J. Neuroeng. Rehabil.* 18, 19. doi:10.1186/s12984-021-00814-6.

Yeung, L.-F., Ockenfeld, C., Pang, M.-K., Wai, H.-W., Soo, O.-Y., Li, S.-W., et al. (2017). Design of an exoskeleton ankle robot for robot-assisted gait training of stroke patients. *IEEE Int. Conf. Rehabil. Robot.* 2017, 211–215. doi:10.1109/ICORR.2017.8009248.

Young, A. J., Foss, J., Gannon, H., and Ferris, D. P. (2017). Influence of power delivery timing on the energetics and biomechanics of humans wearing a hip exoskeleton. *Front. Bioeng. Biotechnol.* 5, 1–11. doi:10.3389/fbioe.2017.00004.

Zanotto, D., Stegall, P., and Agrawal, S. K. (2014). Adaptive Assist-As-Needed Controller to Improve Gait Symmetry in Robot-Assisted Gait Training. in *2014 IEEE International Conference on Robotics & Automation (ICRA)* IEEE International Conference on Robotics and Automation ICRA., 724–729.

Zhang, B., Wang, S., Zhou, M., and Xu, W. (2021). An adaptive framework of real-time continuous gait phase variable estimation for lower-limb wearable robots. *Rob. Auton. Syst.* 143. doi:10.1016/j.robot.2021.103842.

Zhang, C., Liu, G., Li, C., Zhao, J., Yu, H., and Zhu, Y. (2016). Development of a lower limb rehabilitation exoskeleton based on real-time gait detection and gait tracking. *Adv. Mech. Eng.* 8, 168781401562798. doi:10.1177/1687814015627982.

Zhang, J., Fiers, P., Witte, K. A., Jackson, R. W., Poggensee, K. L., Atkeson, C. G., et al. (2017). Human-in-the-loop optimization of exoskeleton assistance during walking. *Science (80-. ).* 356, 1280–1284. doi:10.1126/science.aal5054.

Zhao, G., Sharbafi, M., Vlutters, M., Van Asseldonk, E., and Seyfarth, A. (2017). Template model inspired leg force feedback based control can assist human walking. *IEEE Int. Conf. Rehabil. Robot.*, 473–478. doi:10.1109/ICORR.2017.8009293.

Zhou, Z., Liao, Y., Wang, C., and Wang, Q. (2016). Preliminary evaluation of gait assistance during treadmill walking with a light-weight bionic knee exoskeleton. in *2016 IEEE International Conference on Robotics and Biomimetics (ROBIO)* (IEEE), 1173–1178. doi:10.1109/ROBIO.2016.7866484.
